# Nickel-Catalyzed Carbonylative Cyclization of Bromodifluoroacetamides with Arylboronic Acids toward δ‑Lactams

**DOI:** 10.1021/acs.orglett.5c03212

**Published:** 2025-08-28

**Authors:** Hucheng Ma, Chen-Yang Hou, Yuting Jiang, Xinxin Qi, Xiao-Feng Wu

**Affiliations:** † School of Chemistry and Chemical Engineering, Key Laboratory of Surface & Interface Science of Polymer Materials of Zhejiang Province, 12646 Zhejiang Sci-Tech University, Hangzhou, Zhejiang 310018, People’s Republic of China; ‡ Dalian National Laboratory for Clean Energy, Dalian Institute of Chemical Physics, Chinese Academy of Sciences, Dalian, Liaoning 116023, China; § 28392 Leibniz-Institut für Katalyse e.V., Albert-Einstein-Straße 29a, Rostock 18059, Germany

## Abstract

A nickel-catalyzed radical cyclization/carbonylation reaction of bromodifluoroacetamides with arylboronic acids has been explored. This strategy allows for the synthesis of δ-lactams bearing both *gem*-difluoro and carbonyl groups through a sequential single-electron transfer, 6-*exo*-*trig* cyclization, and carbonyl insertion process. This method proceeds under mild reaction conditions with good functional group compatibility, enabling the preparation of various structurally diverse δ-lactams that might have further applications in medicinal chemistry.

## Graphical abstract

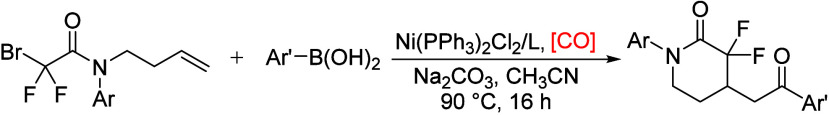

As privileged structural units in organic synthesis and medicinal chemistry, lactams are pharmaceutically important core skeletons that are present in a broad range of natural products, bioactive molecules, and pharmaceuticals.^1^ However, compared to β- and γ-lactams,^2,3^ δ-lactam-containing drug molecules are comparatively rare.^4^ From the perspective of organic and medicinal chemistry, the functionalization of organic molecules may lead to structural diversity and complexity, which has an important effect on their applications in drug design and exploration. The introduction of fluorine atoms into the molecules may be one good modification, as fluorine is the most reactive element with unique characteristics, such as a small radius, high electronegativity, and strong C–F bond strength.^5^ The presence of fluorine atoms usually has a great influence on the physical, chemical, and biological properties of molecules.^6^ In particular, the *gem*-difluoro group has gained extensive research interest due to its wide applications in agrochemical and pharmaceutical industries.^7^ In this regard, installing the *gem*-difluoro group on δ-lactams may expand the diversity of these structures, which is of great interest in drug discovery and biology research.

In addition, as key building blocks in organic chemistry, carbonyl-containing compounds have always been utilized for the construction of complex drug molecules owing to their high reactivity and versatile transformability. In this regard, the preparation of carbonyl-containing compounds has drawn continuous attention from both academic and industrial fields. In recent decades, transition-metal-catalyzed carbonylation reactions have emerged as economic and powerful tools due to their broad utility in the synthesis of carbonyl-containing compounds.^8^ Generally, commonly used transition metals in carbonylation reactions mainly focus on palladium, rhodium, and iridium due to their high efficiency and good reactivity. However, their high price combined with some even more expensive ligands restricts their usage in industry and fine chemicals. Thus, low-cost and abundant metals such as nickel provide a good option. However, the use of nickel catalysts in carbonylation reactions is relatively rare because of nickel’s high affinity for CO, which will lead to catalyst deactivation.^9^ To overcome this disadvantage, a series of nickel-catalyzed radical carbonylation reactions with low-pressure CO gas or CO surrogates have been explored.^10^ The pioneering studies from Zhang and co-workers especially have inspired further developments.^10c^ Considering the synthetic value of functionalized δ-lactams, as well as our research interest in nickel-catalyzed carbonylation reactions,^11^ herein, we wish to disclose a nickel-catalyzed radical cyclization and carbonylation reaction of bromodifluoroacetamides with arylboronic acids for the synthesis of δ-lactams bearing both *gem*-difluoro and carbonyl groups.

At the outset, 2-bromo-*N*-(but-3-en-1-yl)-2,2-difluoro-*N*-phenylacetamide **1a** and *p*-tolylboronic acid **2a** were chosen as the model substrates to screen the reaction conditions. To our delight, the desired product **3aa** was obtained in 59% yield by using Ni­(PPh_3_)_2_Cl_2_ as the catalyst, **L1** as the ligand, Na_2_CO_3_ as the base, and HCOOH as the CO source in THF at 80 °C for 16 h (Table 1, entry 1). Then, catalysts such as NiBr_2_·DME, Ni­(acac)_2_, Ni­(OTf)_2_, and Ni­(PCy_3_)_2_Cl_2_ were examined, and lower yields were detected (Table 1, entries 2–5). Next, various ligands were investigated; no further improvement was achieved (Table 1, entries 6–10). Subsequently, the effect of base was studied, and the yield of product **3aa** was decreased by using K_2_CO_3_, K_3_PO_4_, and NaHCO_3_ as the base (Table 1, entries 11–13). It was noteworthy that the yield of the expected product was observed to be 63% when increasing the temperature to 90 °C (Table 1, entry 14). Solvents including 1,4-dioxane, DME, DMA, and CH_3_CN were tested (Table 1, entries 15–18), and CH_3_CN tended to be the optimal solvent, resulting in the final product in 68% yield (Table 1, entry 18). Finally, 78% yield of **3aa** was formed by changing the amount of HCOOH to 2.5 mmol (Table 1, entry 19). Notably, the five-membered ring product could be detected in the same cases during the optimization process, but always in a small amount, and the yield could not be improved. Other compounds based on noncarbonylation and/or noncyclization could be detected as well. It is worth mentioning that no desired product could be detected when the reaction was performed under 1 bar of CO gas.

**Table 1:**
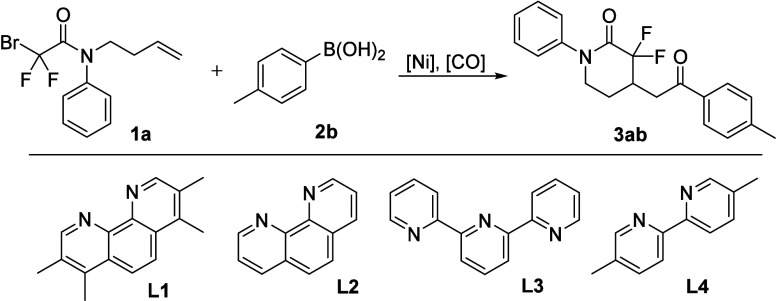
Screening of Reaction Conditions^a^

entry	catalyst	ligand	base	solvent	yield
1	Ni(PPh_3_)_2_Cl_2_	L1	Na_2_CO_3_	THF	59%
2	NiBr_2_·DME	L1	Na_2_CO_3_	THF	50%
3	Ni(acac)_2_	L1	Na_2_CO_3_	THF	44%
4	Ni(OTf)_2_	L1	Na_2_CO_3_	THF	45%
5	Ni(PCy_3_)_2_Cl_2_	L1	Na_2_CO_3_	THF	48%
6	Ni(PPh_3_)_2_Cl_2_	L2	Na_2_CO_3_	THF	42%
7	Ni(PPh_3_)_2_Cl_2_	L3	Na_2_CO_3_	THF	21%
8	Ni(PPh_3_)_2_Cl_2_	L4	Na_2_CO_3_	THF	28%
9	Ni(PPh_3_)_2_Cl_2_	dtbbpy	Na_2_CO_3_	THF	trace
10	Ni(PPh_3_)_2_Cl_2_	bpy	Na_2_CO_3_	THF	20%
11	Ni(PPh_3_)_2_Cl_2_	L1	K_2_CO_3_	THF	47%
12	Ni(PPh_3_)_2_Cl_2_	L1	K_3_PO_4_	THF	25%
13	Ni(PPh_3_)_2_Cl_2_	L1	NaHCO_3_	THF	30%
14^b^	Ni(PPh_3_)_2_Cl_2_	L1	Na_2_CO_3_	THF	63%
15^b^	Ni(PPh_3_)_2_Cl_2_	L1	Na_2_CO_3_	dioxane	52%
16^b^	Ni(PPh_3_)_2_Cl_2_	L1	Na_2_CO_3_	DME	61%
17^b^	Ni(PPh_3_)_2_Cl_2_	L1	Na_2_CO_3_	DMA	56%
18^b^	Ni(PPh_3_)_2_Cl_2_	L1	Na_2_CO_3_	CH_3_CN	68%
19^b^ ^c^	Ni(PPh_3_)_2_Cl_2_	L1	Na_2_CO_3_	CH_3_CN	78%

a Reaction conditions: **1a** (0.2 mmol), **2b** (0.3 mmol), catalyst (10 mol %), ligand (10 mol %), [CO] (HCOOH + Ac_2_O, 2 mmol), base (1.5 equiv), solvent (2 mL), 80 °C, 16 h, isolated yields. dtbbpy: 4 4-di-*tert*-butyl-2 2-bipyridine. bpy: bipyridine.

b 90 °C.

c [CO] (HCOOH + Ac_2_O, 2.5 mmol).

With the best reaction conditions in hand, the generality of this radical cyclization/carbonylation reaction was explored with a variety of arylboronic acids. As shown in Scheme 1, phenylboronic acid could work well to give product **3aa** in a good yield. Arylboronic acids with electron-rich groups, such as methyl, *tert*-butyl, methoxy and trifluoromethoxy, were compatible to furnish the desired products in good yields (**3ab**–**3ag**). Arylboronic acids with electron-withdrawing groups, including acetyl and trifluoromethyl, were tolerated well to provide the expected products in moderate yields (**3ah**–**3ai**). Halogen substituents, such fluoro, chloro, and bromo groups, could also react with *p*-tolylboronic acid smoothly, generating the corresponding products in moderate yields (**3aj**–**3al**). The 2-naphthalenyl group was then tested, and the target product was obtained in 73% yield (**3am**). Additionally, thiophen-3-ylboronic acid was applied as the substrate, and the desired product was isolated in 35% yield (**3an**). However, the reactions with alkyl boronic acids and alkenyl boronic acids under our standard conditions failed. Besides their decreased stability, the low stability of the corresponding alkyl/alkenyl nickel complex could be another reason for these failures.

**Scheme 1:**
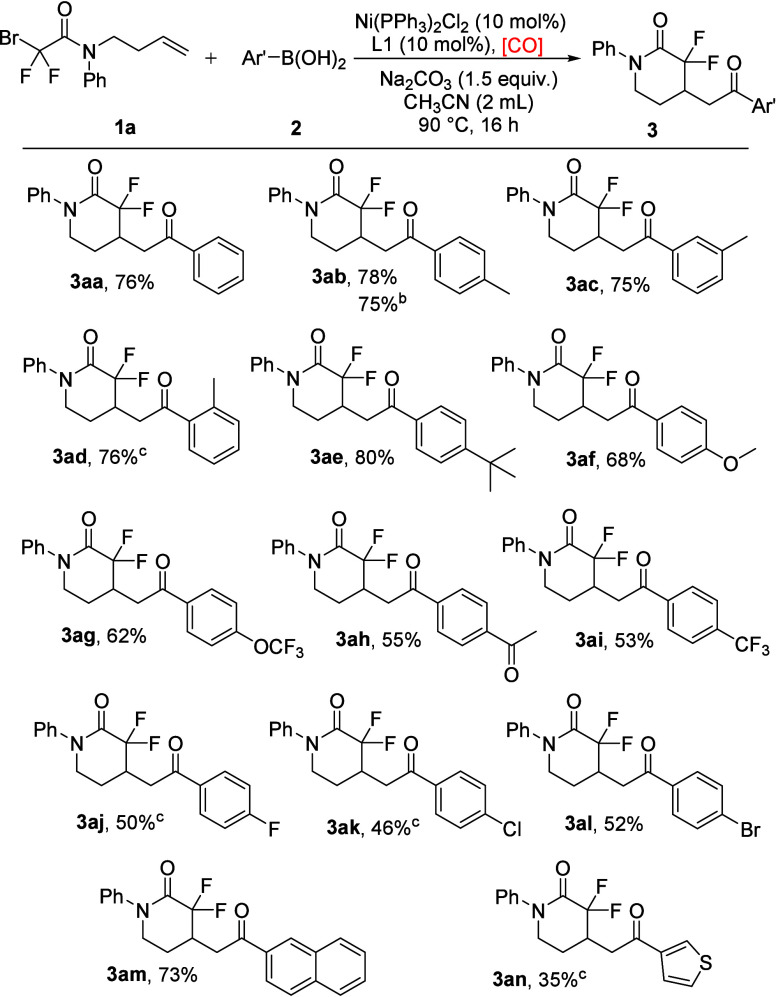
Substrate Scope of Arylboronic Acids^a^

a Reaction conditions: 2-bromo-*N*-(but-3-en-1-yl)-2,2-difluoro-*N*-phenylacetamide **1a** (0.2 mmol), arylboronic acids **2** (0.3 mmol), Ni­(PPh_3_)_2_Cl_2_ (10 mol %), L1 (10 mol %), [CO] (HCOOH + Ac_2_O, 2.5 mmol), Na_2_CO_3_ (1.5 equiv), CH_3_CN (2 mL), 90 °C, 16 h. Isolated yield.
b Performed at 1 mmol scale.
c With 95% purity.

Next, we turned our attention to the substrate scope of bromodifluoroacetamides (Scheme 2). Substrates with electron-donating groups could work smoothly to give the desired products in moderate to good yields under the standard reaction conditions (**3bb**–**3fb**). Halogen groups, including fluoro, chloro, and bromo, proved to be the suitable substrates and delivered the expected products in 60–85% yields (**3gb**–**3ib**). A biphenyl substrate was then examined, furnishing the target product in moderate yields (**3jb**). Notably, the substrate with a 3,5-dimethyl group could be efficiently converted to the final product in 65% yield (**3kb**). Furthermore, the substrate bearing the benzo­[*d*]­[1,3]­dioxol-5-yl substituent was examined, affording the corresponding product in good yield (**3 lb**). However, only a trace amount of the desired product was detected when the phenyl ring was *para*-substituted with CF_3_ or acetyl or the phenyl was replaced with pyridine.

**Scheme 2:**
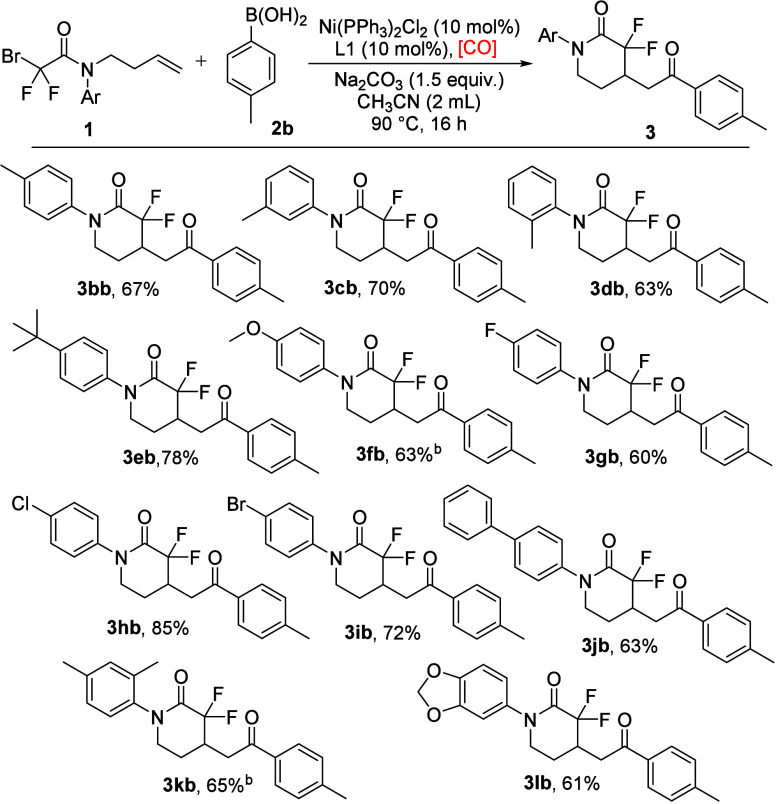
Substrate Scope of Bromodifluoroacetamides^a^

a Reaction conditions: bromodifluoroacetamides **1** (0.2 mmol), *p*-tolylboronic acid **2b** (0.3 mmol), Ni­(PPh_3_)_2_Cl_2_ (10 mol %), L1 (10 mol %), [CO] (HCOOH + Ac_2_O, 2.5 mmol), Na_2_CO_3_ (1.5 equiv), CH_3_CN (2 mL), 90 °C, 16 h. Isolated yield.
b With 95% purity.

Moreover, a control experiment was carried out to gain more insight into the reaction mechanism (Scheme 3). Under the standard reaction conditions, TEMPO was added to the reaction mixture as a radical scavenger, and product **3ab** was not observed, which indicated that a free radical process may be included in this reaction.

**Scheme 3:**
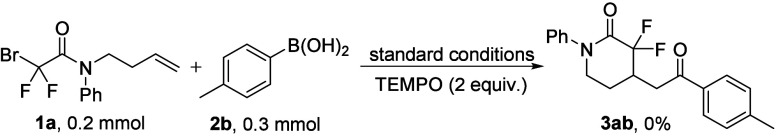
Control Experiment

Based on above outcomes and previous reports,^10c^ a possible reaction mechanism is shown in Scheme 4. First, transmetalation of arylboronic acids **2** with Ni­(II) affords the aryl Ni­(II) species **I**, which undergoes carbonylation to yield ArCONi­(II) complex **II**. Then, ArCONi­(II) complex **II** react with bromodifluoroacetamides **1** through a SET process to give radical **A** and ArCONi­(III) complex **III**. Next, a 6-*exo*-*trig* cyclization of **A** occurs to provide the radical **B**. This newly formed radical **B** reacts with another molecule of ArCONi­(II) complex II to produce ArCONi­(III) species **IV**, which undergoes reductive elimination to yield Ni­(I) and the desired product. Finally, the released Ni­(I) reacts with ArCONi­(III) via a comproportionation pathway to regenerate Ni­(II) and ArCONi­(II).

**Scheme 4:**
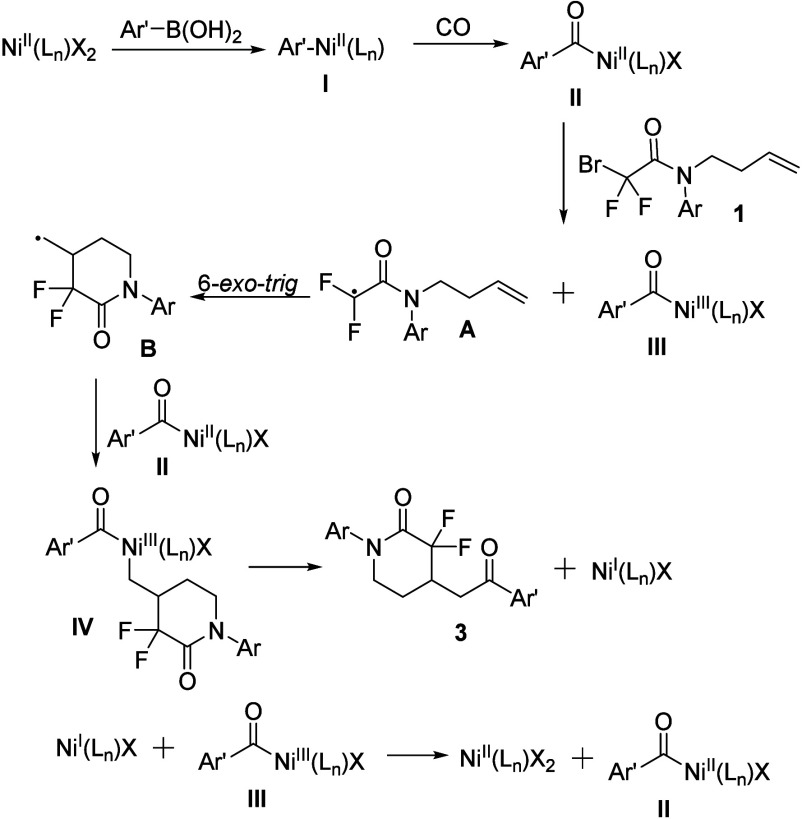
Proposed Reaction Mechanism

In summary, an efficient nickel-catalyzed radical cyclization and carbonylation reaction of bromodifluoroacetamides with arylboronic acids has been disclosed. By using formic acid as the CO source, a variety of δ-lactams bearing both *gem*-difluoro and carbonyl groups were obtained in moderate to good yields. This reaction features mild reaction conditions, good functional group tolerance, and no manipulation of toxic CO gas. It provides a good addition to access δ-lactams with diverse set of functional groups through a nickel-catalyzed carbonylation reaction.

## Supplementary Material



## Data Availability

The data underlying this study are available in the published article and its Supporting Information

## Notes

Experimental details, characterization data and NMR spectra (PDF)
