# Supplementary material for: Nickel-Catalyzed Carbonylative Cyclization of Bromodifluoroacetamides with Arylboronic Acids toward δ‑Lactams
Source: Org Lett. 2025 Aug 28;27(36):10136–40. doi: 10.1021/acs.orglett.5c03212 (PMC12442075; doi:10.1021/acs.orglett.5c03212)
Supplement: Supplementary file 1 [file ol5c03212_si_001.pdf]

Supporting Information

Nickel-Catalyzed Carbonylative Cyclization of Bromodifluoroacetamides with Arylboronic Acids Toward δ-Lactams

Hucheng Ma,[†,#] Chen-Yang Hou,[†,#] Yuting Jiang,[†] Xinxin Qi,*[,†] Xiao-Feng Wu*[,‡]
†Department of Chemistry, Key Laboratory of Surface & Interface Science of Polymer Materials of Zhejiang Province, Zhejiang Sci-Tech University, Hangzhou, Zhejiang 310018, People's Republic of China.
E-mail: xinxinqi@zstu.edu.cn
‡Dalian National Laboratory for Clean Energy, Dalian Institute of Chemical Physics,Chinese Academy of Sciences, 116023, Dalian, Liaoning, China; Leibniz-Institut für Katalyse e.V. an der, Institution Universität Rostock, Albert-Einstein-Straße 29a, Rostock 18059, Germany.
E-mail: xiao-feng.wu@catalysis.de
[#]These authors contributed equally.

Table of Contents

1. General Information 2

2. General Procedures 3

2.1 General Procedures for the Synthesis of 2-bromo-*N*-(but-3-en-1-yl)-2,2-difluoro-*N*-arylacetamides 3

2.2 General Procedure for the Synthesis of Products 4

3. Characterization Data of Products and 1i 4

4. Reference 22

5. Copy of ^{1}H and ^{13}C NMR Spectra of Products and 1i 23

1. General Information

Unless otherwise noted, all reactions were carried out under N_2 atmosphere. All chemicals and reagents were obtained from Macklin, J&K chemical, energy chemical, Bidepharm and Sigma-Aldrich, and were used without further purification. All solvents were dry solvents. Column chromatography was performed on silica gel (200-300 meshes) using petroleum ether and ethyl acetate as eluent. NMR spectra were recorded on a Bruker Avance operating at for ^1H NMR at 400 MHz, ^{13}C NMR at 101 MHz and spectral data were reported in ppm relative to tetramethylsilane (TMS) as internal standard and $CDCl_3$ (^1H NMR δ 7.26, ^{13}C NMR δ 77.16) as solvent. All coupling constants (J) are reported in Hz. The following abbreviations were used to describe peak splitting patterns when appropriate: s = singlet, d = doublet, dd = double doublet, ddd = double doublet of doublets, t = triplet, dt = double triplet, q = quartet, m = multiplet, br = broad. Gas chromatography (GC) analyses were performed on a Shimadzu GC-2014C chromatograph equipped with a FID detector. Mass spectra (MS) were measured on spectrometer by direct inlet at 70 eV. Mass spectroscopy data of the products were collected on an HRMS-TOF instrument or Waters TOFMS GCT Premier using EI or ESI ionization. Melting points were measured with WRR digital point apparatus and not corrected.

2. General Procedures

2.1 General Procedure for the Synthesis of 2-bromo-*N*-(but-3-en-1-yl)-2,2-difluoro-*N*-arylacetamides

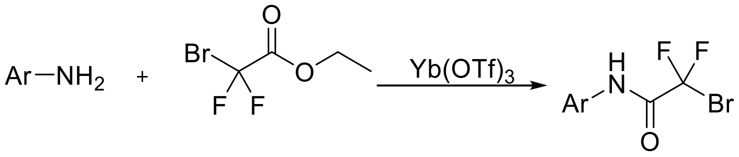

Step I: In a 25 mL of round bottom flask was charged with $Yb(OTf)_3$ (100 mg), ethyl bromodifluoroacetate (1.28 mL, 10.0 mmol,1.0 equiv.), Arylamine (0.9 mL, 10.0 mmol, 1.0 equiv.). The reaction mixture was stirred at 38 °C (oil bath) for 24 h. After completion of the reaction (TLC), the reaction was quenched with HCl (10%). The products were extracted with ethyl acetate, washed with brine. The combined organic layers were dried over Na_2SO_4. The solvent was removed under a reduced pressure and the residue was purified by silica gel column chromatography to give the 2-bromo-2,2-difluoro-*N*-arylacetamides as white solid (1.5 g, 6.0 mmol).

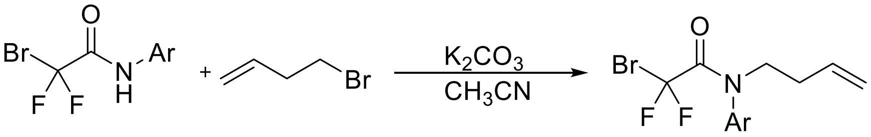

Step II: In a 50 mL of round bottom flask was charged with 2-bromo-2,2-difluoro-*N*-arylacetamides (1.5 g, 6 mmol, 1.0 equiv.), K_2CO_3 (2.5 g, 18 mmol, 3.0 equiv.) and CH_3CN (20 mL). 4-bromo-1-butene (1.8 mL, 18 mmol, 3.0 equiv.) was added to the solution and the mixture was stirred magnetically at 90 °C (oil bath) for 24 h. After the reaction was completed monitored by TLC, the solvent was removed under a reduced pressure. The reaction mixture was quenched with water, extracted with EtOAc, washed with brine, dried over anhydrous Na_2SO_4. The crude material was purified by column chromatography on silica gel to afford 2-bromo-*N*-(but-3-en-1-yl)-2,2-difluoro-*N*-arylacetamides.

2.2 General Procedure for the Synthesis of Products

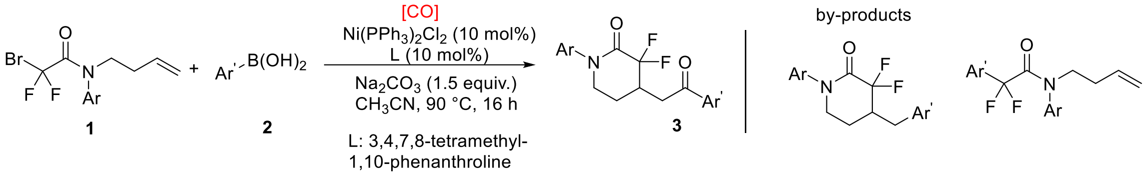

1 (0.2 mmol, 1.0 equiv.), **2** (0.3 mmol, 1.5 equiv.), $Ni(PPh_3)_2Cl_2$ (10 mol%), 3,4,7,8-tetramethyl-1,10-phenanthroline (10 mol%), and Na_2CO_3 (0.3 mmol, 1.5 equiv.) were added to an oven-dried tube (20 mL) which was then placed under vacuum and refilled with nitrogen for three times. Then dry CH_3CN (2.0 mL) was added into the tube via a syringe. A mixture of formic acid (2.5 mmol) and acetic anhydride (2.5 mmol) was stirred at 30 °C (oil bath) for 1.5 h, which was then added to a small inner tube with Et_3N (2.5 mmol). The tube was sealed and the mixture was stirred at 90 °C (oil bath) for 16 h. After the reaction was completed, the reaction mixture was filtered and concentrated under vacuum. The crude product was purified by column chromatography (petroleum ether : ethyl acetate = 20 : 1 to 5 : 1) on silica gel to afford the corresponding product **3**.

1 mmol scale: **1a** (1 mmol, 1.0 equiv.), **2b** (1.5 mmol, 1.5 equiv.), $Ni(PPh_3)_2Cl_2$ (10 mol%, 65.4 mg), 3,4,7,8-tetramethyl-1,10-phenanthroline (10 mol%, 23.6 mg), and Na_2CO_3 (1.5 mmol, 1.5 equiv.) were added to an oven-dried tube (50 mL) which was then placed under vacuum and refilled with nitrogen for three times. Then dry CH_3CN (10.0 mL) was added into the tube via a syringe. A mixture of formic acid (12.5 mmol) and acetic anhydride (12.5 mmol) was stirred at 30 °C (oil bath) for 1.5 h, which was then added to a small inner tube with Et_3N (12.5 mmol). The tube was sealed and the mixture was stirred at 90 ℃ (oil bath) for 16 h. After the reaction was completed, the reaction mixture was filtered and concentrated under vacuum. The crude product was purified by column chromatography (petroleum ether : ethyl acetate = 20 : 1 to 5 : 1) on silica gel to afford the corresponding product **3ab** in 75% yield (257.25 mg).

Failed substrates:

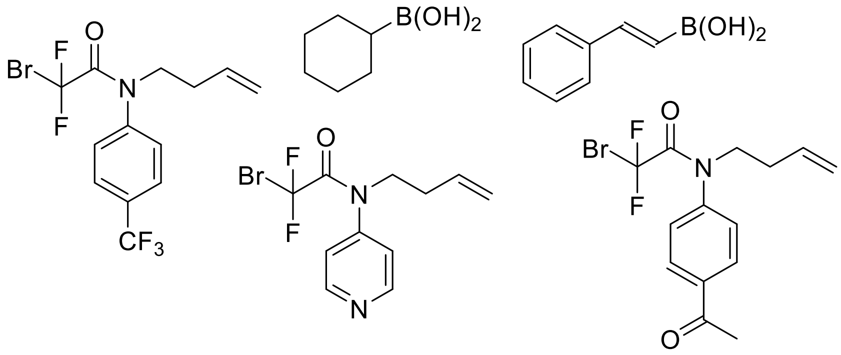

3. Characterization Data of Products and1i.

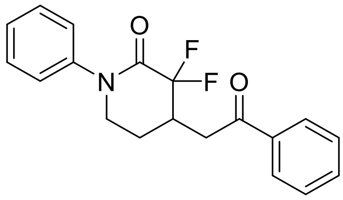

3,3-difluoro-4-(2-oxo-2-phenylethyl)-1-phenylpiperidin-2-one (3aa)

Upon completion the mixture was concentrated and purified via flash column chromatography (petroleum ether : ethyl acetate = 5 : 1, Rf = 0.4) to give the titled product **3aa** as a white solid (50.1 mg, 76%).

^{1}H NMR (400 MHz, $CDCl_3$) δ 8.01 (d, J = 7.3 Hz, 2H), 7.62 (t, J = 7.4 Hz, 1H), 7.51 (t, J = 7.6 Hz, 2H), 7.43 (t, J = 7.7 Hz, 2H), 7.31 (dd, J = 10.0, 8.8 Hz, 3H), 3.98 – 3.90 (m, 1H), 3.66 – 3.61 (m, 1H), 3.56 (d, J = 15.9 Hz, 1H), 3.22 – 3.08 (m, 2H), 2.30 – 2.25 (m, 1H), 2.08 – 1.97 (m, 1H).

^{13}C NMR (101 MHz, $CDCl_3$) δ 196.9, 161.1 (t, J = 30.1 Hz), 141.0, 136.3, 133.7, 129.4, 128.8, 128.0, 127.7, 125.6, 113.6 (dd, J = 248.9, 244.9 Hz), 50.1, 37.4 (t, J = 20.7 Hz), 35.3, 25.0 (d, J = 7.5 Hz).

^{19}F NMR (376 MHz, $CDCl_3$) δ -108.26 (dd, J = 278.6, 23.7 Hz), -110.22 (d, J = 278.5 Hz).

M.p. 64.9 – 65.3 ℃

HRMS (ESI-TOF) m/z: $[M+H]^+$ Calcd. for $C_{19}H_{18}F_2NO_2$ 330.1300; found: 330.1305.

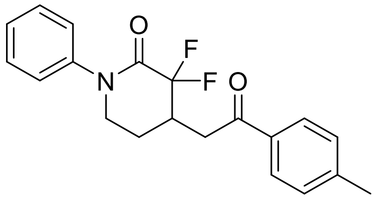

3,3-difluoro-4-(2-oxo-2-(*p*-tolyl)ethyl)-1-phenylpiperidin-2-one (3ab)

Upon completion the mixture was concentrated and purified via flash column chromatography (petroleum ether : ethyl acetate = 5 : 1, Rf = 0.4) to give the titled product **3ab** as a white solid (53.5 mg, 78%).

^{1}H NMR (400 MHz, $CDCl_3$) δ 7.91 (d, J = 8.1 Hz, 2H), 7.43 (t, J = 7.6 Hz, 2H), 7.31 (t, J = 9.5 Hz, 5H), 3.94 (t, J = 9.9 Hz, 1H), 3.63 (d, J = 10.1 Hz, 1H), 3.52 (d, J = 16.9

Hz, 1H), 3.13 (dt, *J* = 26.3, 10.2 Hz, 2H), 2.43 (s, 3H), 2.27 (d, *J* = 13.5 Hz, 1H), 2.07 – 2.00 (m, 1H).

^{13}C NMR (101 MHz, CDCl$_3$) *δ* 196.6, 161.2 (t, *J* = 30.0 Hz), 144.6, 141.0, 133.9, 129.4, 129.4, 129.1, 128.2, 127.7, 125.6, 113.6 (dd, *J* = 249.0, 244.9 Hz), 50.1, 37.4 J (t, *J* = 20.8 Hz), 35.2, 24.9 (d, *J* = 7.5 Hz). 21.7.

^{19}F NMR (376 MHz, CDCl$_3$) *δ* -108.29 (dd, *J* = 278.8, 23.8 Hz), -110.24 (d, *J* = 278.7 Hz).

M.p. 131.8 – 132.4 ℃

HRMS (ESI-TOF) m/z: $[M+H]^+$ Calcd. for $C_{20}H_{20}F_2NO_2$ 344.1457; found: 344.1466.

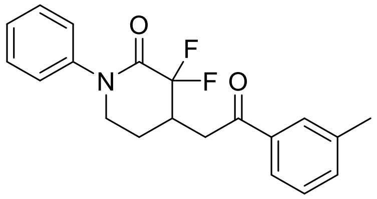

3,3-difluoro-4-(2-oxo-2-(*m*-tolyl)ethyl)-1-phenylpiperidin-2-one (3ac)

Upon completion the mixture was concentrated and purified via flash column chromatography (petroleum ether : ethyl acetate = 5 : 1, Rf = 0.4) to give the titled product **3ac** as a yellow solid (51.2 mg, 75%).

^{1}H NMR (400 MHz, CDCl$_3$) *δ* 7.81 (d, *J* = 6.7 Hz, 2H), 7.45 – 7.37 (m, 4H), 7.34 – 7.29 (m, 3H), 3.97 – 3.90 (m, 1H), 3.66 – 3.52 (m, 1H), 3.54 (d, *J* = 15.8 Hz, 1H), 3.21 – 3.07 (m, 2H), 2.44 (s, 3H), 2.30 – 2.22 (m, 1H), 2.07 – 1.96 (m, 1H).

^{13}C NMR (101 MHz, CDCl$_3$) *δ* 197.1, 161.1 (t, *J* = 30.2 Hz), 141.1, 138.6, 136.4, 134.4, 129.4, 128.7, 128.6, 127.7, 127.6, 125.6, 125.3, 113.6 (dd, *J* = 248.9, 244.9 Hz), 50.1, 37.4 (t, *J* = 20.7 Hz), 35.4, 24.9 (d, *J* = 7.5 Hz), 21.3.

^{19}F NMR (376 MHz, CDCl$_3$) *δ* -108.29 (dd, *J* = 278.6, 23.7 Hz), -110.24 (d, *J* = 278.4 Hz).

M.p. 65.0 – 65.7 ℃

HRMS (ESI-TOF) m/z: $[M+H]^+$ Calcd. for $C_{20}H_{20}F_2NO_2$ 344.1457; found: 344.1466.

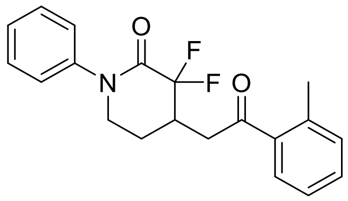

3,3-difluoro-4-(2-oxo-2-(*o*-tolyl)ethyl)-1-phenylpiperidin-2-one (3ad)

Upon completion the mixture was concentrated and purified via flash column chromatography (petroleum ether : ethyl acetate = 10 : 1, Rf = 0.4) to give the titled product **3ad** as a yellow oil (52.2mg, 76%) with 95% purity.

^{1}H NMR (400 MHz, CDCl$_3$) δ 7.75 (d, J = 7.6 Hz, 1H), 7.43 (dd, J = 12.7, 4.9 Hz, 3H), 7.34 – 7.28 (m, 5H), 3.98 – 3.92 (m, 1H), 3.67 – 3.62 (m, 1H), 3.51 (d, J = 15.4 Hz, 1H), 3.14 – 3.07 (m, 1H), 2.65 (s, 1H), 2.54 (s, 3H), 2.29 – 2.24 (m, 1H), 2.08 – 1.99 (m, 1H).

^{13}C NMR (101 MHz, CDCl$_3$) δ 200.5, 161.2 (t, J = 29.9 Hz), 141.1, 138.6, 136.8, 132.3, 132.0, 129.4, 128.8, 127.7, 126.0, 125.6, 113.6 (dd, J = 249.1, 245.1 Hz), 50.1, 37.9, 37.6 (t, J = 20.7 Hz), 25.0 (d, J = 7.6 Hz), 21.6.

^{19}F NMR (376 MHz, CDCl$_3$) δ -105.68 – -109.18 (m), -110.70 (d, J = 279.0 Hz).

HRMS (ESI-TOF) m/z: $[M+H]^+$ Calcd. for $C_{20}H_{20}F_2NO_2$ 344.1457; found: 344.1466.

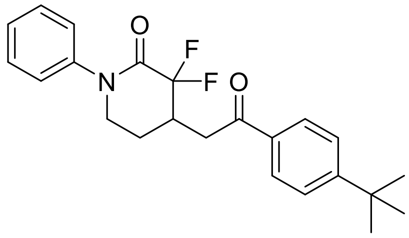

4-(2-(4-(*tert*-butyl)phenyl)-2-oxoethyl)-3,3-difluoro-1-phenylpiperidin-2-one (3ae)

Upon completion the mixture was concentrated and purified via flash column chromatography (petroleum ether : ethyl acetate = 5 : 1, Rf = 0.4) to give the titled product **3ae** as a yellow solid (61.6 mg, 80%).

^{1}H NMR (400 MHz, CDCl$_3$) δ 7.96 (d, J = 8.5 Hz, 2H), 7.52 (d, J = 8.5 Hz, 2H), 7.43 (t, J = 7.7 Hz, 2H), 7.34 – 7.29 (m, 3H), 3.97 – 3.91 (m, 1H), 3.66 – 3.61 (m, 1H), 3.54 (d, J = 15.7 Hz, 1H), 3.21 – 3.14 (m, 2H), 2.29 – 2.24 (m, 1H), 2.07 – 1.98 (m, 1H), 1.36 (s, 9H).

^{13}C NMR (101 MHz, CDCl$_3$) δ 196.6, 170.9, 161.2 (t, J = 30.1 Hz), 157.5, 157.3, 141.0, 133.8, 130.0, 129.4, 128.0, 127.7, 126.5, 125.7, 125.6, 125.4, 113.6 (dd, J = 248.8, 245.0 Hz), 50.1, 37.4 (t, J = 20.7 Hz), 35.2, 35.2, 31.1, 31.0, 25.0 (d, J = 7.4 Hz).

^{19}F NMR (376 MHz, CDCl$_3$) δ -108.86 (dd, J = 278.9, 23.8 Hz), -110.74 (d, J = 278.8 Hz).

M.p. 139.6 – 140.0 °C

HRMS (ESI-TOF) m/z: [M+H]$^+$ Calcd. for $C_{23}H_{26}F_2NO_2$ 386.1926; found: 386.1929.

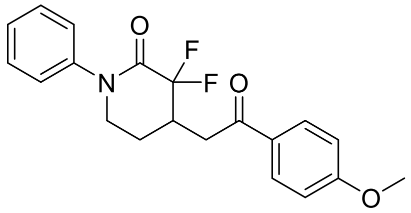

3,3-difluoro-4-(2-(4-methoxyphenyl)-2-oxoethyl)-1-phenylpiperidin-2-one (3af)

Upon completion the mixture was concentrated and purified via flash column chromatography (petroleum ether : ethyl acetate = 5 : 1, Rf = 0.4) to give the titled product **3af** as a white solid (48.9 mg, 68%).

^{1}H NMR (400 MHz, CDCl$_3$) δ 7.99 (d, J = 8.9 Hz, 2H), 7.43 (t, J = 7.7 Hz, 2H), 7.34 – 7.29 (m, 3H), 6.97 (d, J = 8.9 Hz, 2H), 3.96 – 3.92 (m, 1H), 3.89 (s, 3H), 3.65 – 3.60 (m, 1H), 3.50 (d, J = 15.5 Hz, 1H), 3.17 – 3.05 (m, 2H), 2.26 (dd, J = 10.0, 6.9 Hz, 1H), 2.07 – 1.96 (m, 1H).

^{13}C NMR (101 MHz, CDCl$_3$) δ 195.4, 163.9, 161.2 (t, J = 30.2 Hz).141.1, 130.4, 129.5, 129.4, 127.7, 125.6, 113.8 (dd, J = 275.0, 218.6 Hz), 55.5, 50.2, 37.5 (t, J = 20.6 Hz), 34.9, 25.0 (d, J = 7.5 Hz).

^{19}F NMR (376 MHz, CDCl$_3$) δ -108.82 (dd, J = 278.4, 24.0 Hz), -110.78 (d, J = 278.3 Hz).

M.p. 136.0 – 136.8 °C

HRMS (ESI-TOF) m/z: [M+H]$^+$ Calcd. for $C_{20}H_{20}F_2NO_3$ 360.1406; found: 360.1413.

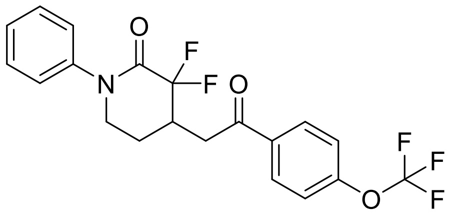

3,3-difluoro-4-(2-oxo-2-(4-(trifluoromethoxy)phenyl)ethyl)-1-phenylpiperidin-2-one (3ag)

Upon completion the mixture was concentrated and purified via flash column chromatography (petroleum ether : ethyl acetate = 5 : 1, Rf = 0.5) to give the titled product **3ag** as a yellow solid (51.25 mg, 62%).

^{1}H NMR (400 MHz, CDCl$_3$) δ 8.07 (d, *J* = 8.8 Hz, 2H), 7.44 (t, *J* = 7.7 Hz, 2H), 7.34 – 7.29 (m, 5H), 3.98 – 3.91 (m, 1H), 3.67 – 3.62 (m, 1H), 3.55 (d, *J* = 15.8 Hz, 1H), 3.20 – 3.07 (m, 2H), 2.30 – 2.25 (m, 1H), 2.09 – 1.98 (m, 1H).

^{13}C NMR (101 MHz, CDCl$_3$) δ 195.4, 161.0 (t, *J* = 29.8 Hz), 153.0, 141.0, 133.3 (d, *J* = 240.9 Hz), 130.2, 129.4, 127.8, 125.6, 117.5 (dd, *J* = 554.9, 254.1 Hz), 113.5, 50.1, 37.4 (t, *J* = 20.7 Hz), 35.5, 35.5, 25.0 (d, *J* = 7.5 Hz).

^{19}F NMR (376 MHz, CDCl$_3$) δ -57.57, -108.20 (dd, *J* = 278.9, 24.0 Hz), -110.24 (d, *J* = 279.0 Hz).

M.p. 135.0 – 135.6 °C

HRMS (ESI-TOF) m/z: $[M+H]^+$ Calcd. for $C_{20}H_{17}F_5NO_3$ 414.1123; found: 414.1130.

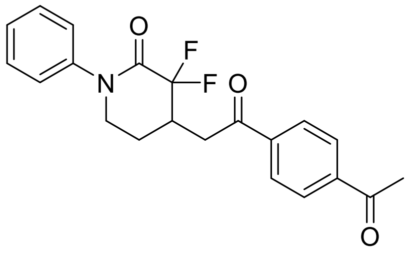

4-(2-(4-acetylphenyl)-2-oxoethyl)-3,3-difluoro-1-phenylpiperidin-2-one (3ah)

Upon completion the mixture was concentrated and purified via flash column chromatography (petroleum ether : ethyl acetate = 5 : 1, Rf = 0.4) to give the titled product **3ah** as a yellow solid (46.1mg, 55%).

^{1}H NMR (400 MHz, CDCl$_3$) *δ* 8.10 – 8.05 (m, 4H), 7.43 (t, *J* = 7.7 Hz, 2H), 7.34 – 7.29 (m, 3H), 3.99 – 3.92 (m, 1H), 3.67 – 3.57 (m, 1H), 3.59 (d, *J* = 16.0 Hz, 1H), 3.24 – 3.08 (m, 2H), 2.66 (s, 3H), 2.30 – 2.22 (m, 1H), 2.09 – 1.99 (m, 1H).

^{13}C NMR (101 MHz, CDCl$_3$) *δ* 197.3, 196.4, 161.0 (t, *J* = 30.0 Hz), 141.0, 140.6, 139.3, 129.4, 128.6, 128.3, 127.7, 125.6, 113.5 (dd, *J* = 249.2, 245.1 Hz), 50.1, 37.4 (t, *J* = 20.7 Hz), 35.8, 26.9, 25.0 (d, *J* = 7.5 Hz).

^{19}F NMR (376 MHz, CDCl$_3$) *δ* -108.14 (dd, *J* = 278.8, 23.7 Hz), -110.16 (d, *J* = 278.7 Hz).

M.p. 151.6 – 152.1 °C

HRMS (ESI-TOF) m/z: $[M+H]^+$ Calcd. for $C_{21}H_{20}F_2NO_3$ 372.1406; found: 372.1410.

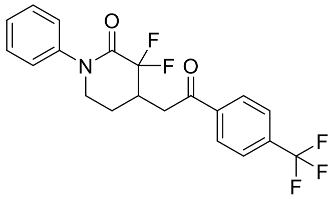

3,3-difluoro-4-(2-oxo-2-(4-(trifluoromethyl)phenyl)ethyl)-1-phenylpiperidin-2-one (3ai)

Upon completion the mixture was concentrated and purified via flash column chromatography (petroleum ether : ethyl acetate = 5 : 1, Rf = 0.4) to give the titled product **3ai** as a white solid (42.1 mg, 53%).

^{1}H NMR (400 MHz, CDCl$_3$) *δ* 8.12 (d, *J* = 8.1 Hz, 2H), 7.77 (d, *J* = 8.3 Hz, 2H), 7.44 (t, *J* = 7.7 Hz, 2H), 7.35 – 7.29 (m, 3H), 4.00 – 3.92 (m, 1H), 3.68 – 3.63 (m, 1H), 3.59 (d, *J* = 16.0 Hz, 1H), 3.23 – 3.09 (m, 2H), 2.30 – 2.24 (m, 1H), 2.10 – 1.99 (m, 1H).

^{13}C NMR (101 MHz, CDCl$_3$) *δ* 196.1, 161.0 (t, *J* = 29.7 Hz), 141.0, 138.9, 134.9 (d, *J* = 32.6 Hz), 129.4, 127.0 (d, *J* = 286.1 Hz), 125.9, 125.9, 125.6, 124.8, 122.1, 113.5 (dd, *J* = 249.2, 245.0 Hz), 50.1, 37.4 (t, *J* = 20.7 Hz), 35.8 (d, *J* = 3.0 Hz), 25.0 (d, *J* = 7.5 Hz).

^{19}F NMR (376 MHz, CDCl$_3$) *δ* -63.15 (s), -108.15 (dd, *J* = 278.8, 23.9 Hz), -110.19 (d, *J* = 279.1 Hz).

M.p. 138.8 – 139.4 °C

HRMS (ESI-TOF) m/z: $[M+H]^+$ Calcd. for $C_{20}H_{17}F_5NO_2$ 398.1174; found: 398.1183.

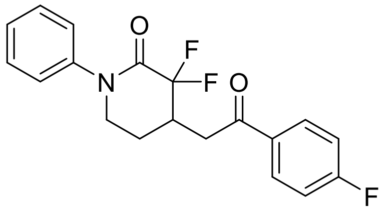

3,3-difluoro-4-(2-(4-fluorophenyl)-2-oxoethyl)-1-phenylpiperidin-2-one (3aj)

Upon completion the mixture was concentrated and purified via flash column chromatography (petroleum ether : ethyl acetate = 5 : 1, Rf = 0.4) to give the titled product **3aj** as a yellow oil (34.8 mg, around 50% yield) with 95% purity.

^{1}H NMR (400 MHz, CDCl$_3$) δ 8.05 (dd, J = 8.8, 5.4 Hz, 2H), 7.43 (q, J = 7.2 Hz, 2H), 7.32 (dd, J = 14.0, 7.5 Hz, 3H), 7.18 (t, J = 8.6 Hz, 2H), 3.99 – 3.91 (m, 1H), 3.67 – 3.61 (m, 1H), 3.53 (d, J = 15.8 Hz, 1H), 3.19 – 3.06 (m, 2H), 2.30 – 2.25 (m, 1H), 2.05 – 1.98 (m, 1H).

^{13}C NMR (101 MHz, CDCl$_3$) δ 195.3, 167.3, 164.8, 160.9 (d, J = 30.1 Hz), 141.0, 132.8, 130.8, 130.7, 129.4, 129.4, 127.7, 125.6, 116.1, 115.8, 111.2 (dd, J = 214.6, 166.8 Hz), 50.1, 37.4 (t, J = 20.8 Hz), 35.3, 25.0 (d, J = 7.6 Hz).

^{19}F NMR (376 MHz, CDCl$_3$) δ -107.89 (d, J = 24.3 Hz), -108.45 – -109.19 (m), -109.90 – -113.78 (m).

HRMS (ESI-TOF) m/z: $[M+H]^+$ Calcd. for $C_{19}H_{17}F_3NO_2$ 348.1206; found: 348.1209.

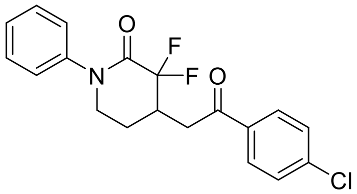

4-(2-(4-chlorophenyl)-2-oxoethyl)-3,3-difluoro-1-phenylpiperidin-2-one (3ak)

Upon completion the mixture was concentrated and purified via flash column chromatography (petroleum ether : ethyl acetate = 10 : 1, Rf = 0.4) to give the titled product **3ak** as a yellow solid (33.4 mg, around 46% yield) with 95% purity.

^{1}H NMR (400 MHz, CDCl$_3$) δ 7.95 (d, J = 8.4 Hz, 2H), 7.48 (d, J = 8.3 Hz, 2H), 7.43 (t, J = 7.6 Hz, 2H), 7.32 (dd, J = 14.0, 7.6 Hz, 3H), 3.98 – 3.91 (m, 1H), 3.66 – 3.63 (m, 1H), 3.53 (d, J = 16.1 Hz, 1H), 3.18 – 3.06 (m, 2H), 2.27 (d, J = 13.9 Hz, 1H), 2.07 – 1.98 (m, 1H).

^{13}C NMR (101 MHz, CDCl$_3$) δ 195.7, 161.0 (t, J = 30.0 Hz), 141.0, 140.2, 134.7, 129.5, 129.4, 129.1, 127.7, 125.6, 113.5 (dd, J = 249.3, 245.0 Hz), 50.1, 37.4 (t, J = 20.7 Hz), 35.4, 25.0 (d, J = 7.5 Hz).

^{19}F NMR (376 MHz, CDCl$_3$) δ -108.25 (dd, J = 278.9, 24.0 Hz), -110.26 (d, J = 278.9 Hz).

M.p. 125.8 – 126.4 ℃

HRMS (ESI-TOF) m/z: $[M+H]^+$ Calcd. for $C_{19}H_{17}ClF_2NO_2$ 364.0910; found: 364.0918.

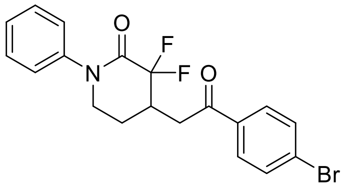

4-(2-(4-bromophenyl)-2-oxoethyl)-3,3-difluoro-1-phenylpiperidin-2-one (3al)

Upon completion the mixture was concentrated and purified via flash column chromatography (petroleum ether : ethyl acetate = 5 : 1, Rf = 0.4) to give the titled product **3al** as a yellow solid (42.5 mg, 52%).

^{1}H NMR (400 MHz, CDCl$_3$) δ 7.88 (d, J = 8.5 Hz, 2H), 7.65 (d, J = 8.5 Hz, 2H), 7.44 (t, J = 7.7 Hz, 2H), 7.32 (dd, J = 15.2, 7.4 Hz, 3H), 3.99 – 3.91 (m, 1H), 3.67 – 3.62 (m, 1H), 3.53 (d, J = 15.9 Hz, 1H), 3.18 – 3.06 (m, 2H), 2.31 – 2.24 (m, 1H), 2.08 – 1.97 (m, 1H).

^{13}C NMR (101 MHz, CDCl$_3$) δ 196.0, 163.7, 161.2 (d, J = 30.1 Hz), 141.0, 135.0, 132.1, 129.6, 129.4, 129.0, 127.8, 125.6, 107.9 (dd, J = 281.8, 277.3 Hz), 50.1, 37.4 (t, J = 21.0 Hz), 35.4, 25.1 (d, J = 7.8 Hz).

^{19}F NMR (376 MHz, CDCl$_3$) δ -108.19 (dd, J = 278.9, 24.3 Hz), -110.29 (d, J = 278.7 Hz).

M.p. 129.8 – 130.5 ℃

HRMS (ESI-TOF) m/z: $[M+H]^+$ Calcd. for $C_{19}H_{17}ClF_2NO_2$ 408.0405; found: 408.0411.

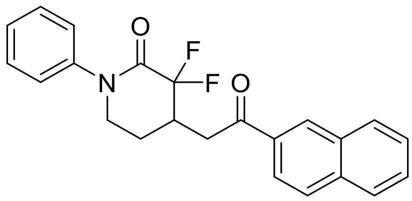

3,3-difluoro-4-(2-(naphthalen-2-yl)-2-oxoethyl)-1-phenylpiperidin-2-one (3am)

Upon completion the mixture was concentrated and purified via flash column chromatography (petroleum ether : ethyl acetate = 5 : 1, Rf = 0.4) to give the titled product **3am** as a white solid (55.4 mg, 73%).

^{1}H NMR (400 MHz, CDCl$_3$) δ 8.54 (s, 1H), 8.06 (dd, *J* = 8.6, 1.7 Hz, 1H), 8.00 (d, *J* = 8.0 Hz, 1H), 7.91 (dd, *J* = 12.2, 8.4 Hz, 2H), 7.66 – 7.57 (m, 2H), 7.44 (t, *J* = 7.7 Hz, 2H), 7.32 (dd, *J* = 8.6, 7.3 Hz, 3H), 4.00 – 3.92 (m, 1H), 3.72 – 3.63 (m, 2H), 3.33 (dd, *J* = 17.8, 10.0 Hz, 1H), 3.25 – 3.11 (m, 1H), 2.34 – 2.29 (m, 1H), 2.12 – 2.01 (m, 1H).

^{13}C NMR (101 MHz, CDCl$_3$) δ 196.8, 161.1 (t, *J* = 30.1 Hz), 141.0, 135.8, 133.6, 132.4, 130.0, 129.6, 129.4, 128.8, 128.7, 127.8, 127.7, 127.0, 125.6, 123.5, 113.7 (dd, *J* = 249.0, 245.1 Hz), 50.1, 37.5 (t, *J* = 20.7 Hz), 35.4, 25.0 (d, *J* = 7.6 Hz).

^{19}F NMR (376 MHz, CDCl$_3$) δ -108.15 (dd, *J* = 278.7, 18.6 Hz), -110.13 (d, *J* = 278.7 Hz).

M.p. 135.1 – 135.6 °C

HRMS (ESI-TOF) m/z: $[M+H]^+$ Calcd. for $C_{23}H_{20}F_2NO_2$ 380.1457; found: 380.1562.

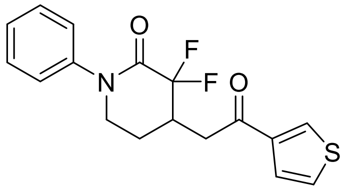

3,3-difluoro-4-(2-oxo-2-(thiophen-2-yl)ethyl)-1-phenylpiperidin-2-one (3an)

Upon completion the mixture was concentrated and purified via flash column chromatography (petroleum ether : ethyl acetate = 5 : 1, Rf = 0.4) to give the titled product **3an** as a white solid (24.1 mg, 35%) with 95% purity.

^{1}H NMR (400 MHz, CDCl$_3$) δ 8.15 (d, 1H), 7.59 (d, *J* = 5.1 Hz, 1H), 7.43 (t, *J* = 7.7 Hz, 2H), 7.37 (dd, *J* = 5.1, 2.9 Hz, 1H), 7.31 (dd, *J* = 14.5, 7.4 Hz, 3H), 3.96 – 3.89 (m,

1H), 3.66 – 3.62 (m, 1H), 3.48 (d, *J* = 15.3 Hz, 1H), 3.12 – 3.05 (m, 2H), 2.30 – 2.25 (m, 1H), 2.09 – 1.98 (m, 1H).

^{13}C NMR (101 MHz, $CDCl_3$) *δ* 191.2, 161.2 (d, *J* = 29.2 Hz), 141.7, 141.0, 132.6, 129.4, 127.7, 126.8, 126.7, 125.6, 112.3 (dd, *J* = 244.1, 5.0 Hz), 50.1, 37.4 (t, *J* = 20.8 Hz), 36.5, 25.0 (d, *J* = 7.3 Hz).

^{19}F NMR (376 MHz, $CDCl_3$) *δ* -108.48 (dd, *J* = 279.2, 23.7 Hz), -110.25 (d, *J* = 279.1 Hz).

M.p. 87.7 – 88.4 °C

HRMS (ESI-TOF) m/z: $[M+H]^+$ Calcd. for $C_{17}H_{16}F_2NO_2S$ 336.0864; found: 336.0870.

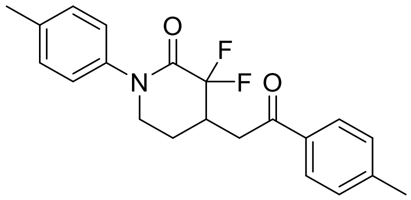

3,3-difluoro-4-(2-oxo-2-(*p*-tolyl)ethyl)-1-(*p*-tolyl)piperidin-2-one (3bb)

Upon completion the mixture was concentrated and purified via flash column chromatography (petroleum ether : ethyl acetate = 5 : 1, Rf = 0.4) to give the titled product **3bb** as a yellow oil (47.9 mg, 67%).

^{1}H NMR (400 MHz, $CDCl_3$) *δ* 7.91 (d, *J* = 8.2 Hz, 2H), 7.30 (d, *J* = 8.0 Hz, 2H), 7.23 (d, *J* = 8.2 Hz, 2H), 7.17 (d, *J* = 8.5 Hz, 2H), 3.94 – 3.86 (m, 1H), 3.63 – 3.58 (m, 1H), 3.52 (d, *J* = 15.8 Hz, 1H), 3.19 – 3.05 (m, 1H), 2.43 (s, 3H), 2.36 (s, 3H), 2.27 – 2.20 (m, 1H), 2.06 – 1.95 (m, 1H).

^{13}C NMR (101 MHz, $CDCl_3$) *δ* 196.6, 161.2 (t, *J* = 30.0 Hz), 144.6, 138.5, 137.6, 133.9, 130.0, 129.4, 128.2, 125.4, 113.6 (dd, *J* = 248.5, 245.0 Hz), 50.2, 37.4 (t, *J* = 20.7 Hz), 35.2, 24.9 (d, *J* = 7.6 Hz), 21.7, 21.0.

^{19}F NMR (376 MHz, $CDCl_3$) *δ* -108.40 (dd, *J* = 278.6, 23.4 Hz), -110.21 (d, *J* = 278.7 Hz).

HRMS (ESI-TOF) m/z: $[M+H]^+$ Calcd. for $C_{21}H_{22}F_2NO_2$ 358.1613; found: 358.1621.

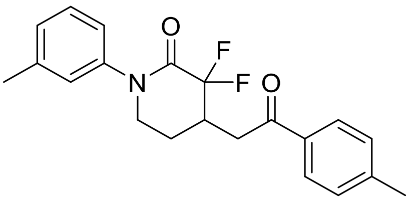

3,3-difluoro-4-(2-oxo-2-phenylethyl)-1-(*m*-tolyl)piperidin-2-one (3cb)

Upon completion the mixture was concentrated and purified via flash column chromatography (petroleum ether : ethyl acetate = 5 : 1, Rf = 0.4) to give the titled product **3cb** as a yellow oil (50.1 mg, 70%).

^{1}H NMR (400 MHz, CDCl$_3$) δ 7.91 (d, J = 8.1 Hz, 2H), 7.31 (t, J = 8.6 Hz, 3H), 7.11 (dd, J = 18.9, 9.9 Hz, 3H), 3.95 – 3.87 (m, 1H), 3.63 – 3.59 (m, 1H), 3.52 (d, J = 16.0 Hz, 1H), 3.19 – 3.05 (m, 2H), 2.44 (s, 3H), 2.37 (s, 3H), 2.26 (d, J = 13.8 Hz, 1H), 2.06 – 1.95 (m, 1H).

^{13}C NMR (101 MHz, CDCl$_3$) δ 196.6, 161.2 (t, J = 30.1 Hz), 144.6, 141.0, 139.5, 133.9, 129.4, 129.2, 128.6, 128.2, 126.4, 122.6, 113.6 (dd, J = 248.8, 245.3 Hz), 50.3, 37.5 (t, J = 20.7 Hz), 35.2, 25.0 (d, J = 7.4 Hz). 21.7, 21.3.

^{19}F NMR (376 MHz, CDCl$_3$) δ -108.40 (dd, J = 278.7, 23.6 Hz), -110.24 (d, J = 278.4 Hz).

HRMS (ESI-TOF) m/z: $[M+H]^+$ Calcd. for $C_{21}H_{22}F_2NO_2$ 358.1613; found: 358.1621.

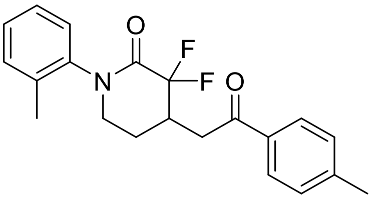

3,3-difluoro-4-(2-oxo-2-(*p*-tolyl)ethyl)-1-(*o*-tolyl)piperidin-2-one (3db)

Upon completion the mixture was concentrated and purified via flash column chromatography (petroleum ether : ethyl acetate = 5 : 1, Rf = 0.4) to give the titled product **3db** as a yellow oil (45.0 mg, 63%).

^{1}H NMR (400 MHz, CDCl$_3$) δ 7.92 (d, J = 8.2 Hz, 2H), 7.31 – 7.27 (m, 5H), 7.15 (dd, J = 8.9, 5.5 Hz, 1H), 3.93 – 3.70 (m, 1H), 3.61 – 3.38 (m, 2H), 3.22 – 3.06 (m, 2H), 2.44 (s, 3H), 2.30 – 2.26 (m, 1H), 2.23 (d, J = 5.4 Hz, 3H), 2.11 – 2.00 (m, 1H).

^{13}C NMR (101 MHz, CDCl$_3$) δ 196.6, 160.4 (t, J = 27.4 Hz), 144.6, 139.7, 134.9, 134.8, 133.9, 131.3, 129.5, 128.6, 128.5, 128.2, 127.5, 127.2, 126.9, 126.0, 114.9 (dd,

J = 243.1, 6.5 Hz), 50.0, 49.9, 37.6 (t, J = 22.9 Hz), 35.2, 35.1, 25.3 (d, J = 7.7 Hz), 21.7, 17.4, 17.1.

^{19}F NMR (376 MHz, $CDCl_3$) δ -107.79 – -109.56 (m), -111.47 (d, J = 278.8 Hz).

HRMS (ESI-TOF) m/z: $[M+H]^+$ Calcd. for $C_{21}H_{22}F_2NO_2$ 358.1613; found: 358.1621.

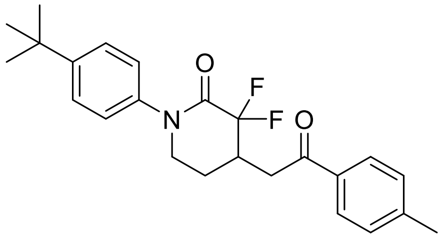

1-(4-(*tert*-butyl)phenyl)-3,3-difluoro-4-(2-oxo-2-phenylethyl)piperidin-2-one (3eb)

Upon completion the mixture was concentrated and purified via flash column chromatography (petroleum ether : ethyl acetate = 5 : 1, Rf = 0.4) to give the titled product **3eb** as a yellow solid (62.2 mg, 78%).

^{1}H NMR (400 MHz, $CDCl_3$) δ 7.91 (d, J = 8.2 Hz, 2H), 7.44 (d, J = 8.6 Hz, 2H), 7.30 (d, J = 8.0 Hz, 2H), 7.22 (d, J = 8.6 Hz, 2H), 3.96 – 3.88 (m, 1H), 3.65 – 3.60 (m, 1H), 3.52 (d, J = 15.8 Hz, 1H), 3.19 – 3.05 (m, 1H), 2.43 (s, 3H), 2.28 – 2.23 (m, 1H), 2.06 – 1.95 (m, 1H), 1.32 (s, 9H).

^{13}C NMR (101 MHz, $CDCl_3$) δ 196.6, 161.2 (t, J = 30.0 Hz), 150.6, 144.6, 138.4, 133.9, 129.5, 128.2, 126.3, 125.0, 113.7 (dd, J = 248.7, 244.8 Hz), 50.2, 37.5 (t, J = 20.6 Hz), 35.2, 34.6, 31.3, 25.0, (d, J = 7.5 Hz), 21.7.

^{19}F NMR (376 MHz, $CDCl_3$) δ -108.28 (dd, J = 278.6, 23.6 Hz), -110.11 (d, J = 278.9 Hz).

M.p. 151.4– 151.8 °C

HRMS (ESI-TOF) m/z: $[M+H]^+$ Calcd. for $C_{24}H_{28}F_2NO_2$ 400.2083; found: 400.2088.

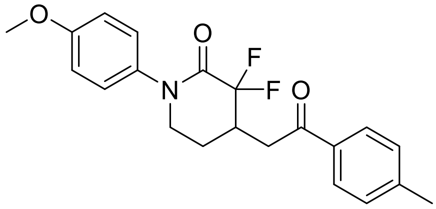

3,3-difluoro-1-(4-methoxyphenyl)-4-(2-oxo-2-(*p*-tolyl)ethyl)piperidin-2-one (3fb)

Upon completion the mixture was concentrated and purified via flash column

chromatography (petroleum ether : ethyl acetate = 5 : 1, Rf = 0.4) to give the titled product **3fb** as a white solid (47.1 mg, 63%) with 95% purity.

^{1}H NMR (400 MHz, CDCl$_3$) δ 8.13 (d, J = 7.8 Hz, 1H), 7.91 (d, J = 8.2 Hz, 1H), 7.31 (dd, J = 7.6, 5.4 Hz, 3H), 7.21 (d, J = 9.0 Hz, 2H), 6.94 (d, J = 8.9 Hz, 1H), 3.81 (s, 3H), 3.62 – 3.50 (m, 1H), 3.19 – 3.12 (m, 1H), 2.44 (s, 3H), 2.44 (s, 2H), 2.38 (s, 1H), 2.28 – 2.22 (m, 1H), 2.06 – 1.98 (m, 1H).

^{13}C NMR (101 MHz, CDCl$_3$) δ 196.6, 161.4 (t, J = 29.9 Hz), 158.8, 144.6, 142.9, 141.1, 135.7, 133.9, 133.8, 133.7, 129.5, 128.7, 128.7, 128.2, 126.8, 111.5 (dd, J = 218.5, 192.9 Hz), 109.6, 55.5, 50.5, 37.4 (t, J = 20.7 Hz), 35.2, 24.9 (d, J = 7.4 Hz), 21.9, 21.7.

^{19}F NMR (376 MHz, CDCl$_3$) δ -108.36 (dd, J = 278.8, 23.0 Hz), -110.19 (d, J = 279.2 Hz).

M.p. 133.6– 134.2 °C

HRMS (ESI-TOF) m/z: $[M+H]^+$ Calcd. for $C_{21}H_{22}F_2NO_3$ 374.1562; found: 374.1566.

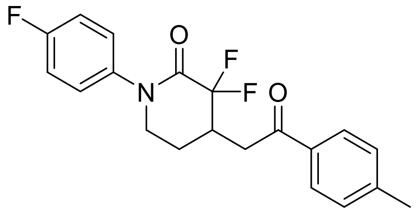

3,3-difluoro-1-(4-fluorophenyl)-4-(2-oxo-2-(*p*-tolyl)ethyl)piperidin-2-one (3gb)

Upon completion the mixture was concentrated and purified via flash column chromatography (petroleum ether : ethyl acetate = 5 : 1, Rf = 0.4) to give the titled product **3gb** as a yellow solid (43.4 mg, 60%).

^{1}H NMR (400 MHz, CDCl$_3$) δ 7.91 (d, J = 8.2 Hz, 2H), 7.41 – 7.24 (m, 4H), 7.11 (t, J = 8.5 Hz, 4H), 3.95 – 3.87 (m, 1H), 3.63 – 3.58 (m, 1H), 3.52 (d, J = 15.8 Hz, 1H), 3.19 – 3.06 (m, 2H), 2.43 (s, 3H), 2.29 – 2.25 (m, 1H), 2.07 – 1.96 (m, 1H).

^{13}C NMR (101 MHz, CDCl$_3$) δ 196.5, 161.5 (d, J = 247.6 Hz), 161.3 (t, J = 30.2 Hz), 144.6, 136.9, 133.9, 129.4, 128.2, 127.5, 127.5, 116.4, 116.2, 113.57 (dd, J = 248.9, 245.0 Hz), 50.3, 37.4 (t, J = 20.6 Hz), 35.2, 26.6 (d, J = 7.3 Hz), 24.9 (d, J = 7.5 Hz), 21.6.

^{19}F NMR (376 MHz, CDCl$_3$) δ -108.85 (dd, J = 279.1, 13.2 Hz), -111.20, -112.26 (d, J = 1354.0 Hz).

M.p. 133.0– 133.6 °C

HRMS (ESI-TOF) m/z: $[M+H]^+$ Calcd. for $C_{20}H_{19}F_3NO_2$ 362.1362; found: 362.1368.

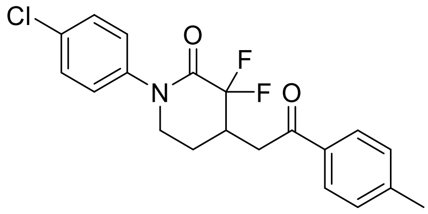

1-(4-chlorophenyl)-3,3-difluoro-4-(2-oxo-2-(*p*-tolyl)ethyl)piperidin-2-one (3hb)

Upon completion the mixture was concentrated and purified via flash column chromatography (petroleum ether : ethyl acetate = 5 : 1, Rf = 0.4) to give the titled product **3hb** as a yellow solid (64.1 mg, 85%).

^{1}H NMR (400 MHz, CDCl$_3$) δ 7.90 (d, J = 8.2 Hz, 2H), 7.39 (d, J = 8.7 Hz, 2H), 7.29 (d, J = 8.1 Hz, 2H), 7.25 (d, J = 8.7 Hz, 2H), 3.96 – 3.88 (m, 1H), 3.62 – 3.57 (m, 1H), 3.51 (d, J = 15.8 Hz, 1H), 3.18 – 3.05 (m, 2H), 2.43 (s, 3H), 2.29 – 2.24 (m, 1H), 2.06 – 1.95 (m, 1H).

^{13}C NMR (101 MHz, CDCl$_3$) δ 196.4, 161.2 (t, J = 30.2 Hz), 144.7, 139.5, 133.9, 133.3, 129.5, 129.4, 128.2, 126.9, 113.5 (dd, J = 249.1, 244.9 Hz), 50.0, 37.4 (t, J = 20.7 Hz), 35.1, 24.9 (d, J = 7.6 Hz), 21.7.

^{19}F NMR (376 MHz, CDCl$_3$) δ -108.17 (dd, J = 279.0, 23.9 Hz), -110.26 (d, J = 278.9 Hz).

M.p. 142.0– 142.5 °C

HRMS (ESI-TOF) m/z: $[M+H]^+$ Calcd. for $C_{20}H_{17}ClF_2NO_2$ 378.1067; found: 378.1069.

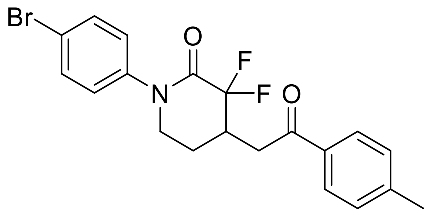

1-(4-bromophenyl)-3,3-difluoro-4-(2-oxo-2-(*p*-tolyl)ethyl)piperidin-2-one (3ib)

Upon completion the mixture was concentrated and purified via flash column chromatography (petroleum ether : ethyl acetate = 5 : 1, Rf = 0.4) to give the titled product **3ib** as a white solid (60.8 mg, 72%).

^{1}H NMR (400 MHz, CDCl$_3$) δ 7.90 (d, J = 8.2 Hz, 2H), 7.54 (d, J = 8.7 Hz, 2H), 7.29 (d, J = 8.0 Hz, 2H), 7.19 (d, J = 8.7 Hz, 2H), 3.95 – 3.88 (m, 1H), 3.62 – 3.57 (m, 1H), 3.51 (d, J = 15.8 Hz, 1H), 3.18 – 3.04 (m, 2H), 2.43 (s, 3H), 2.30 – 2.25 (m, 1H), 2.06 – 1.95 (m, 1H).

^{13}C NMR (101 MHz, CDCl$_3$) δ 196.4, 161.2 (t, J = 30.2 Hz), 144.7, 140.0, 133.9, 132.5, 129.4, 128.2, 127.2, 121.2, 113.5 (dd, J = 249.1, 245.1 Hz), 50.0, 37.4 (t, J = 20.6 Hz), 35.2, 24.9 (d, J = 7.6 Hz), 21.7.

^{19}F NMR (376 MHz, CDCl$_3$) δ -108.16 (dd, J = 279.0, 23.4 Hz), -110.29 (d, J = 279.1 Hz).

M.p. 139.0– 139.5 °C

HRMS (ESI-TOF) m/z: [M+H]$^+$ Calcd. for $C_{20}H_{17}BrF_2NO_2$ 421.0489; found: 421.0493.

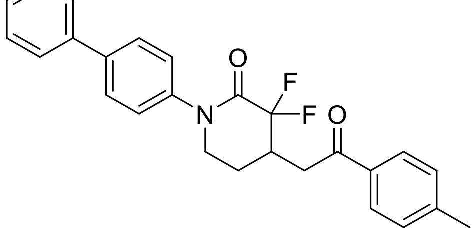

1-([1,1'-biphenyl]-4-yl)-3,3-difluoro-4-(2-oxo-2-(*p*-tolyl)ethyl)piperidin-2-one (3jb)

Upon completion the mixture was concentrated and purified via flash column chromatography (petroleum ether : ethyl acetate = 5 : 1, Rf = 0.4) to give the titled product **3jb** as a white solid (52.9 mg, 63%).

^{1}H NMR (400 MHz, CDCl$_3$) δ 7.96 (dd, J = 27.8, 8.2 Hz, 2H), 7.64 (d, J = 8.5 Hz, 2H), 7.58 (d, J = 7.2 Hz, 2H), 7.45 (t, J = 7.5 Hz, 2H), 7.39 – 7.35 (m, 3H), 7.31 (d, J = 8.0 Hz, 2H), 4.02 – 3.94 (m, 1H), 3.71 – 3.66 (m, 1H), 3.55 (d, J = 15.7 Hz, 1H), 3.21 – 3.11 (m, 1H), 2.44 (s, 3H), 2.43 (s, 1H), 2.33 – 2.28 (m, 1H), 2.10 – 1.99 (m, 1H).

^{13}C NMR (101 MHz, CDCl$_3$) δ 196.6, 161.2 (d, J = 29.8 Hz), 144.6, 140.7, 140.2, 133.9, 130.2, 129.5, 129.2, 128.8, 128.2, 128.1, 127.6, 127.1, 125.9, 113.7 (dd, J = 233.8, 229.9 Hz), 50.1, 37.5 (t, J = 20.7 Hz), 35.2, 25.0 (d, J = 7.4 Hz), 21.7.

^{19}F NMR (376 MHz, CDCl$_3$) δ -108.22 (dd, J = 278.7, 23.9 Hz), -110.15 (d, J = 278.6 Hz).

M.p. 167.1– 167.7 °C

HRMS (ESI-TOF) m/z: $[M+H]^+$ Calcd. for $C_{26}H_{24}F_2NO_2$ 420.1770; found: 420.1778.

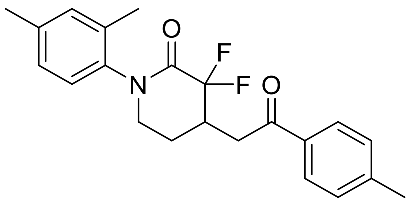

1-(2,4-dimethylphenyl)-3,3-difluoro-4-(2-oxo-2-(*p*-tolyl)ethyl)piperidin-2-one (3kb)

Upon completion the mixture was concentrated and purified via flash column chromatography (petroleum ether : ethyl acetate = 5 : 1, Rf = 0.4) to give the titled product **3kb** as a yellow oil (48.3 mg, 65%) with 95% purity.

^{1}H NMR (400 MHz, CDCl$_3$) δ 7.92 (d, J = 8.1 Hz, 2H), 7.30 (d, J = 7.9 Hz, 2H), 7.17 (d, J = 7.7 Hz, 1H), 7.08 (d, J = 7.8 Hz, 1H), 6.97 (d, J = 5.5 Hz, 1H), 3.91 – 3.68 (m, 1H), 3.60 – 3.37 (m, 2H), 3.21 – 3.05 (m, 2H), 2.44 (s, 3H), 2.32 (d, J = 3.4 Hz, 3H), 2.26 (dd, J = 16.1, 4.9 Hz, 1H), 2.17 (d, J = 5.3 Hz, 3H), 2.10 – 1.95 (m, 1H).

^{13}C NMR (101 MHz, CDCl$_3$) δ 196.6, 160.4 (d, J = 30.6 Hz), 144.6, 139.5, 137.4, 137.1, 133.9, 131.5, 131.4, 131.1, 129.5, 129.3, 128.2, 127.3, 126.6, 111.8 (dd, J = 363.1, 115.7 Hz), 50.0, 49.8, 37.6 (t, J = 20.9 Hz), 35.2, 35.1, 25.2 (d, J = 8.1 Hz). 24.7, 21.7, 20.8, 16.9, 16.7.

^{19}F NMR (376 MHz, CDCl$_3$) δ -107.74 – -109.62 (m), -111.35 (d, J = 278.7 Hz).

HRMS (ESI-TOF) m/z: $[M+H]^+$ Calcd. for $C_{22}H_{24}F_2NO_2$ 372.1770; found: 372.1777.

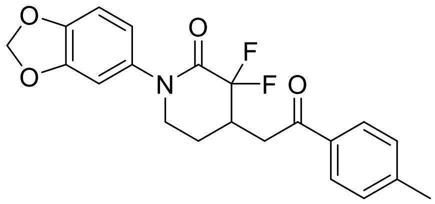

1-(benzo[*d*][1,3]dioxol-5-yl)-3,3-difluoro-4-(2-oxo-2-(*p*-tolyl)ethyl)piperidin-2-one (3lb)

Upon completion the mixture was concentrated and purified via flash column chromatography (petroleum ether : ethyl acetate = 10: 1, Rf = 0.4) to give the titled product **3lb** as a white solid (47.3 mg, 61%).

^{1}H NMR (400 MHz, $CDCl_3$) δ 7.90 (d, *J* = 8.2 Hz, 2H), 7.29 (d, *J* = 8.0 Hz, 2H), 6.82 (d, *J* = 8.2 Hz, 1H), 6.77 (d, *J* = 2.0 Hz, 1H), 6.72 (dd, *J* = 8.2, 2.1 Hz, 1H), 5.99 (s, 2H), 3.90 – 3.82 (m, 1H), 3.60 – 3.55 (m, 1H), 3.51 (d, *J* = 15.8 Hz, 1H), 3.17 – 3.03 (m, 2H), 2.43 (s, 3H), 2.26 – 2.21 (m, 1H), 2.04 – 1.93 (m, 1H).

^{13}C NMR (101 MHz, $CDCl_3$) δ 196.5, 161.4 (t, *J* = 30.0 Hz), 148.2, 147.0, 144.6, 134.9, 133.9, 129.4, 128.2, 119.1, 113.6 (dd, *J* = 248.7, 244.9 Hz), 108.5, 107.3, 101.7, 50.7, 37.4 (t, *J* = 20.6 Hz), 35.2, 24.9 (d, *J* = 7.5 Hz), 21.6.

^{19}F NMR (376 MHz, $CDCl_3$) δ -108.35 (dd, *J* = 278.8, 23.6 Hz), -110.15 (d, *J* = 278.6 Hz).

M.p. 160.7– 161.4 ℃

HRMS (ESI-TOF) m/z: $[M+H]^+$ Calcd. for $C_{21}H_{20}F_2NO_4$ 388.1355; found: 388.1359.

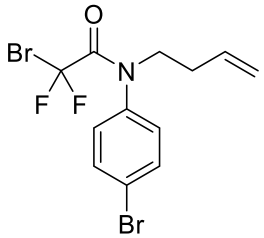

2-bromo-*N*-(4-bromophenyl)-*N*-(but-3-en-1-yl)-2,2-difluoroacetamide (1i)

Upon completion the mixture was concentrated and purified via flash column chromatography (petroleum ether : ethyl acetate = 20: 1, Rf = 0.4) to give the titled product **1i** as a yellow oil with 95% purity.

^{1}H NMR (400 MHz, CDCl$_3$) δ 7.56 (d, J = 8.6 Hz, 1H), 7.16 (d, J = 8.4 Hz, 2H), 5.80 – 5.69 (m, 2H), 5.13 – 5.08 (m, 2H), 3.79 (t, J = 7.3 Hz, 2H), 2.34 (dd, J = 14.1, 7.0 Hz, 2H).

^{13}C NMR (101 MHz, CDCl$_3$) δ 158.8 (t, J = 24.6 Hz), 138.6, 134.0, 132.6, 130.5, 123.0, 117.7, 108.3 (dd, J = 284.1, 195.4 Hz), 51.8, 31.2, 29.3.

^{19}F NMR (376 MHz, CDCl$_3$) δ -51.88.

HRMS (ESI-TOF) m/z: $[M+H]^+$ Calcd. for $C_{12}H_{12}Br_2F_2NO$ 381.9248; found: 381.9256.

4. Reference

(1) Mai, W.-P.; Wang, F.; Zhang, X.-F.; Wang, S.-M.; Duan, Q.-P.; Lu, K. *Org. Biomol. Chem.* **2018**, *16*, 6491-6498.

5. Copy of ^{1}H and ^{13}C NMR Spectra of Products and 1i

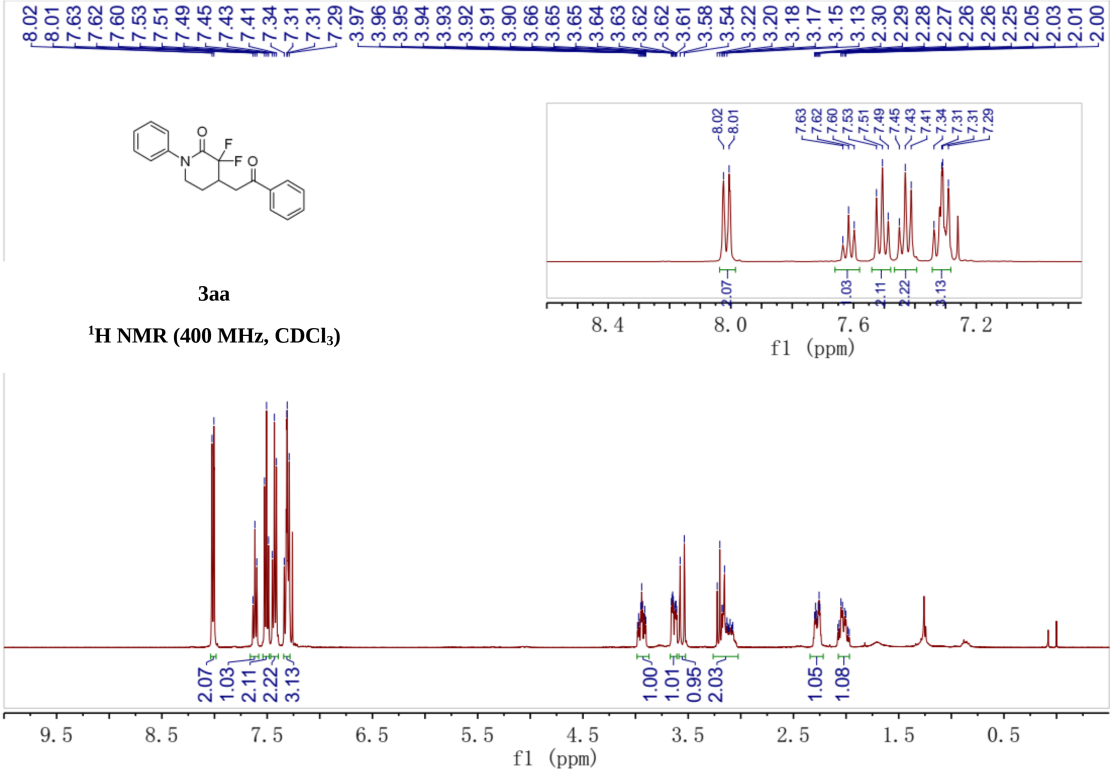

3aa

^{1}H NMR (400 MHz, $CDCl_3$)

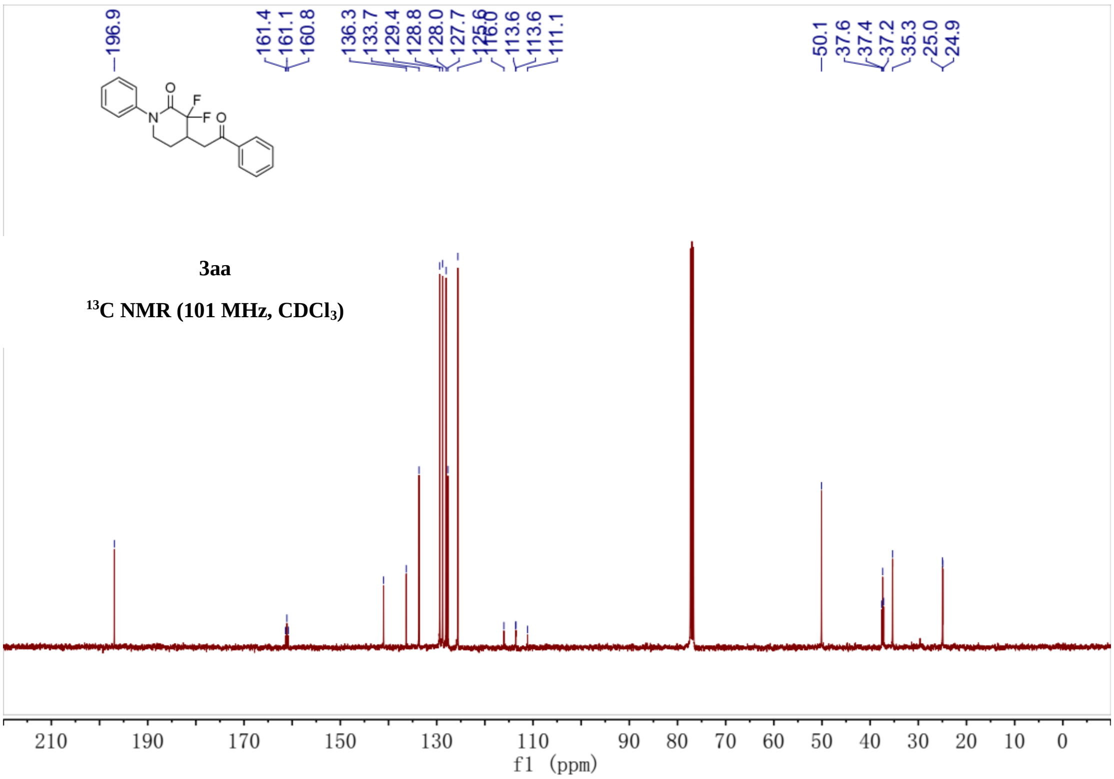

3aa

^{13}C NMR (101 MHz, $CDCl_3$)

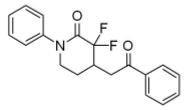

3aa

^{1}F NMR (376 MHz, $CDCl_3$)

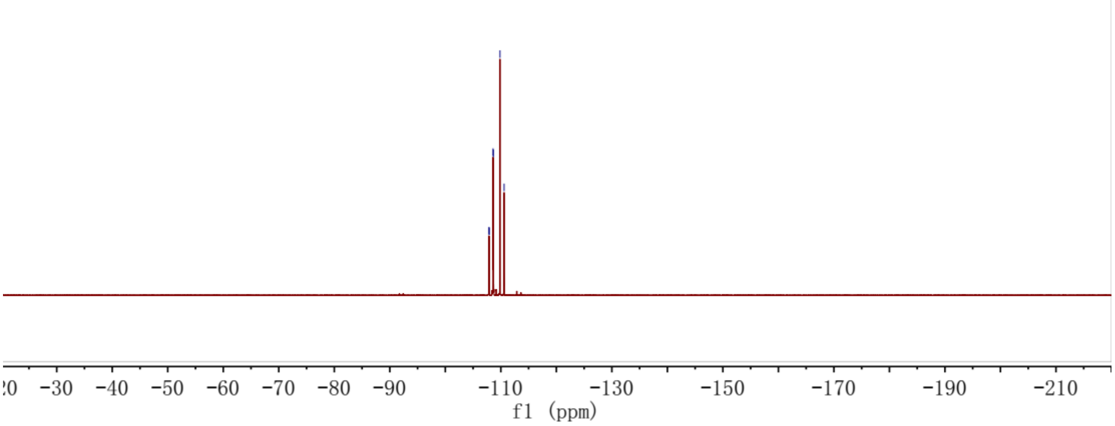

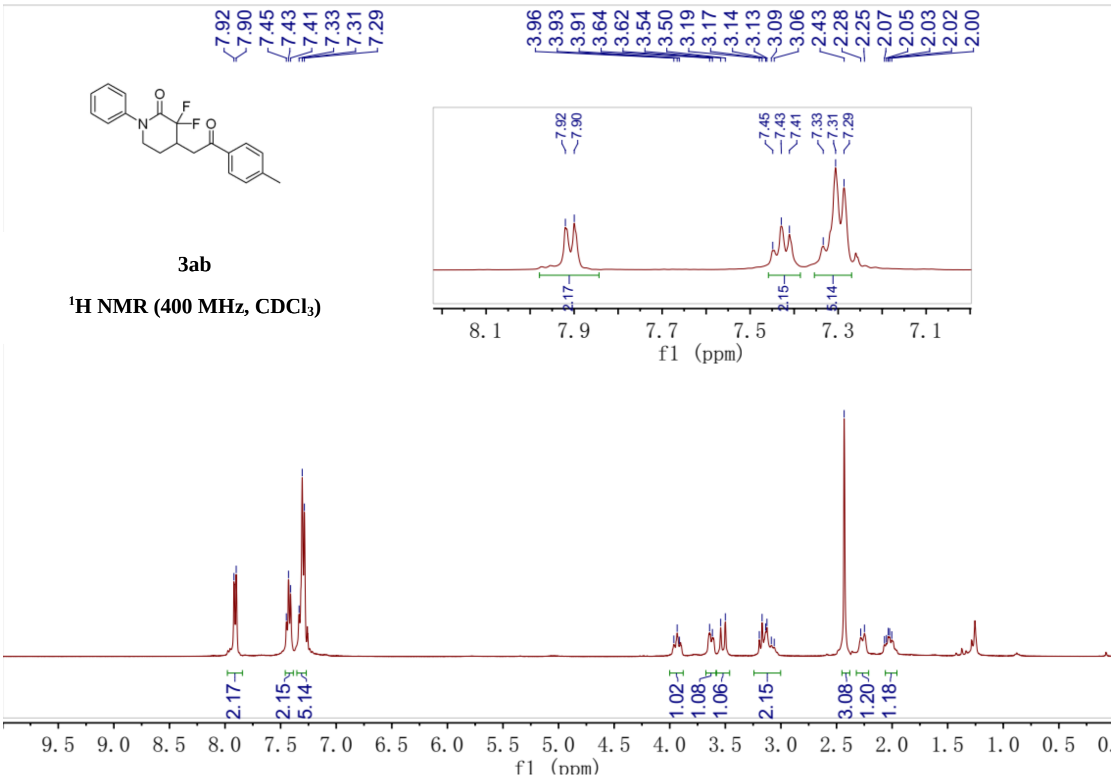

3ab

^{1}H NMR (400 MHz, $CDCl_3$)

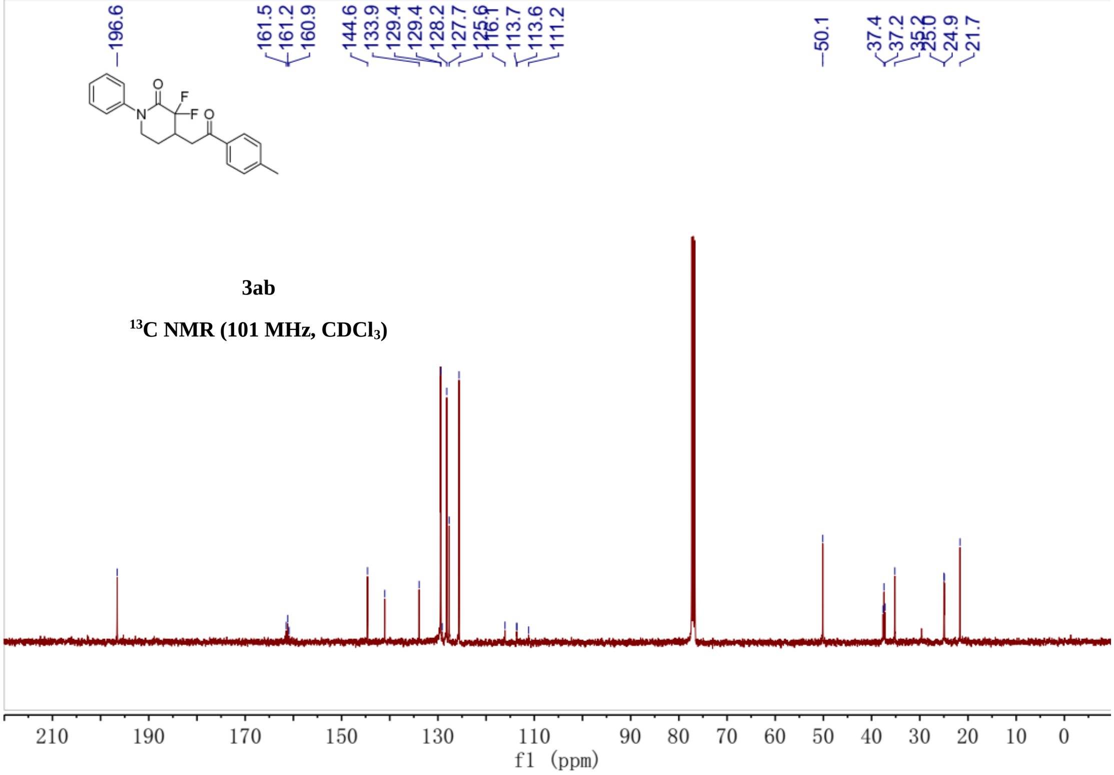

3ab

^{13}C NMR (101 MHz, $CDCl_3$)

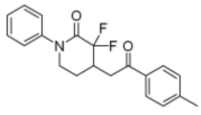

3ab

1F NMR (376 MHz, CDCl3)

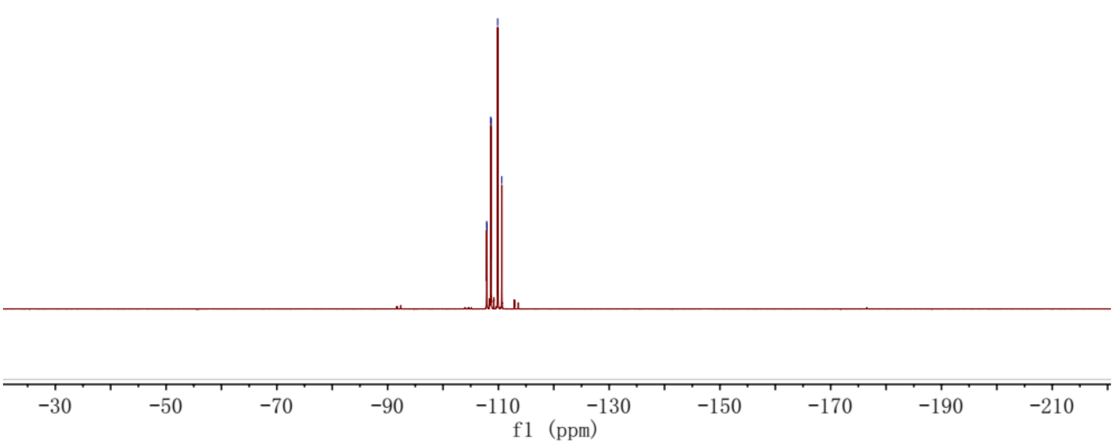

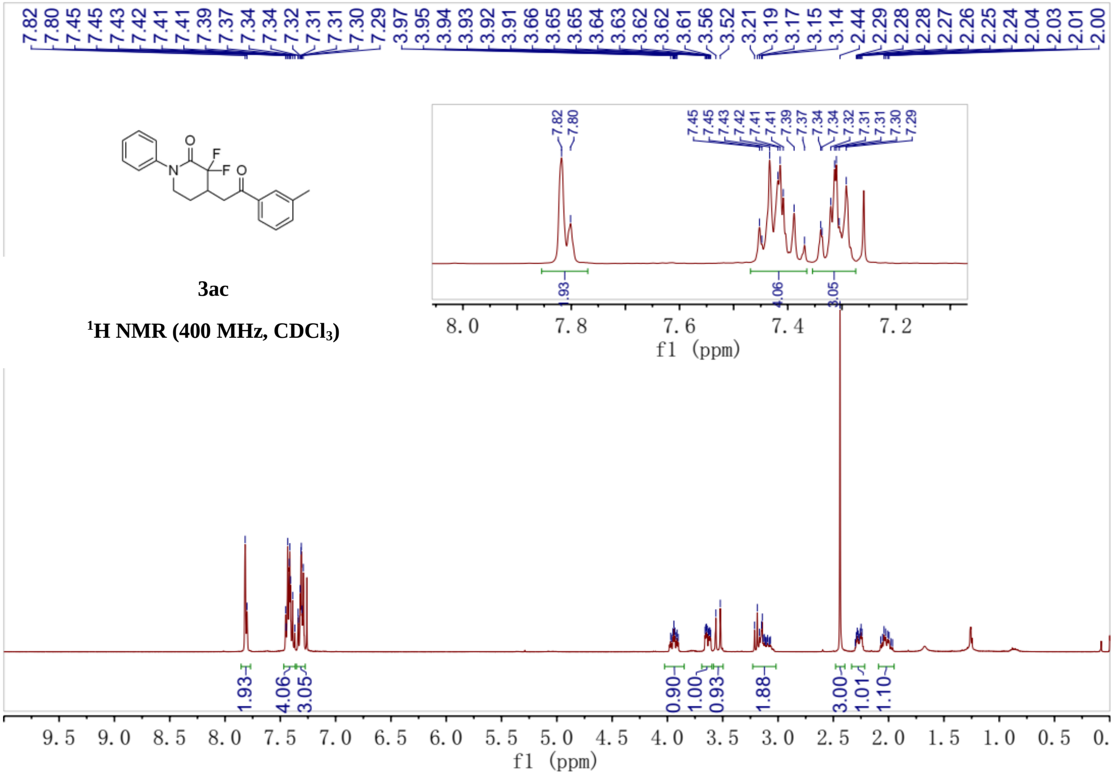

3ac

¹H NMR (400 MHz, CDCl₃)

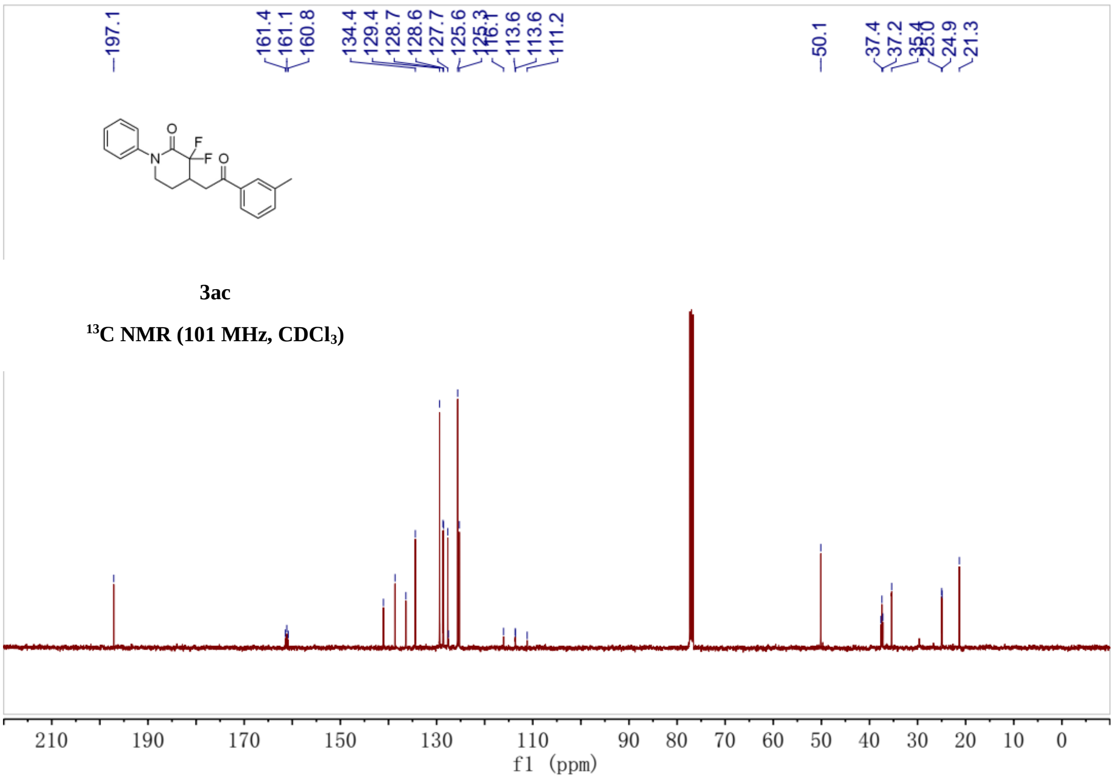

3ac

¹³C NMR (101 MHz, CDCl₃)

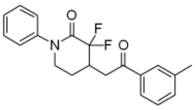

3ac

^{1}F NMR (376 MHz, $CDCl_3$)

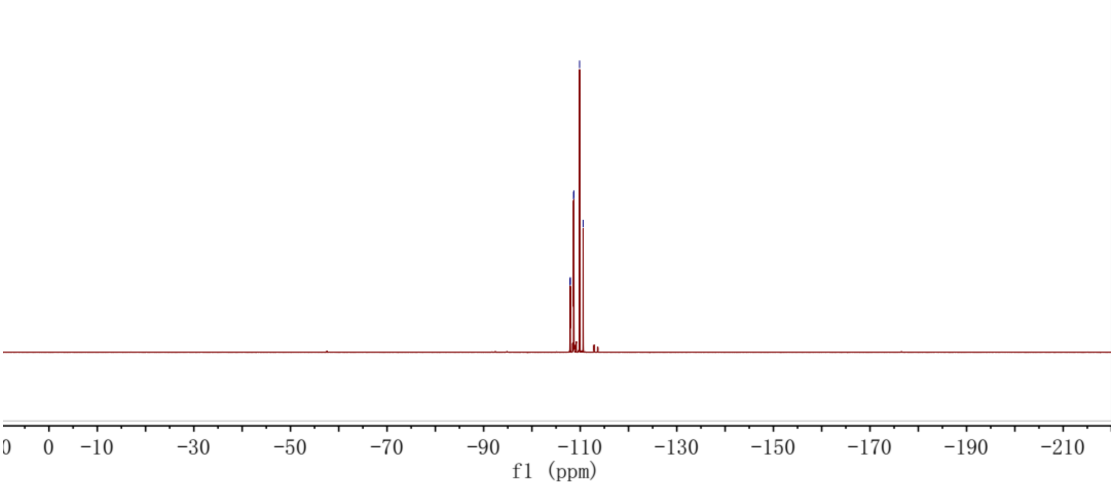

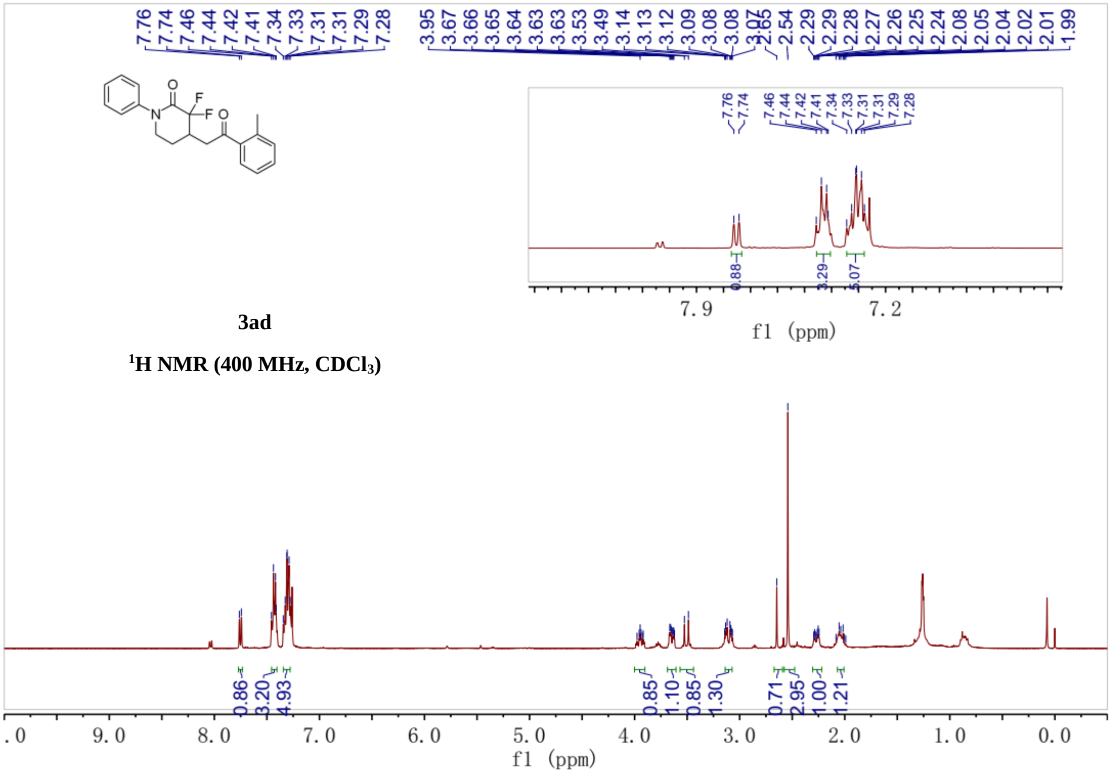

3ad

^{1}H NMR (400 MHz, $CDCl_3$)

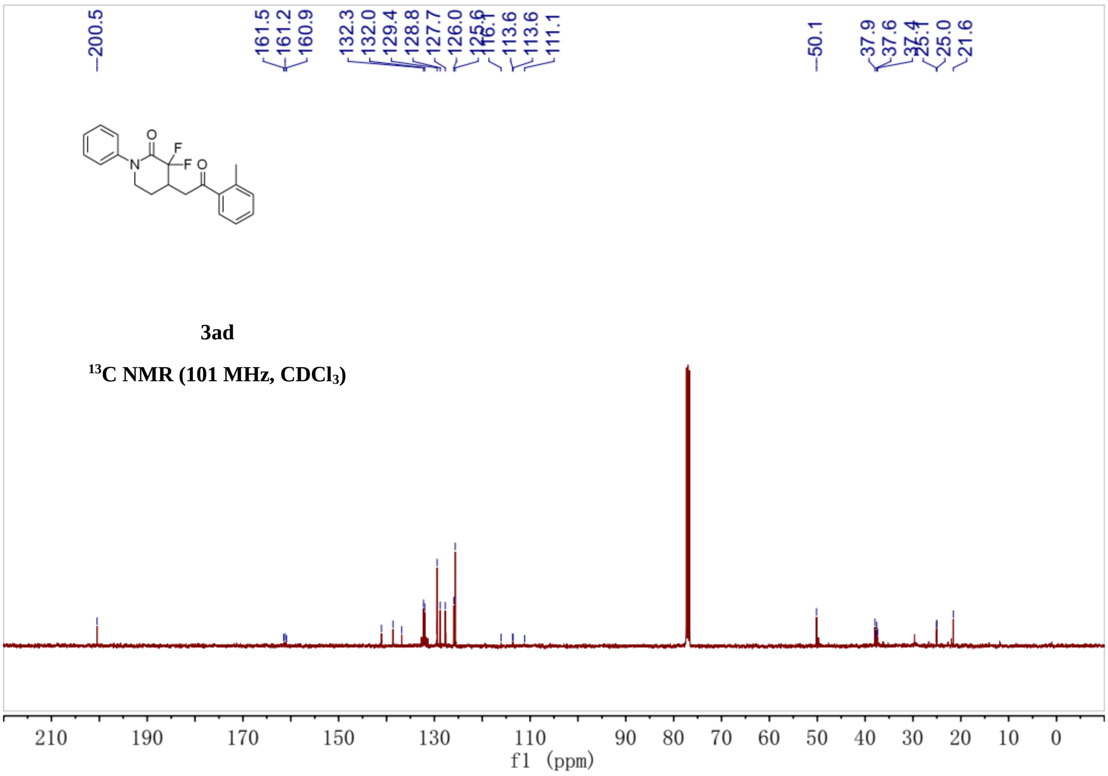

3ad

^{13}C NMR (101 MHz, $CDCl_3$)

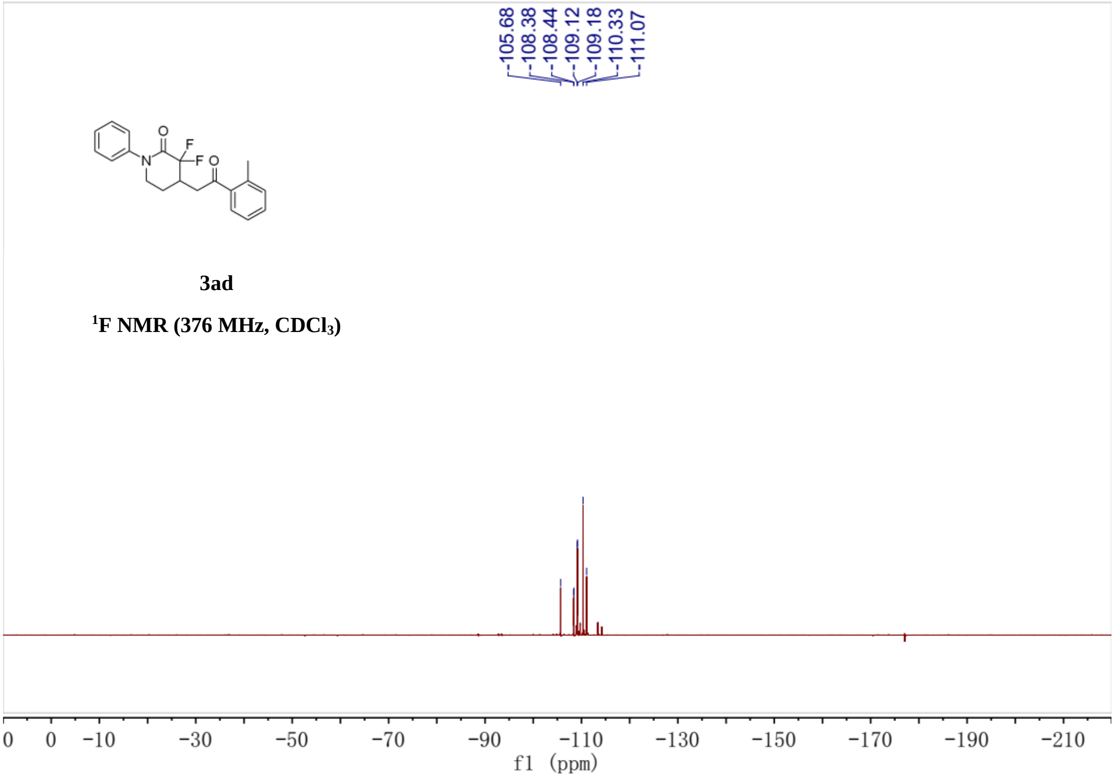

105.68
108.38
108.44
109.12
109.18
110.33
111.07
3ad
1F NMR (376 MHz, CDCl3)
0 0 −10 −30 −50 −70 −90 −110 −130 −150 −170 −190 −210
f1 (ppm)

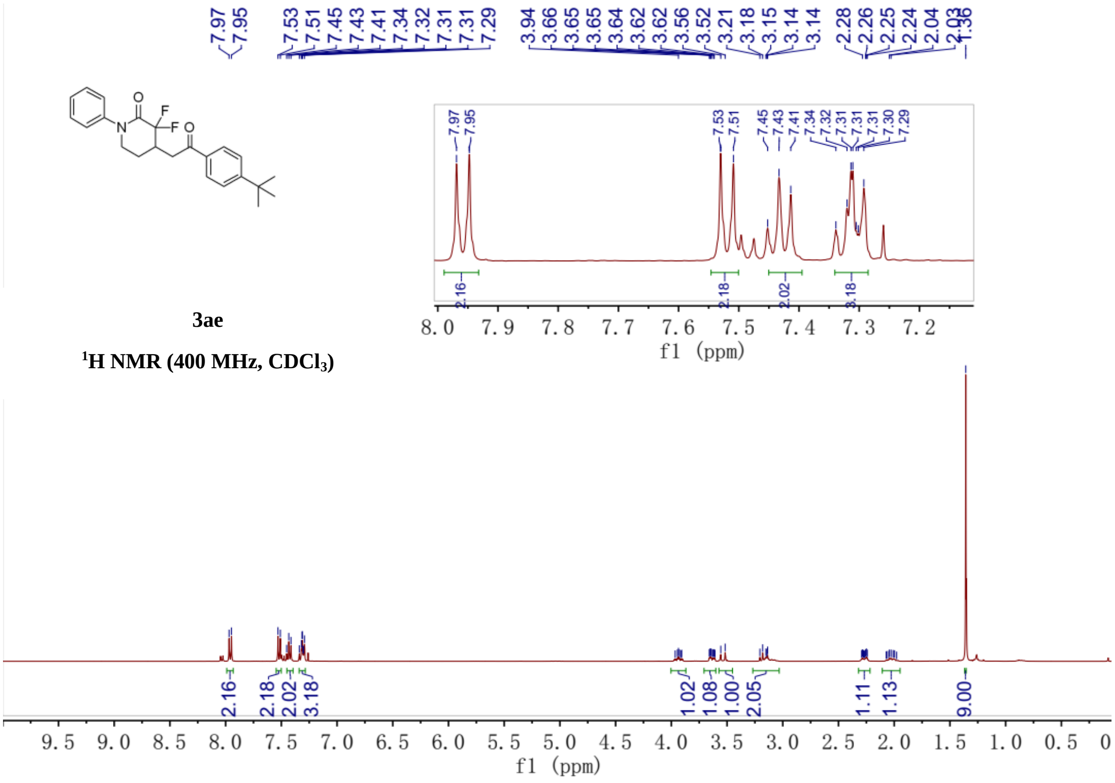

3ae

^{1}H NMR (400 MHz, CDCl$_3$)

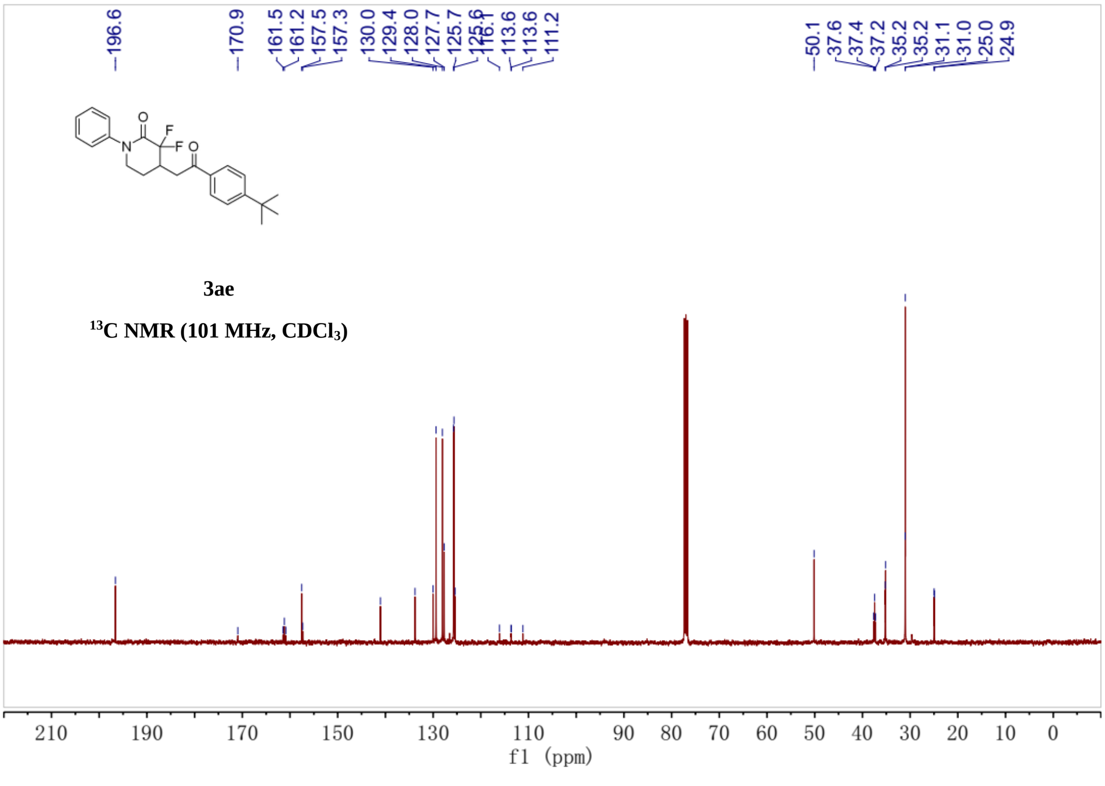

3ae

^{13}C NMR (101 MHz, CDCl$_3$)

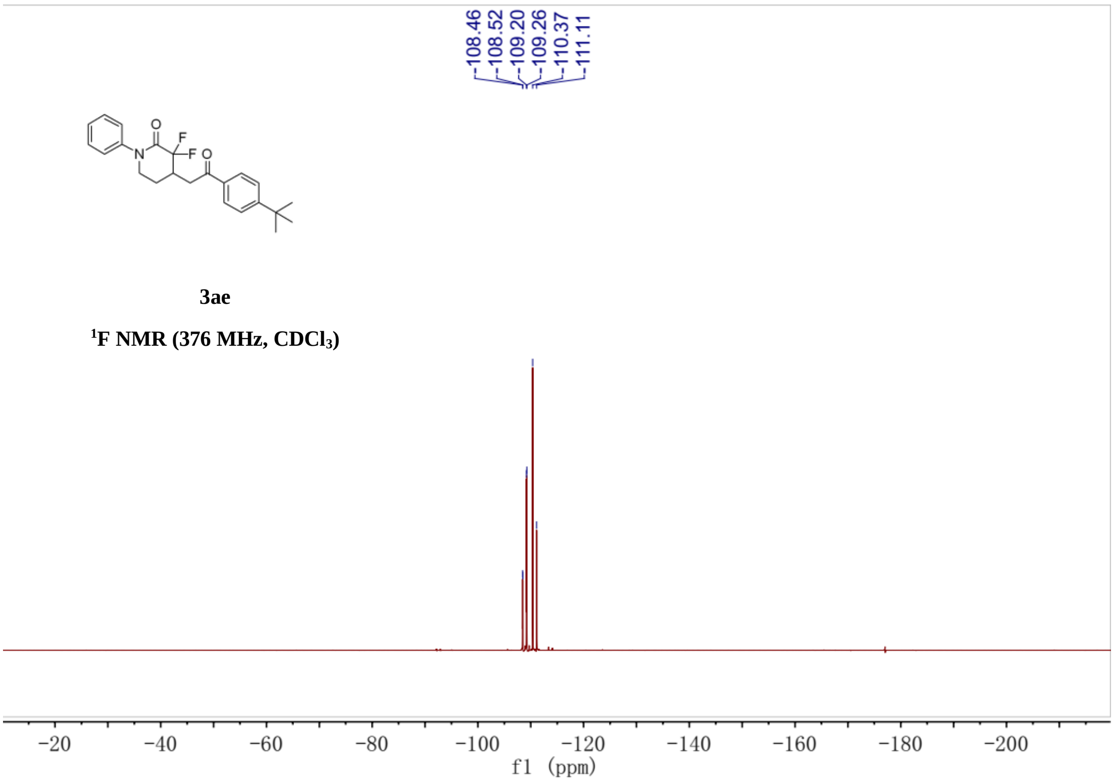

3ae

^{1}F NMR (376 MHz, CDCl$_3$)

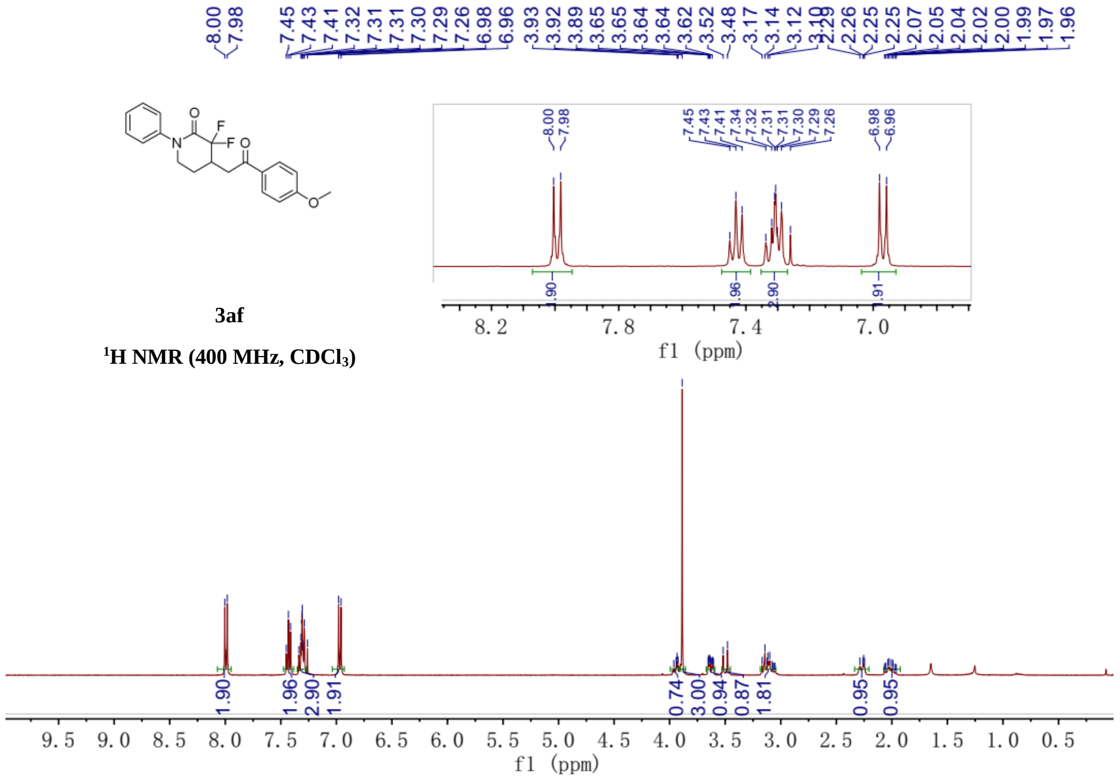

3af

^{1}H NMR (400 MHz, $CDCl_3$)

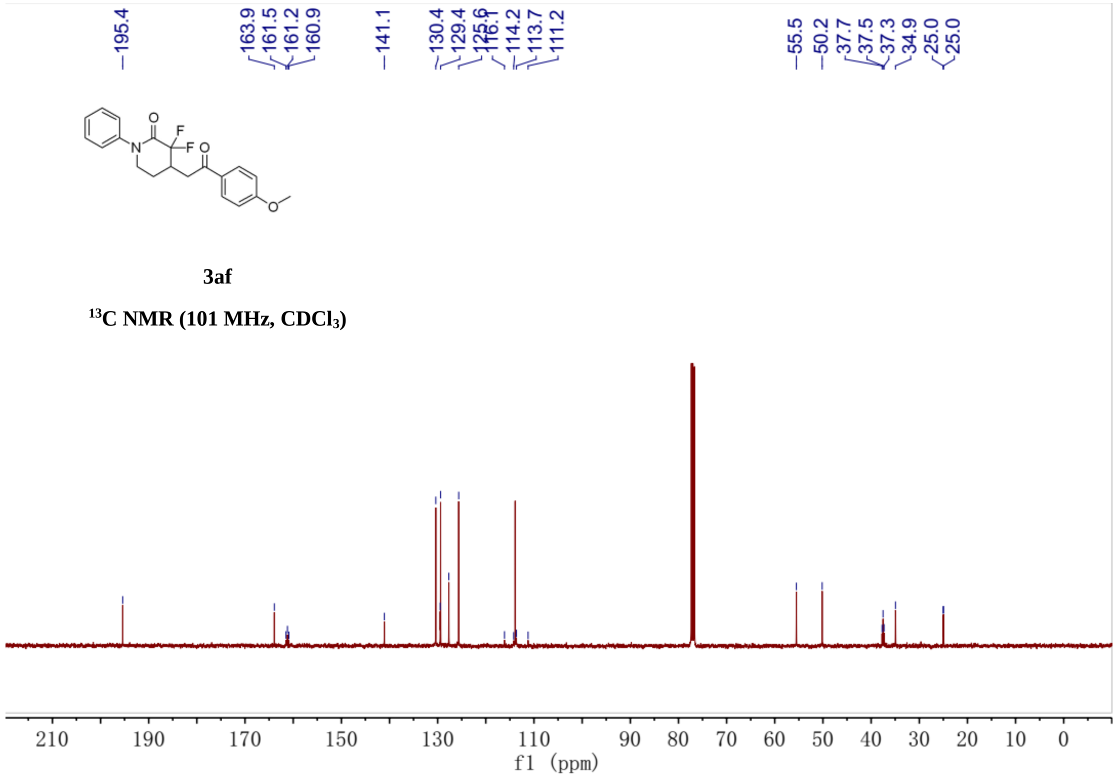

3af

^{13}C NMR (101 MHz, $CDCl_3$)

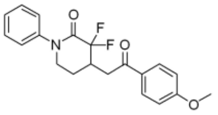

3af

[1]F NMR (376 MHz, CDCl$_3$)

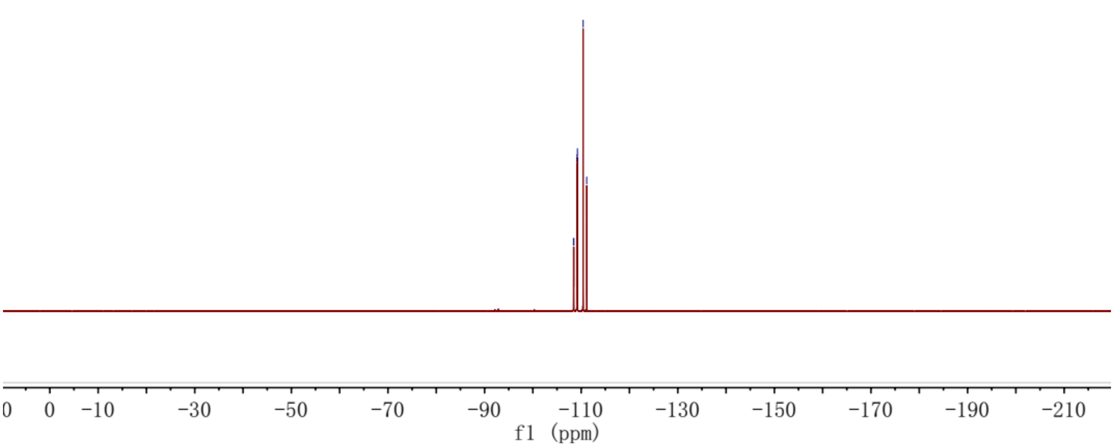

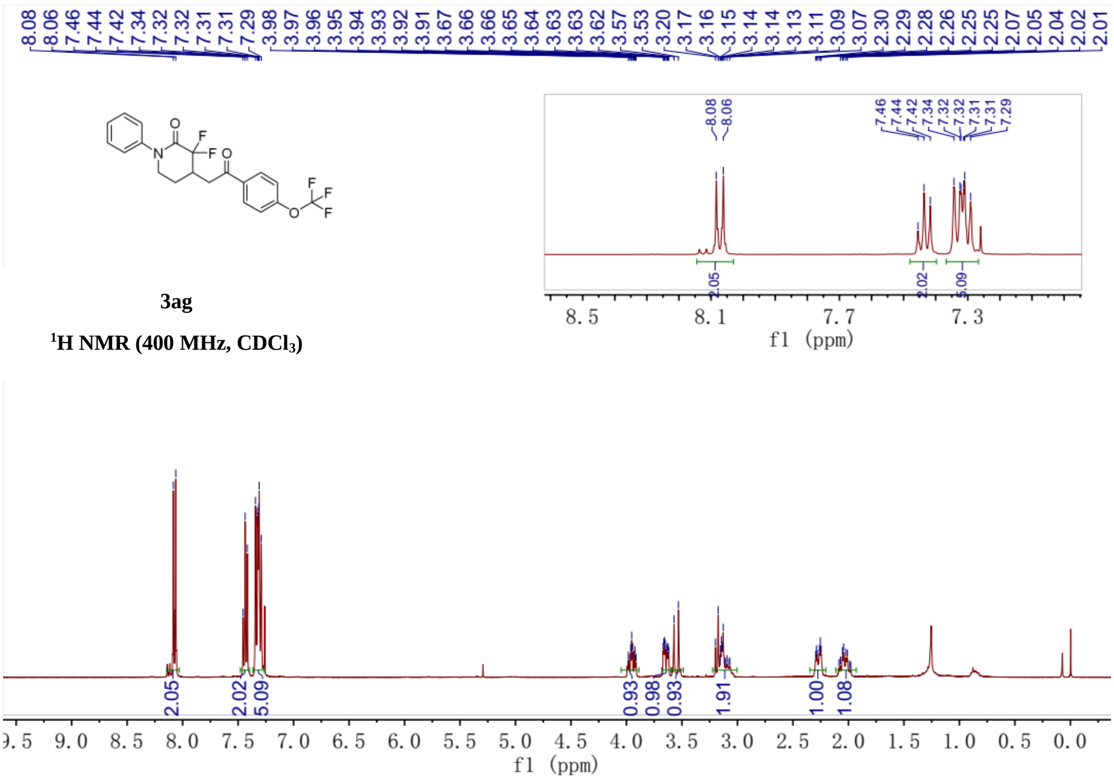

3ag

^{1}H NMR (400 MHz, $CDCl_3$)

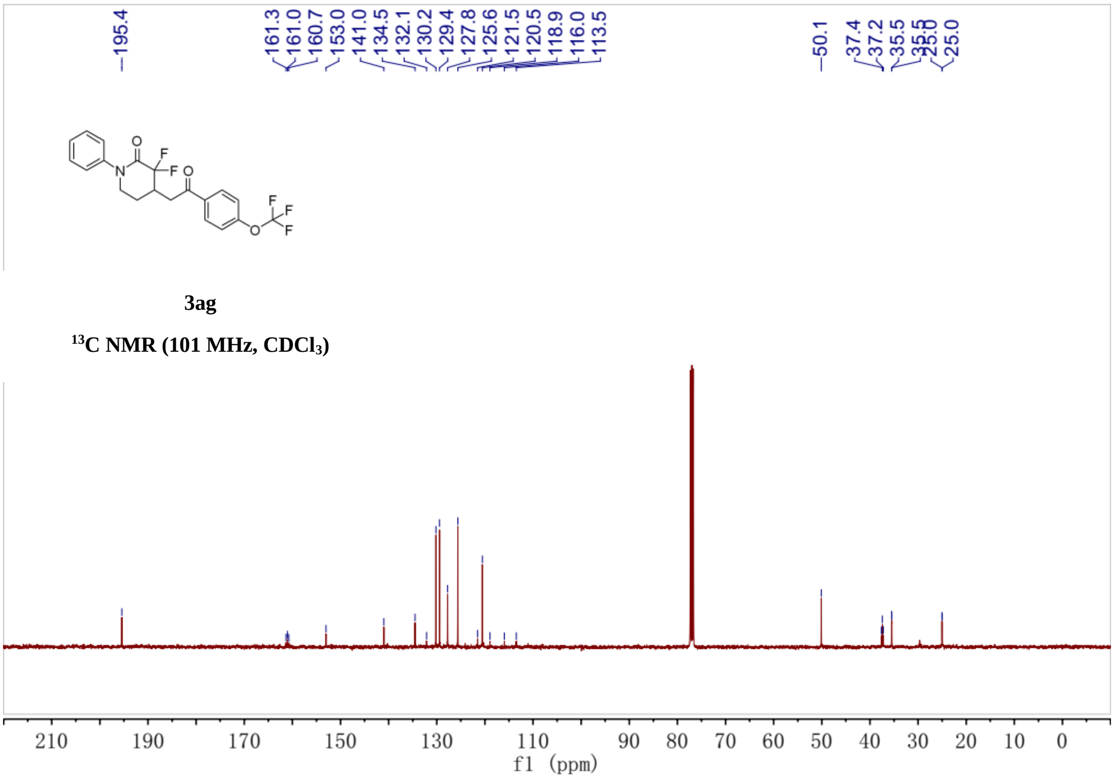

3ag

^{13}C NMR (101 MHz, $CDCl_3$)

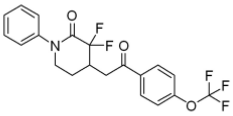

3ag

^{1}F NMR (376 MHz, $CDCl_3$)

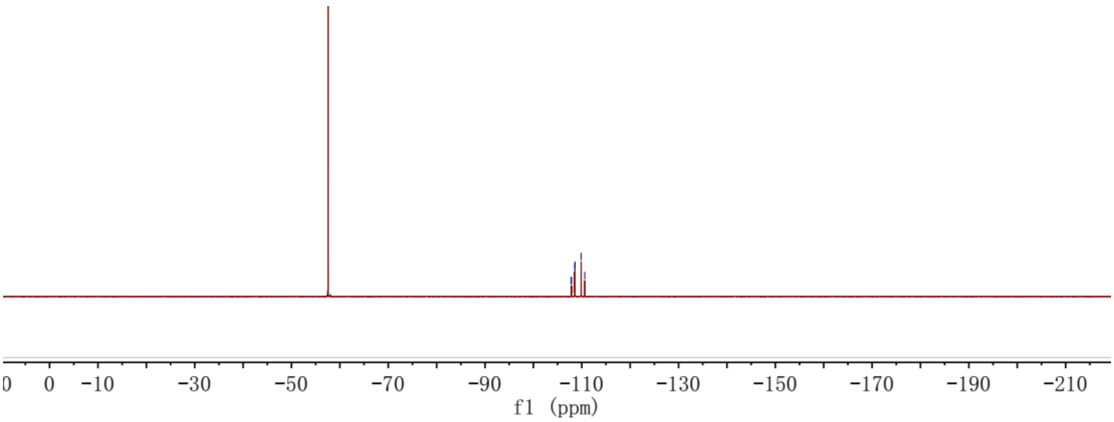

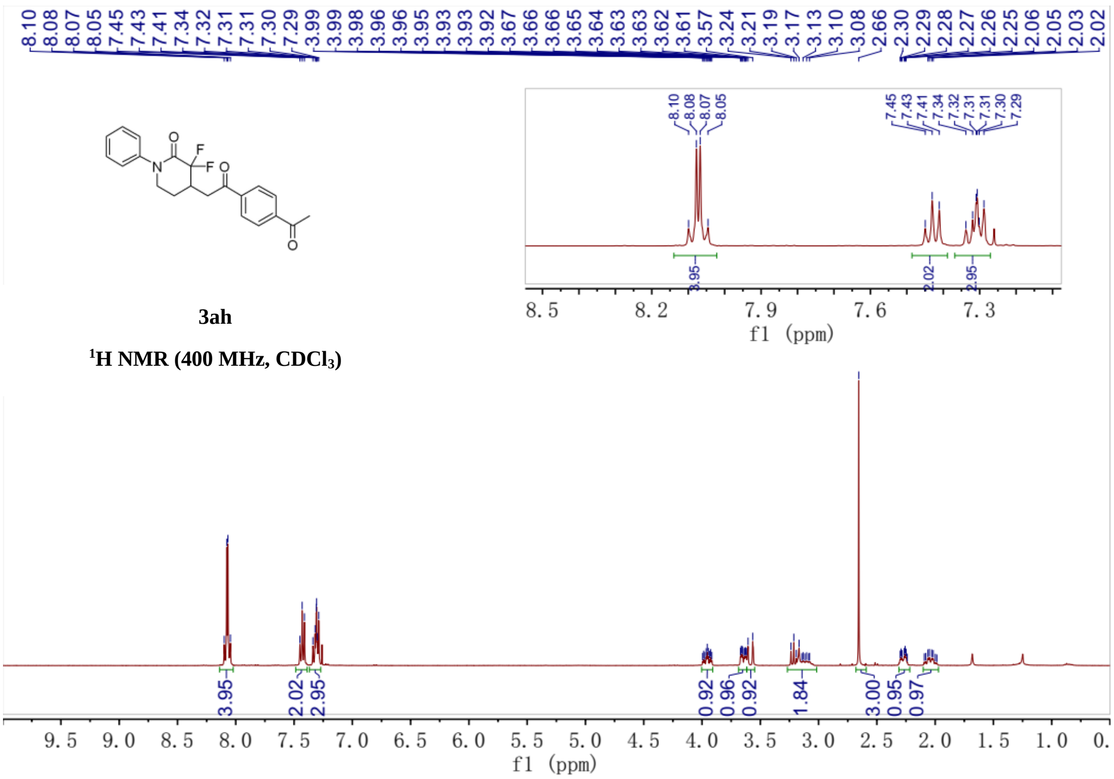

3ah

^{1}H NMR (400 MHz, $CDCl_3$)

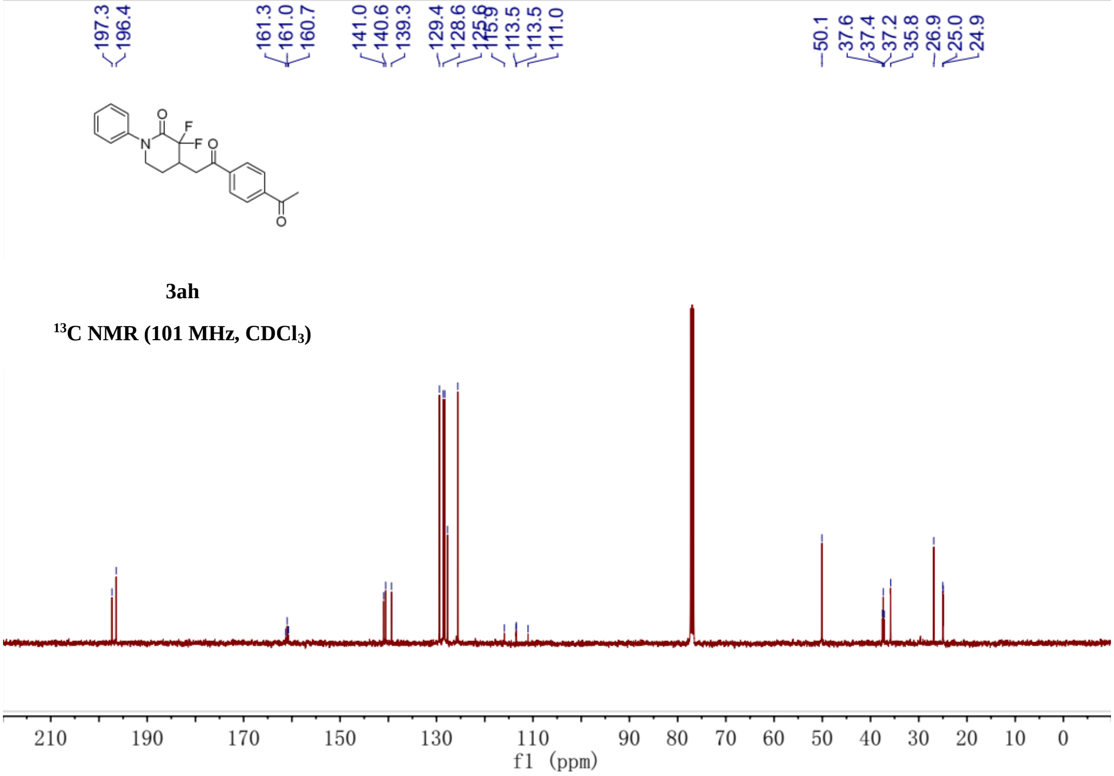

3ah

^{13}C NMR (101 MHz, $CDCl_3$)

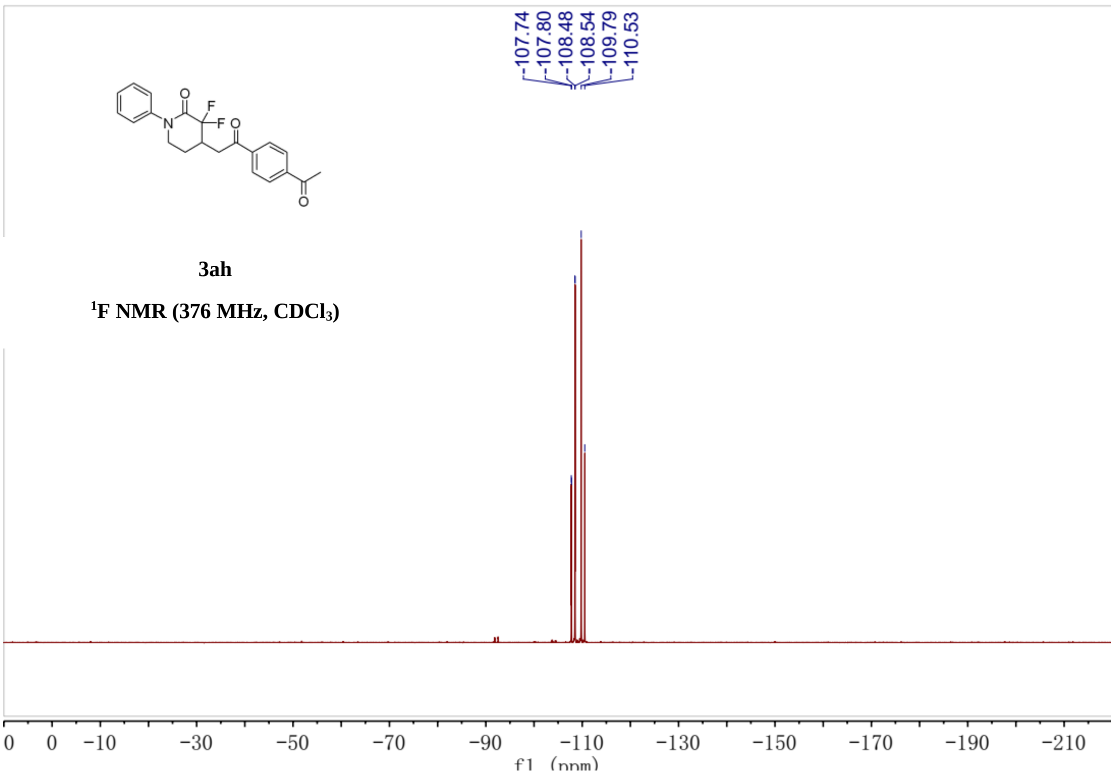

3ah

^{1}F NMR (376 MHz, $CDCl_3$)

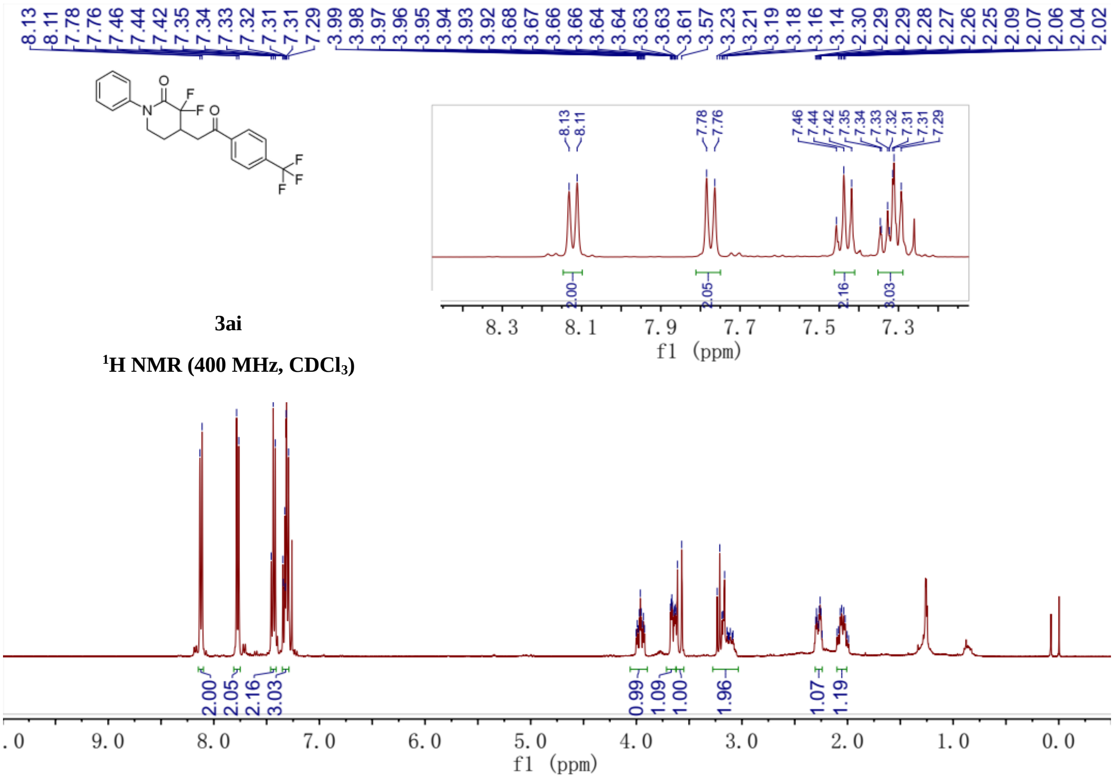

3ai

^{1}H NMR (400 MHz, $CDCl_3$)

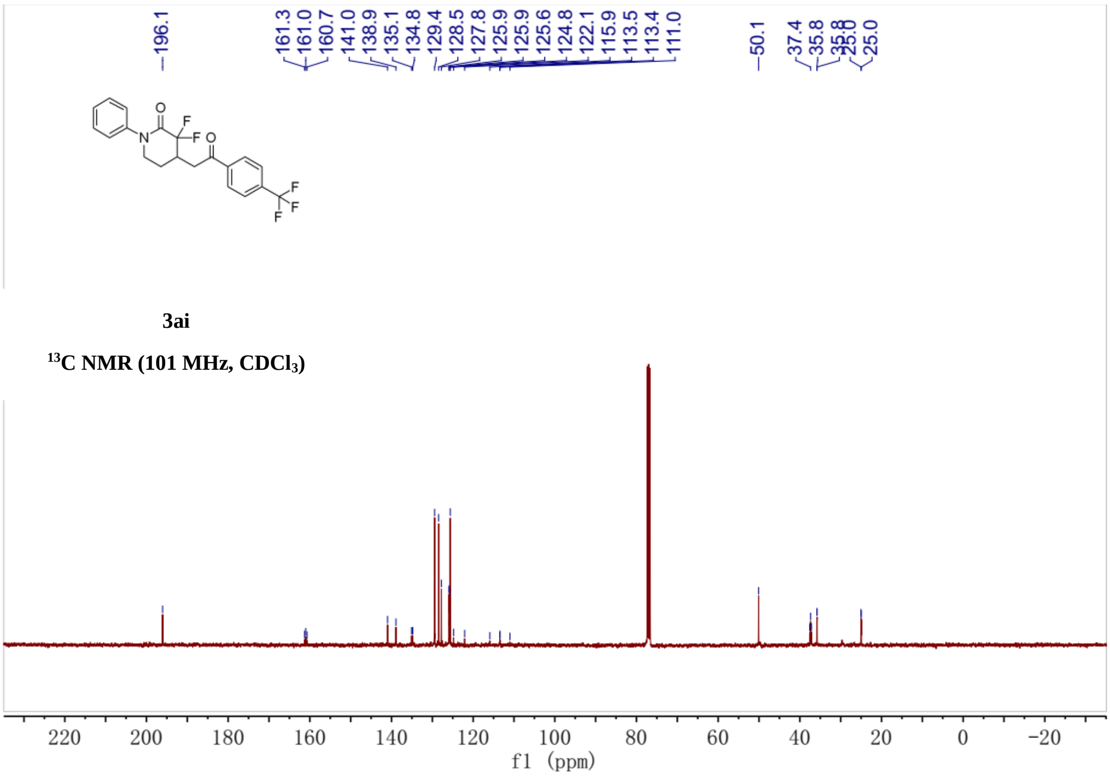

3ai

^{13}C NMR (101 MHz, $CDCl_3$)

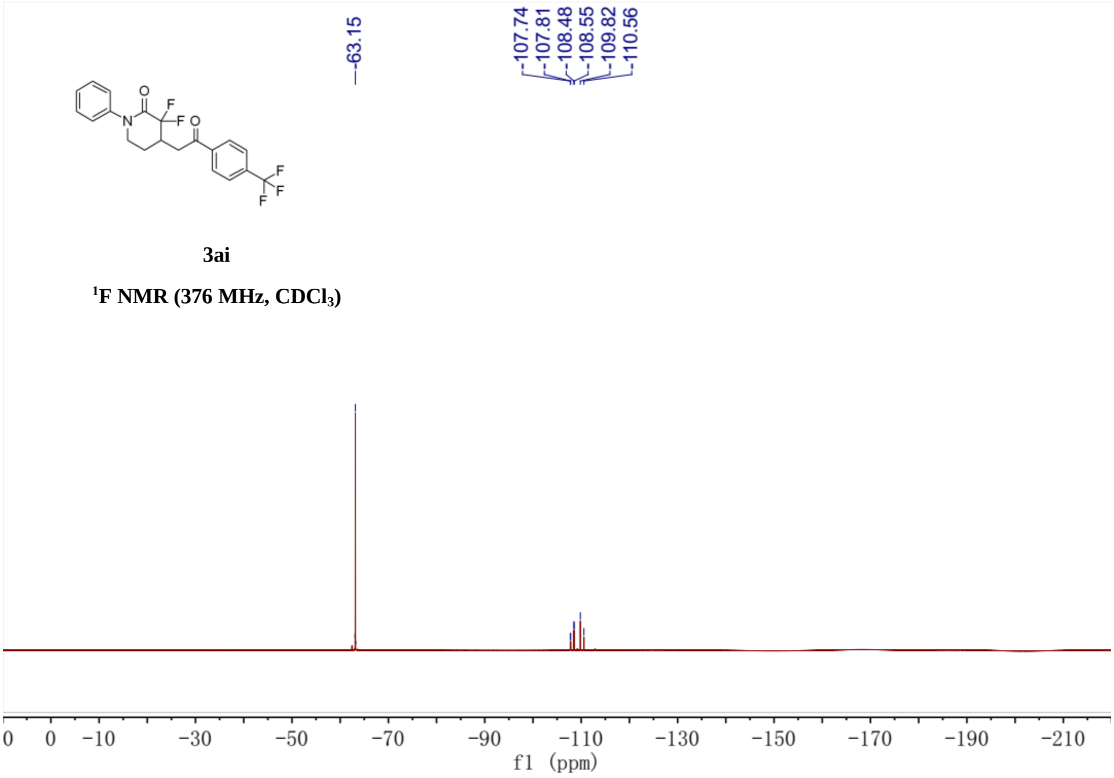

-63.15
-107.74
-107.81
-108.48
-108.55
-109.82
-110.56
3ai
1F NMR (376 MHz, CDCl3)
0
0
−10
−30
−50
−70
−90
−110
−130
−150
−170
−190
−210
f1 (ppm)

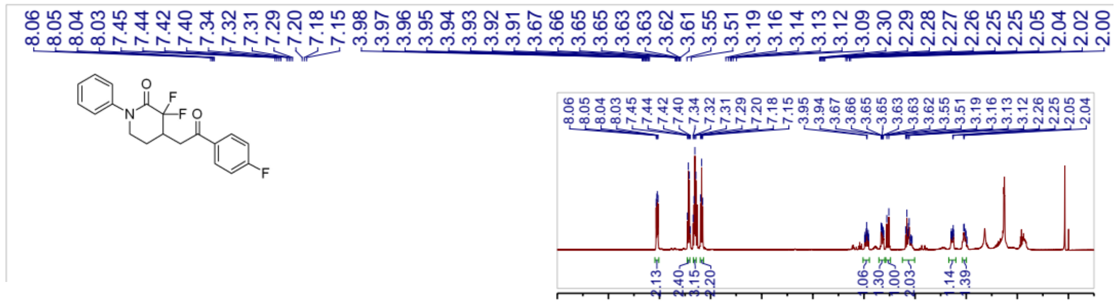

3aj

^{1}H NMR (400 MHz, CDCl$_3$)

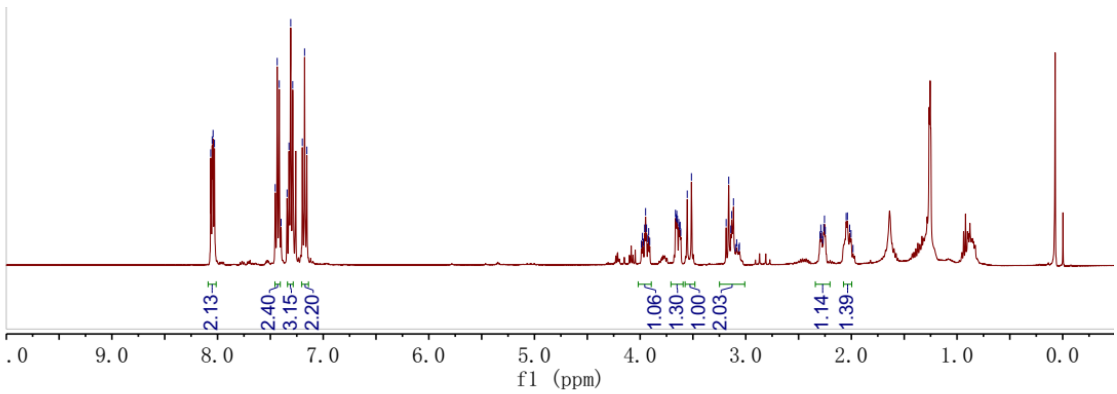

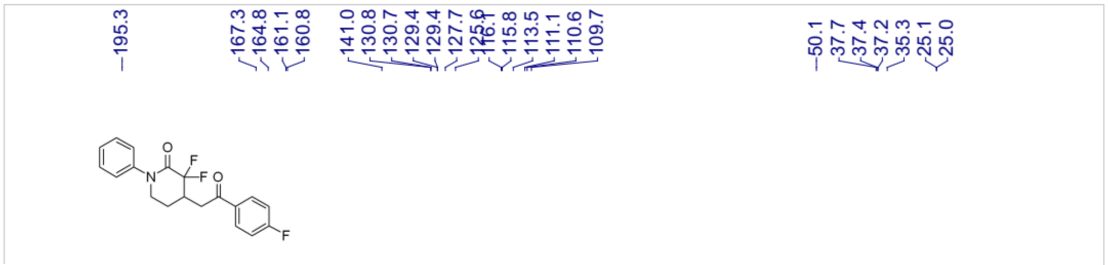

3aj

^{13}C NMR (101 MHz, CDCl$_3$)

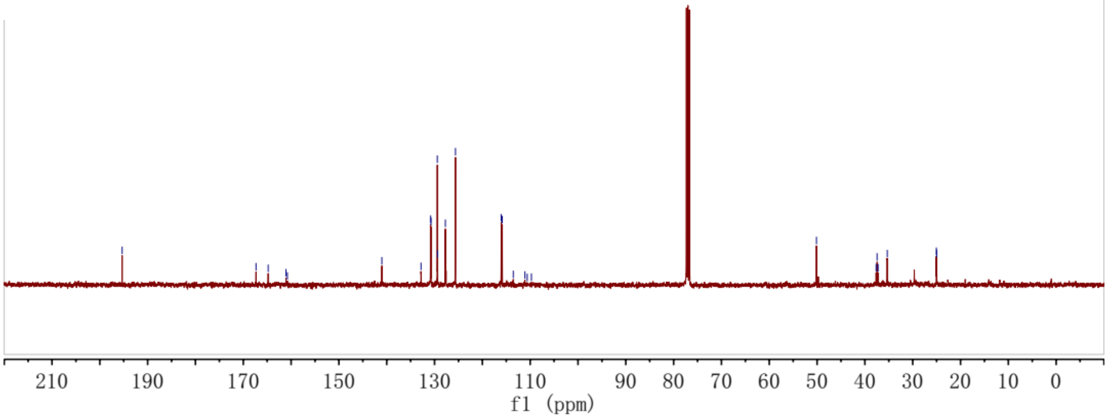

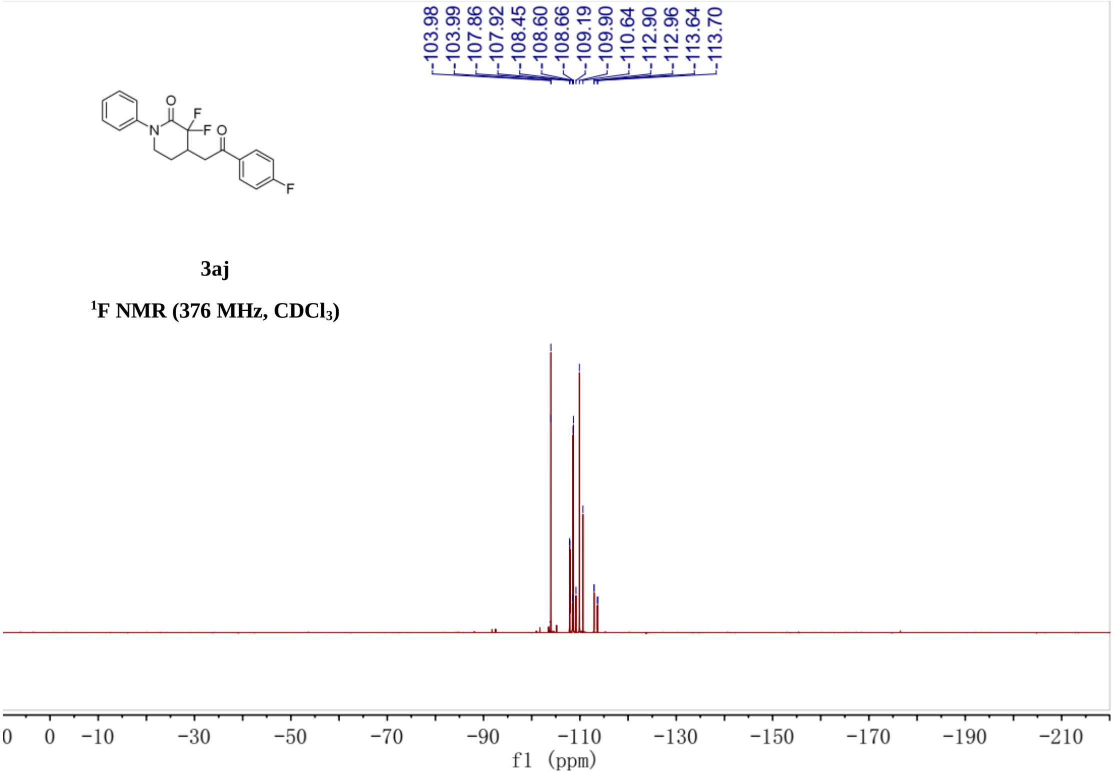

-103.98
-103.99
-107.86
-107.92
-108.45
-108.60
-108.66
-109.19
-109.90
-110.64
-112.90
-112.96
-113.64
-113.70
3aj
^{1}F NMR (376 MHz, $CDCl_3$)
0 0 −10 −30 −50 −70 −90 −110 −130 −150 −170 −190 −210
f1 (ppm)

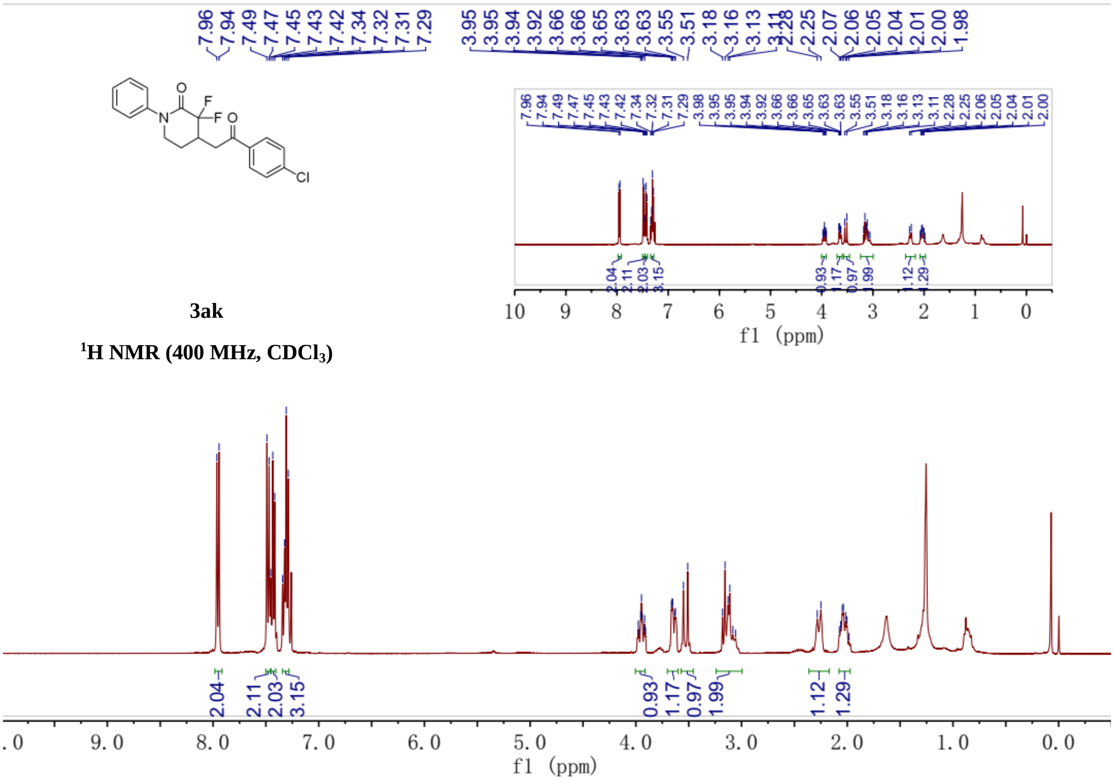

3ak

^{1}H NMR (400 MHz, $CDCl_3$)

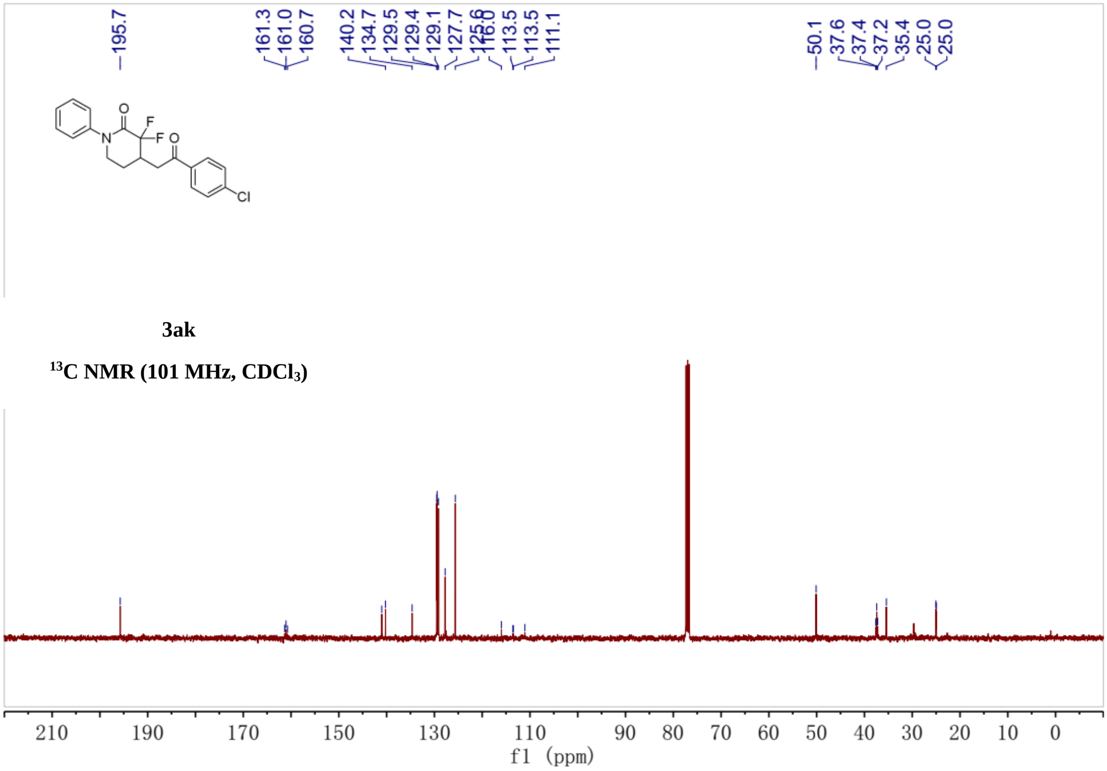

3ak

^{13}C NMR (101 MHz, $CDCl_3$)

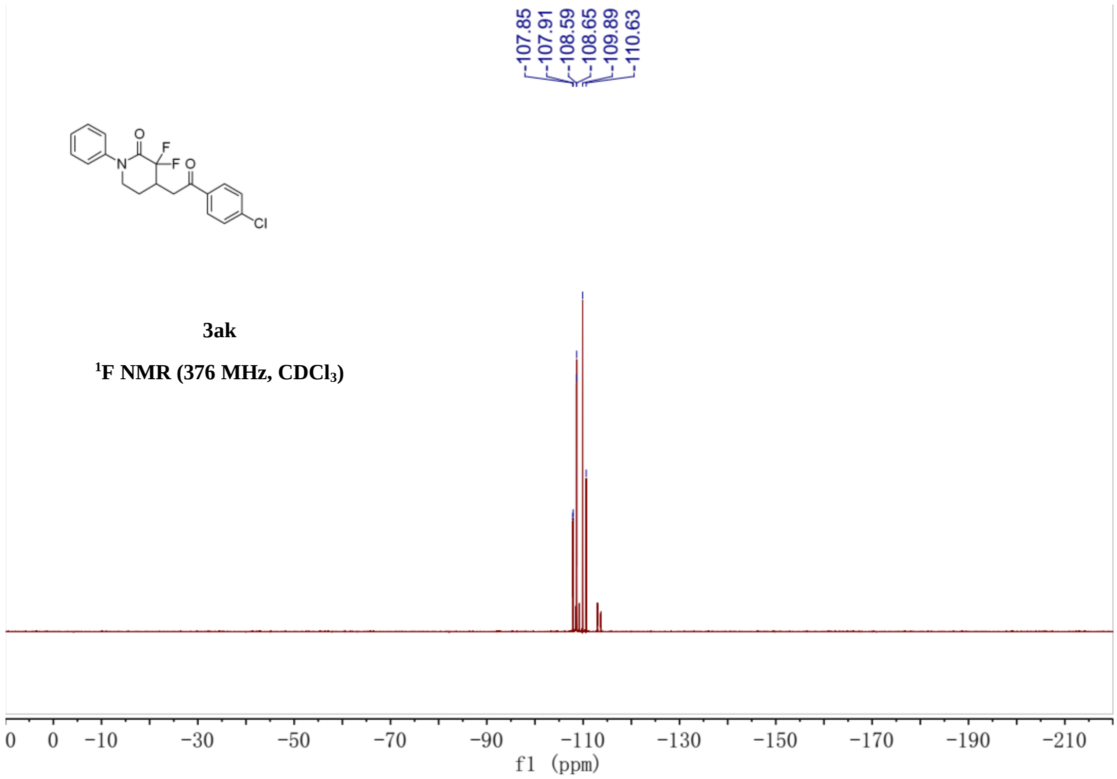

3ak

^{1}F NMR (376 MHz, CDCl$_3$)

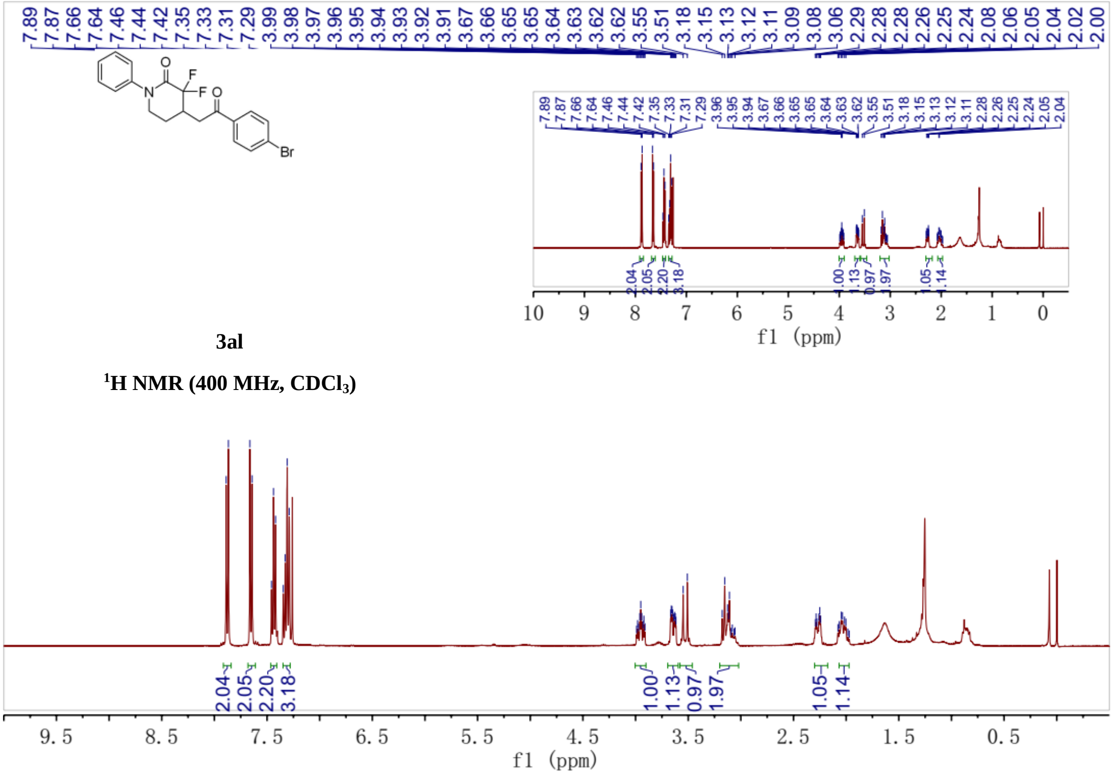

3al

^{1}H NMR (400 MHz, $CDCl_3$)

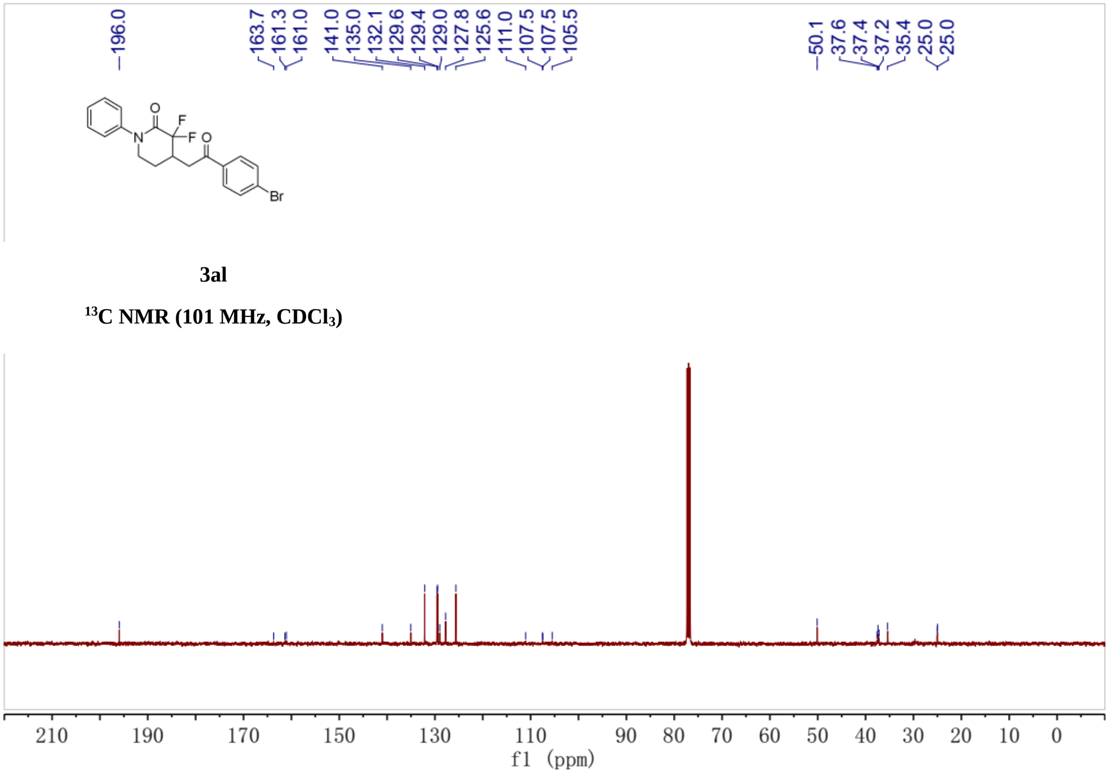

3al

^{13}C NMR (101 MHz, $CDCl_3$)

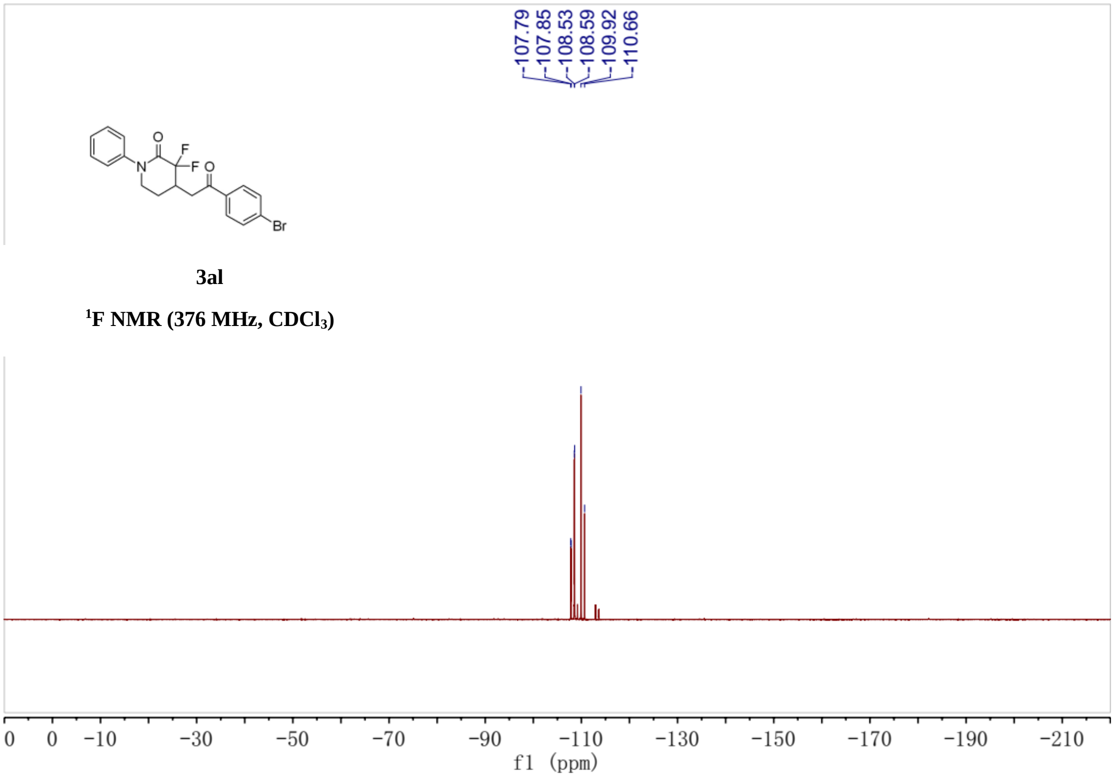

3al

^{1}F NMR (376 MHz, $CDCl_3$)

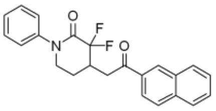

3am

^{1}H NMR (400 MHz, $CDCl_3$)

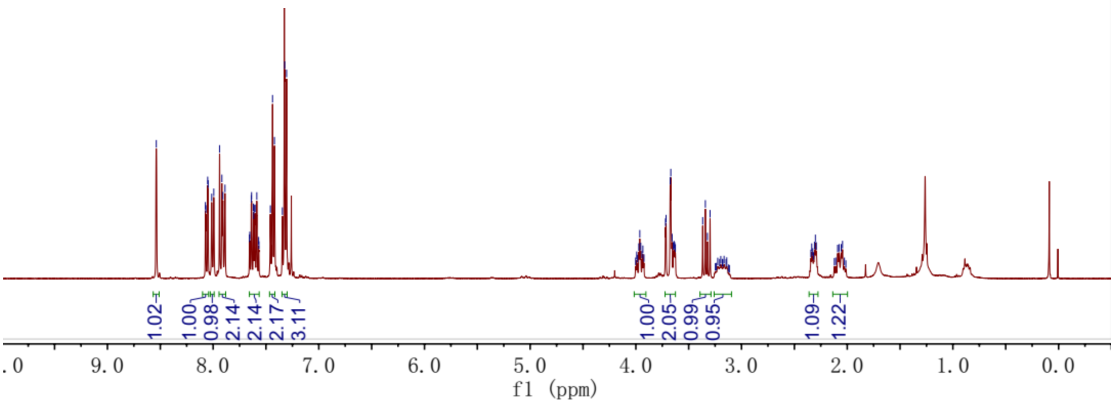

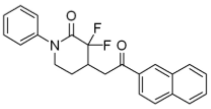

3am

^{13}C NMR (101 MHz, $CDCl_3$)

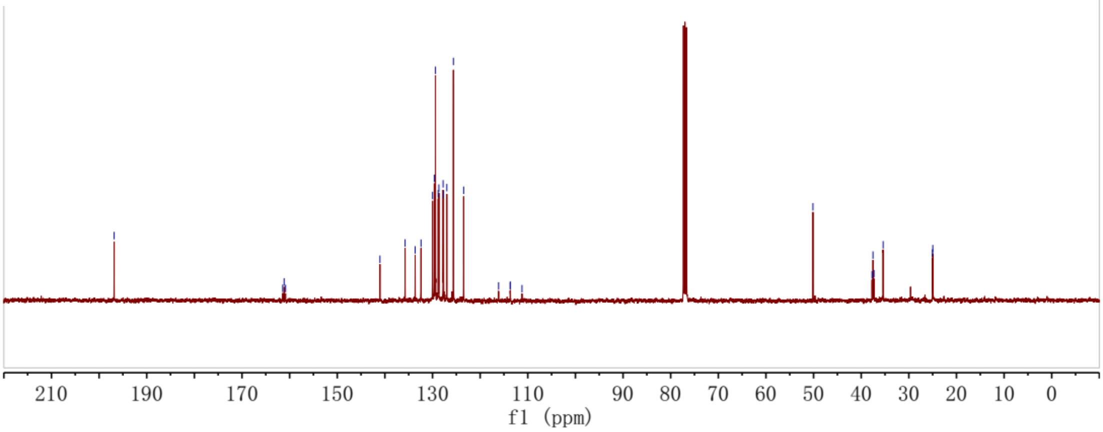

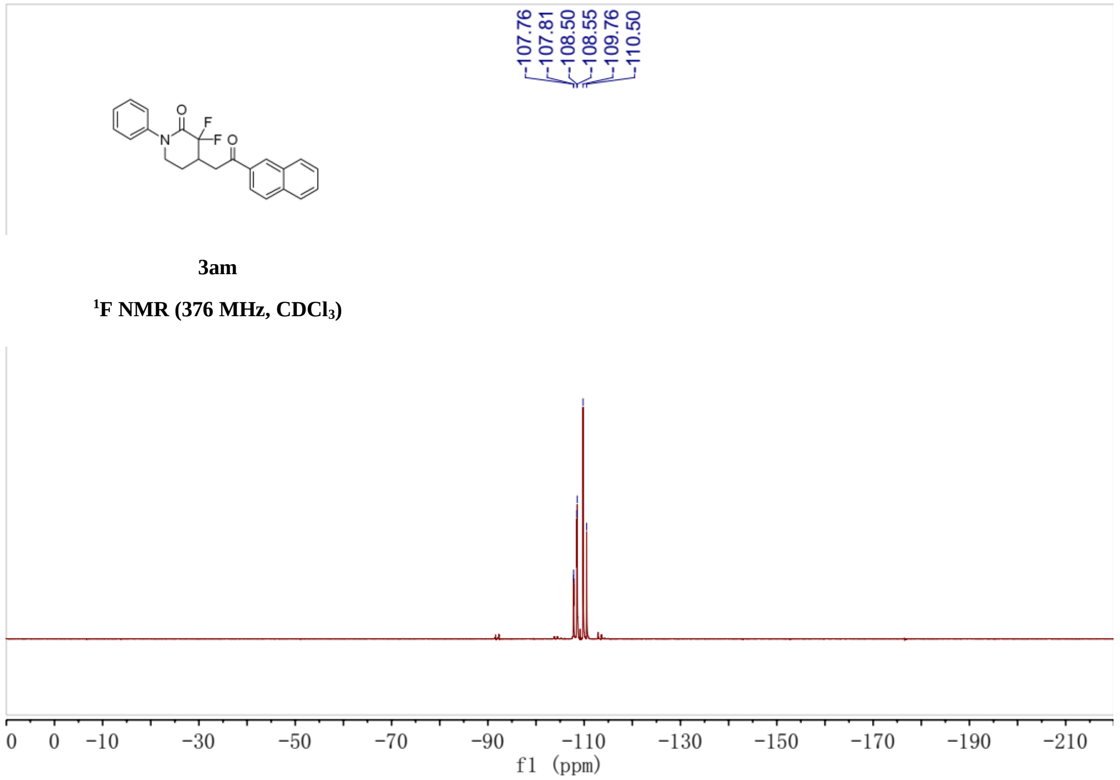

3am

^{1}F NMR (376 MHz, $CDCl_3$)

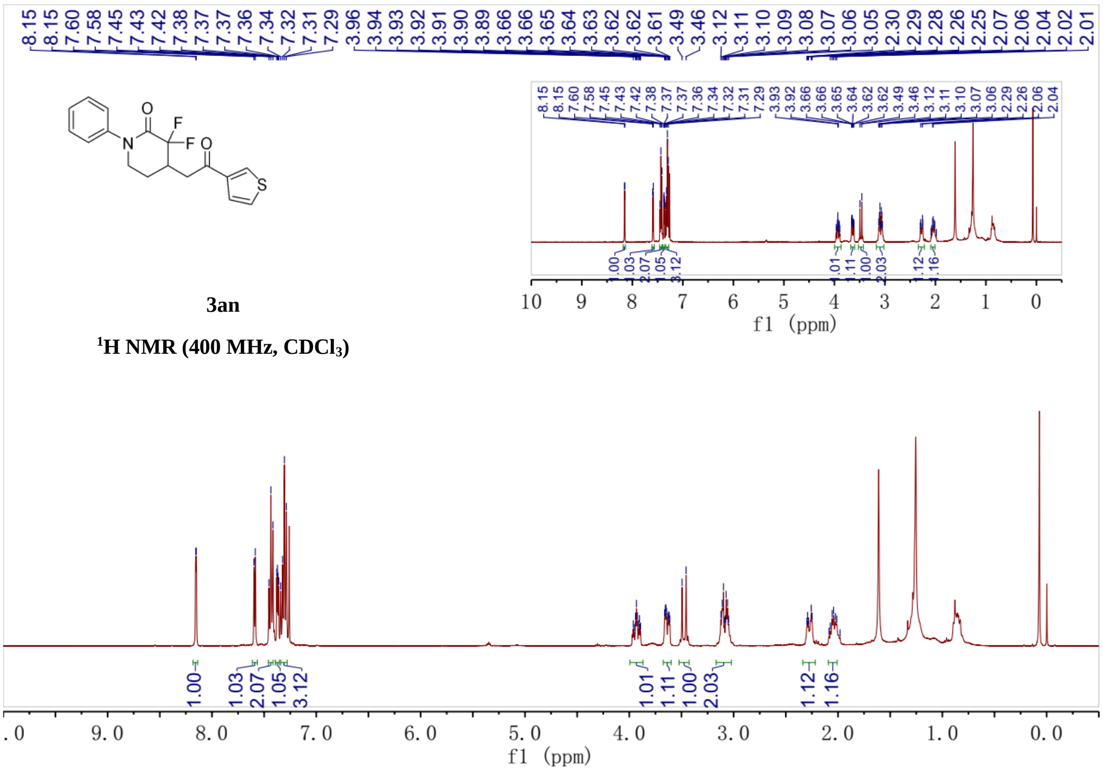

3an

^{1}H NMR (400 MHz, $CDCl_3$)

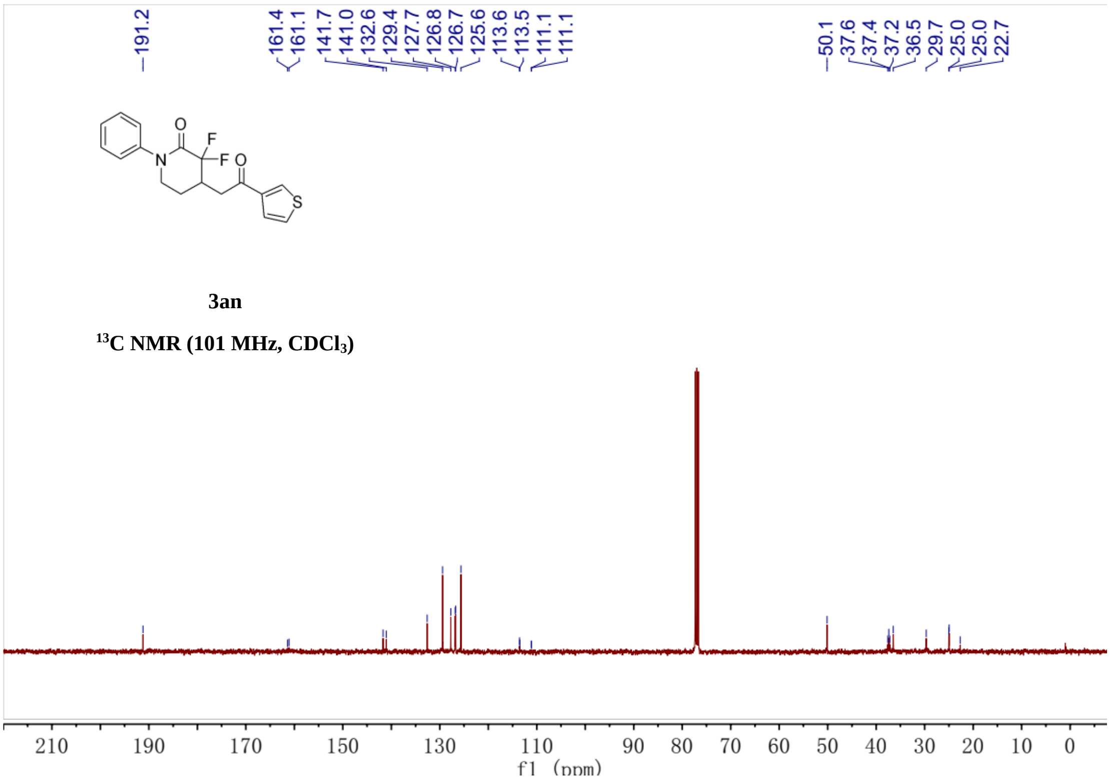

3an

^{13}C NMR (101 MHz, $CDCl_3$)

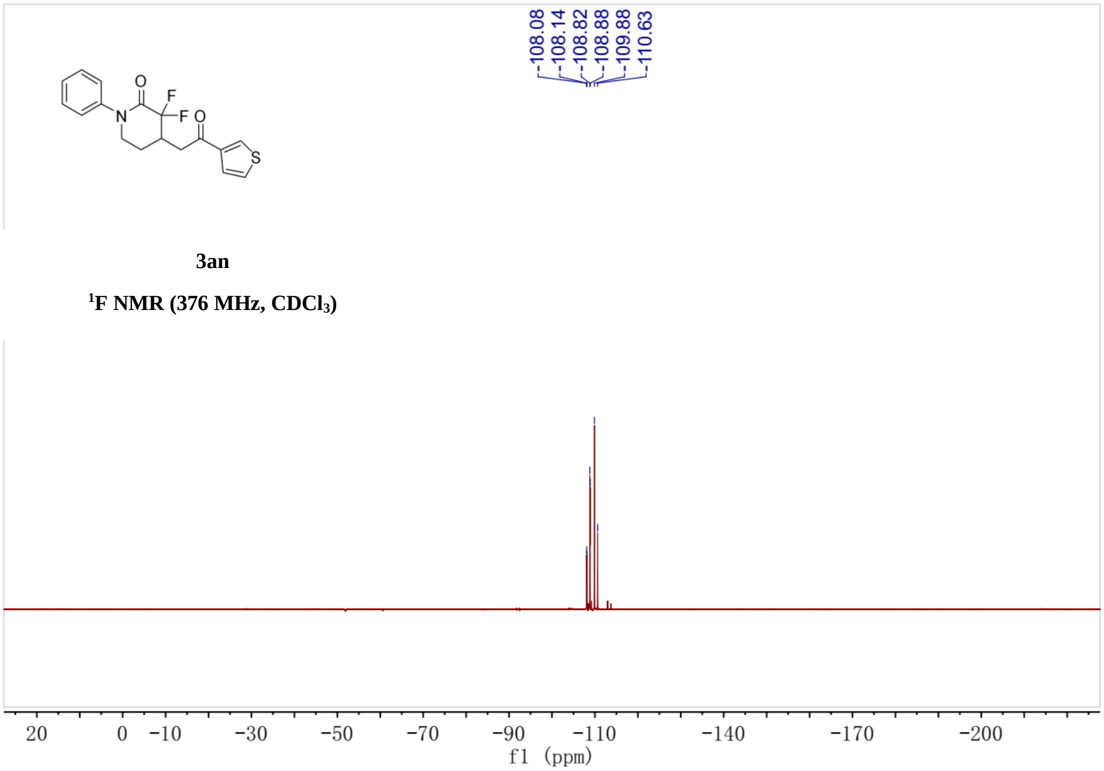

3an

^{1}F NMR (376 MHz, $CDCl_3$)

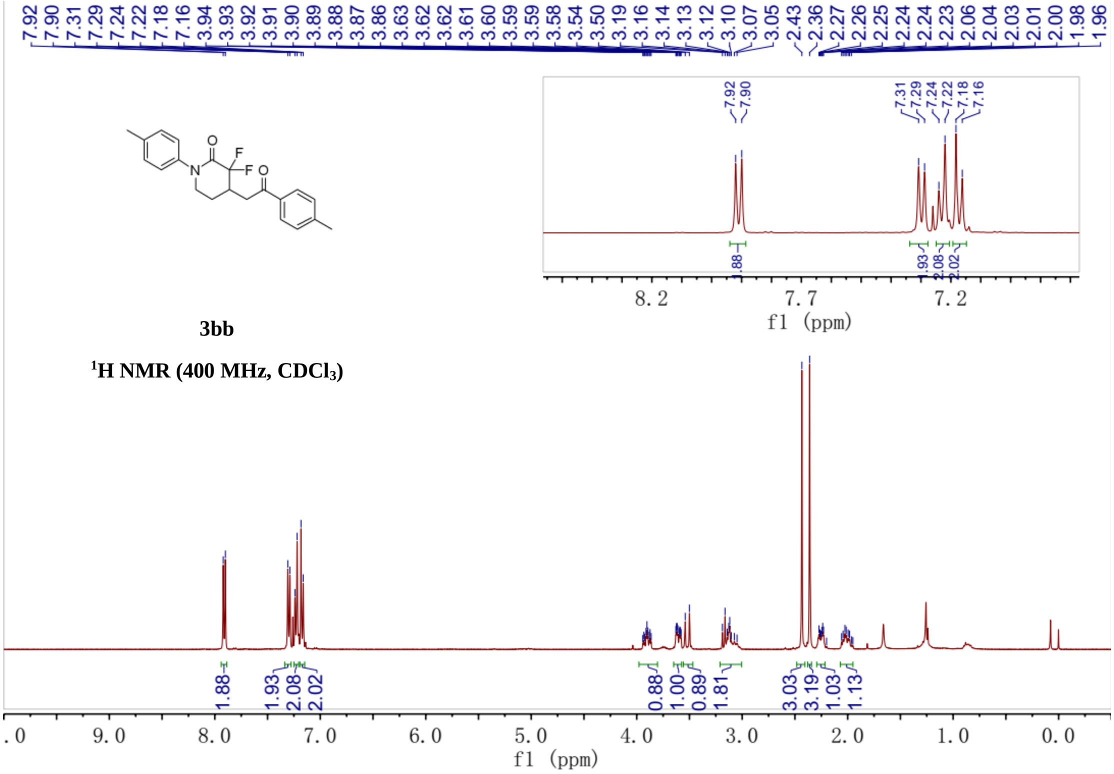

3bb

^{1}H NMR (400 MHz, CDCl$_3$)

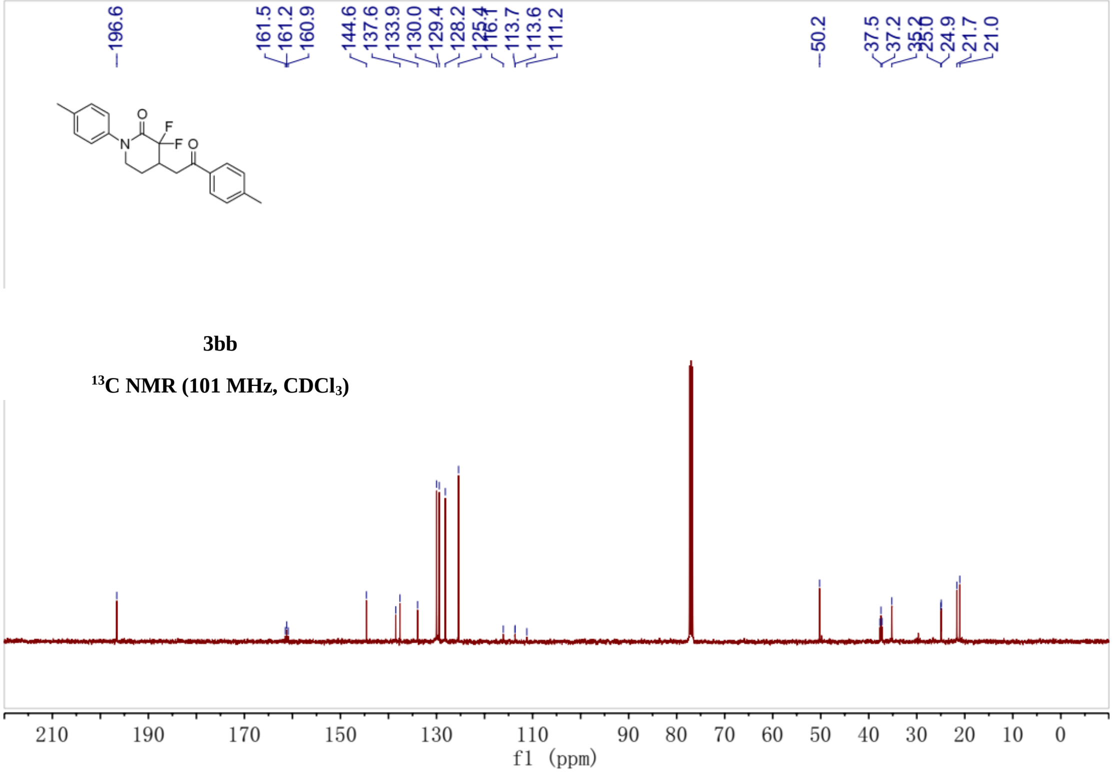

3bb

^{13}C NMR (101 MHz, CDCl$_3$)

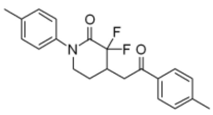

3bb

^{1}F NMR (376 MHz, $CDCl_3$)

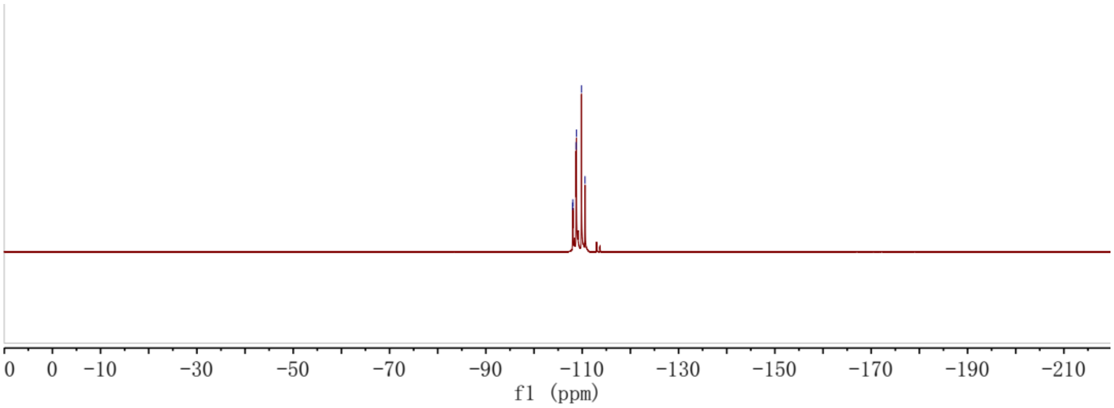

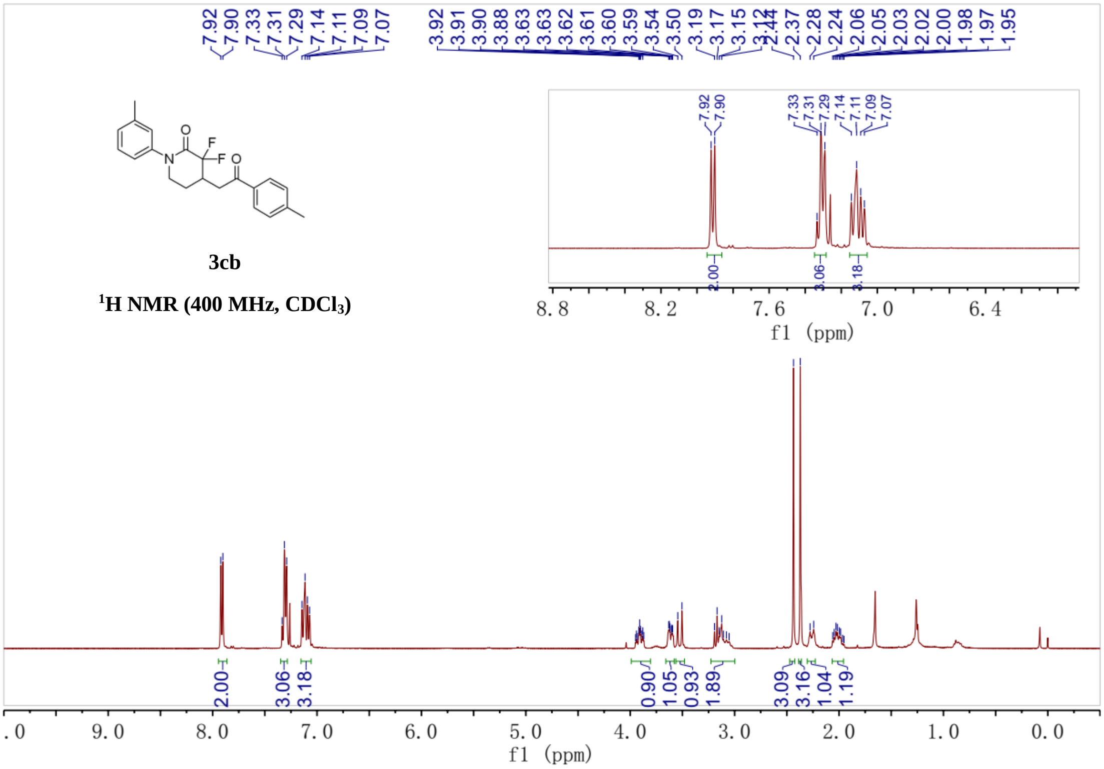
3cb
1H NMR (400 MHz, CDCl3)
f1 (ppm)

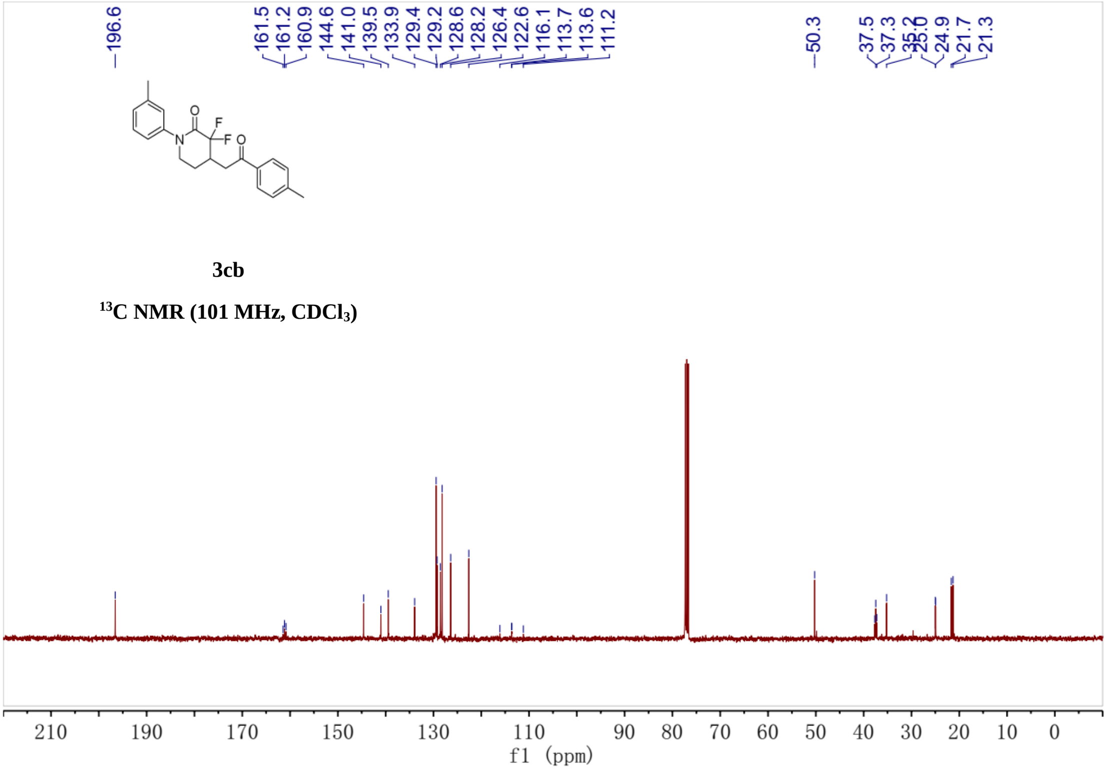
3cb
13C NMR (101 MHz, CDCl3)
f1 (ppm)

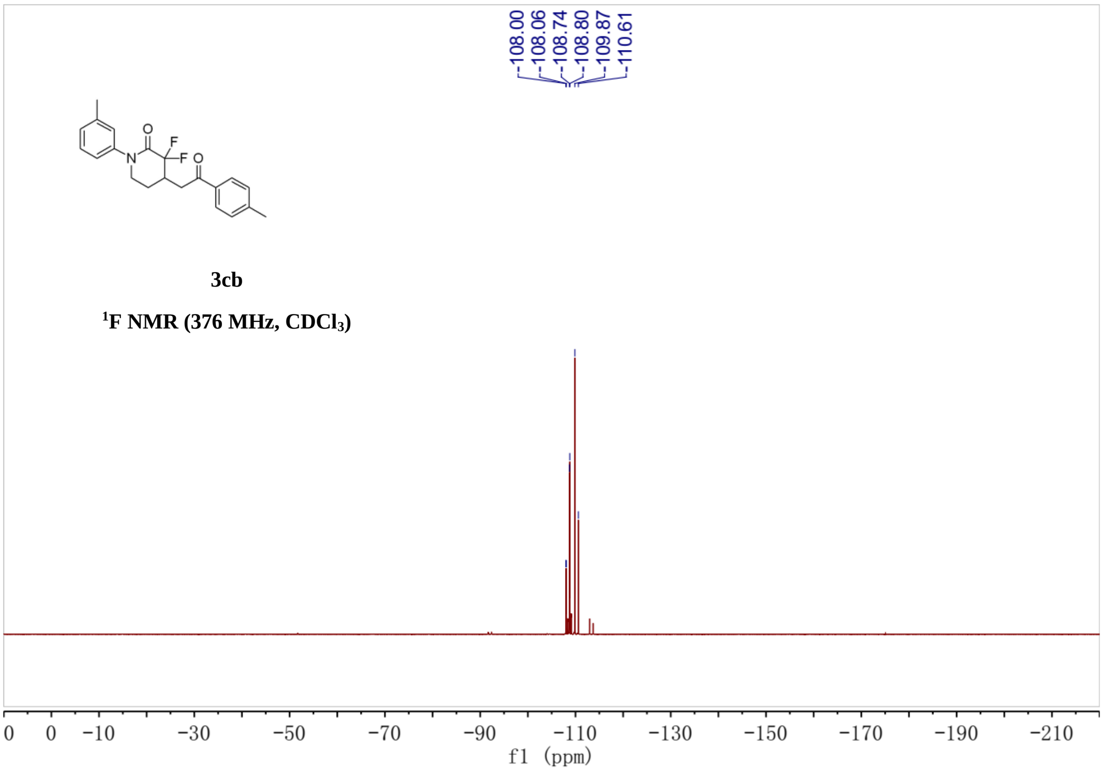

-108.00
-108.06
-108.74
-108.80
-109.87
-110.61
3cb
^{1}F NMR (376 MHz, $CDCl_3$)
0 0 −10 −30 −50 −70 −90 −110 −130 −150 −170 −190 −210
f1 (ppm)

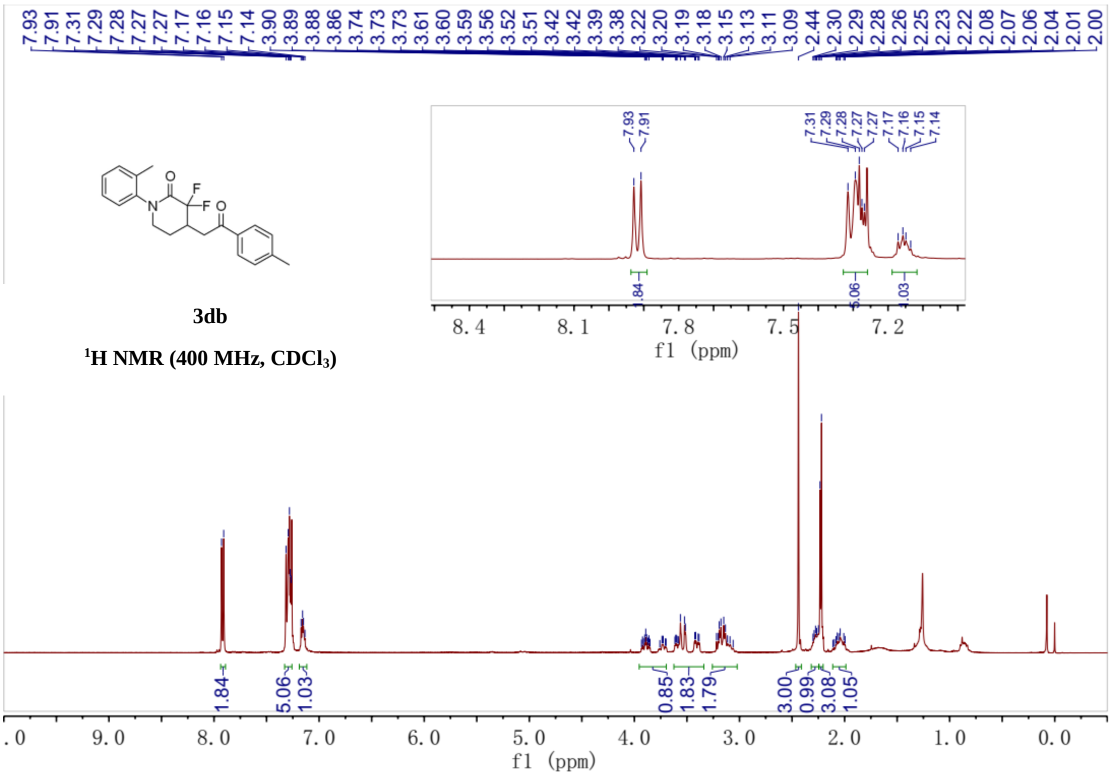

3db

^{1}H NMR (400 MHz, $CDCl_3$)

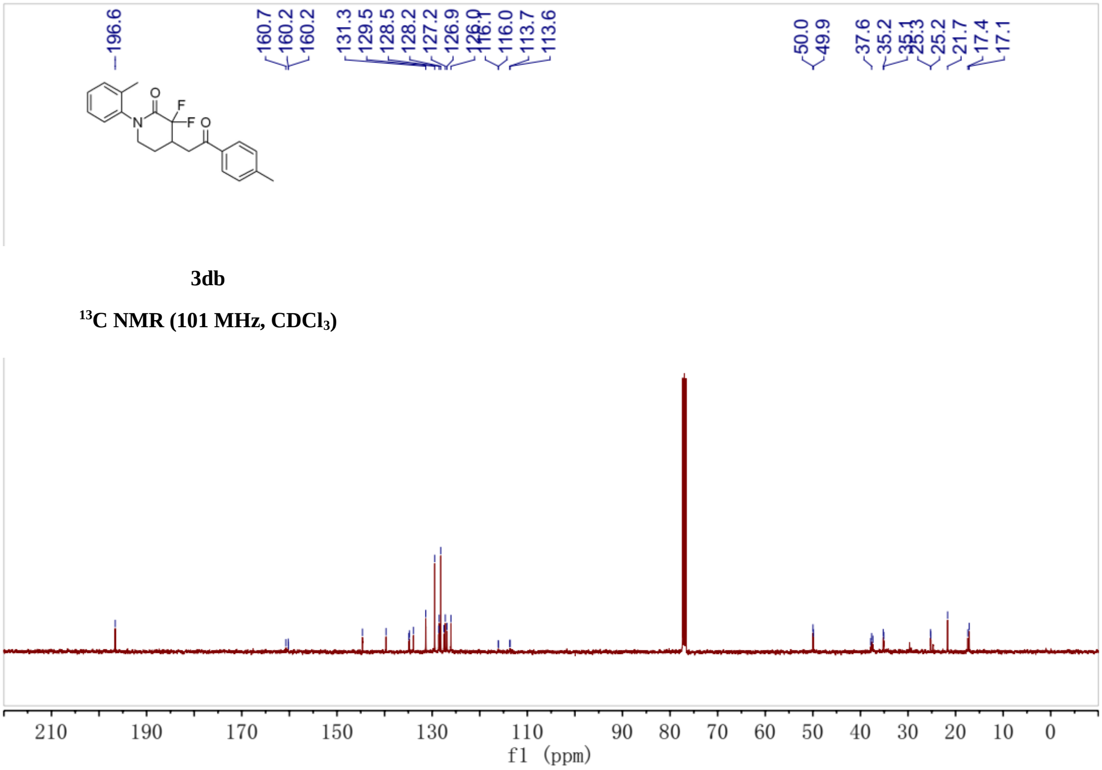

3db

^{13}C NMR (101 MHz, $CDCl_3$)

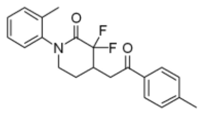

3db

^{1}F NMR (376 MHz, CDCl$_3$)

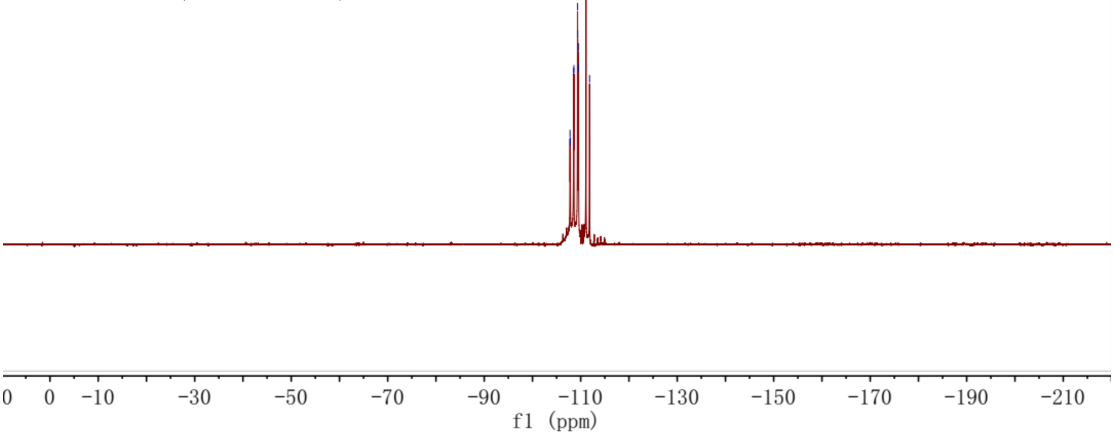

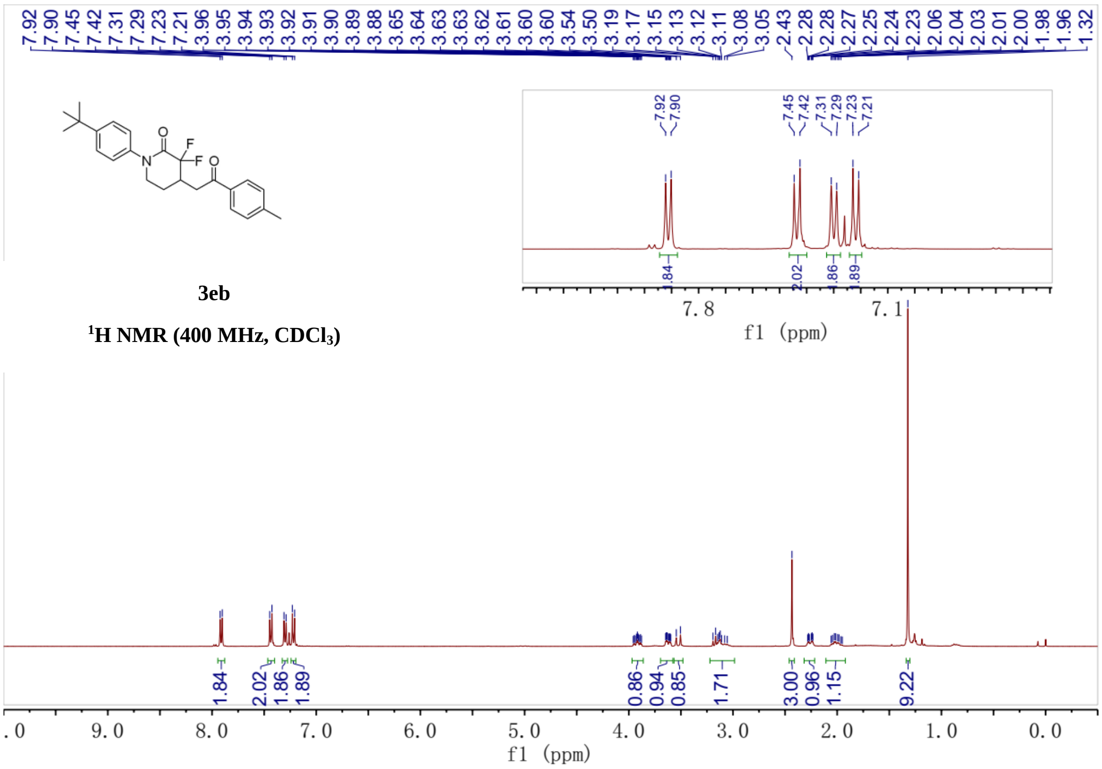

3eb

^{1}H **NMR (400 MHz, $CDCl_3$)**

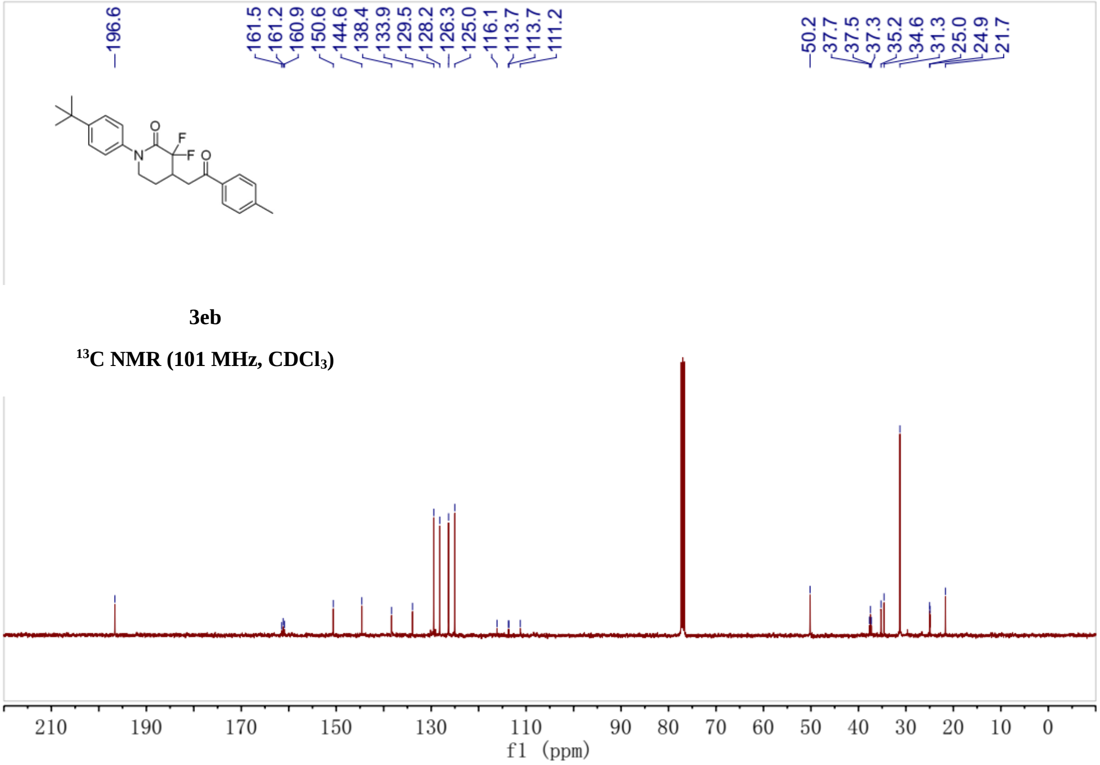

3eb

^{13}C **NMR (101 MHz, $CDCl_3$)**

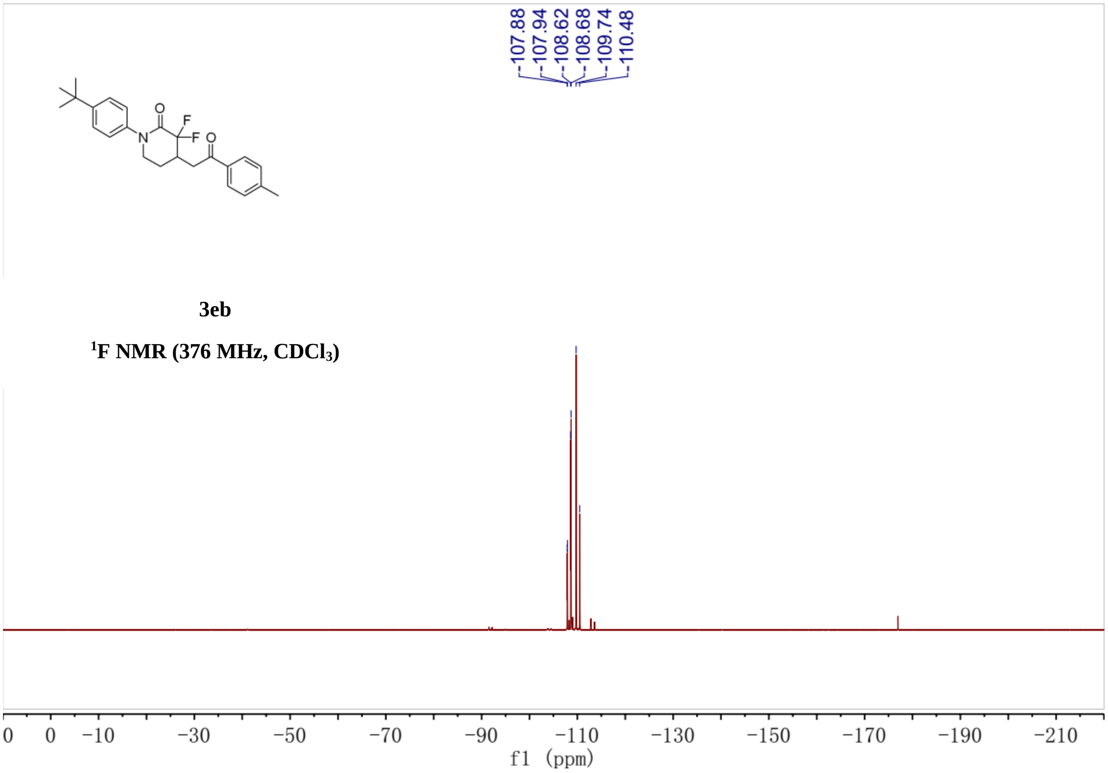

3eb

^{1}F NMR (376 MHz, $CDCl_3$)

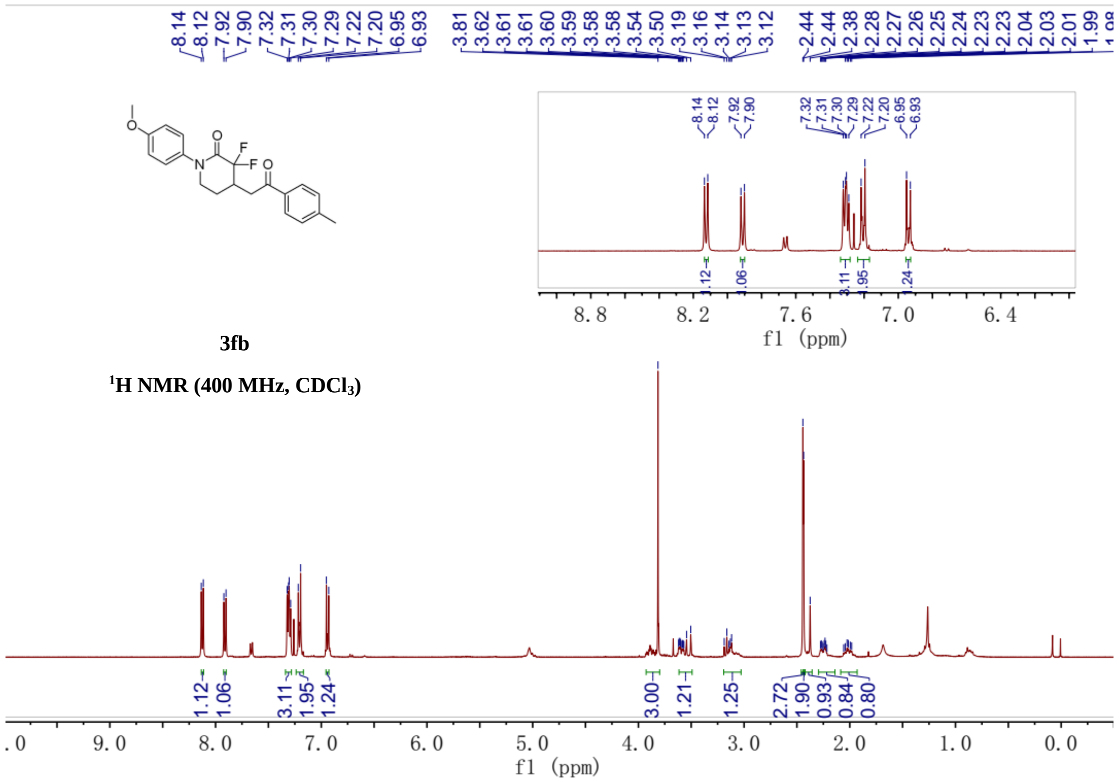

3fb

^{1}H NMR (400 MHz, CDCl$_3$)

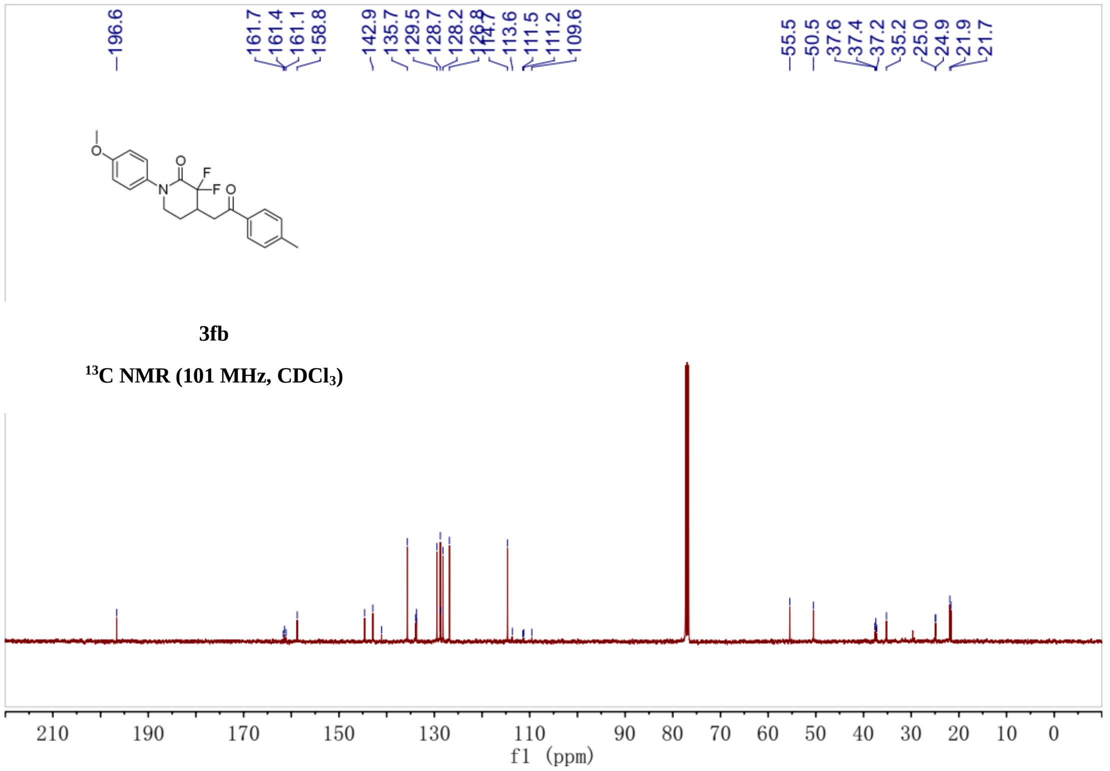

3fb

^{13}C NMR (101 MHz, CDCl$_3$)

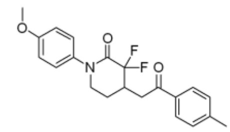

3fb

[1]F NMR (376 MHz, CDCl$_3$)

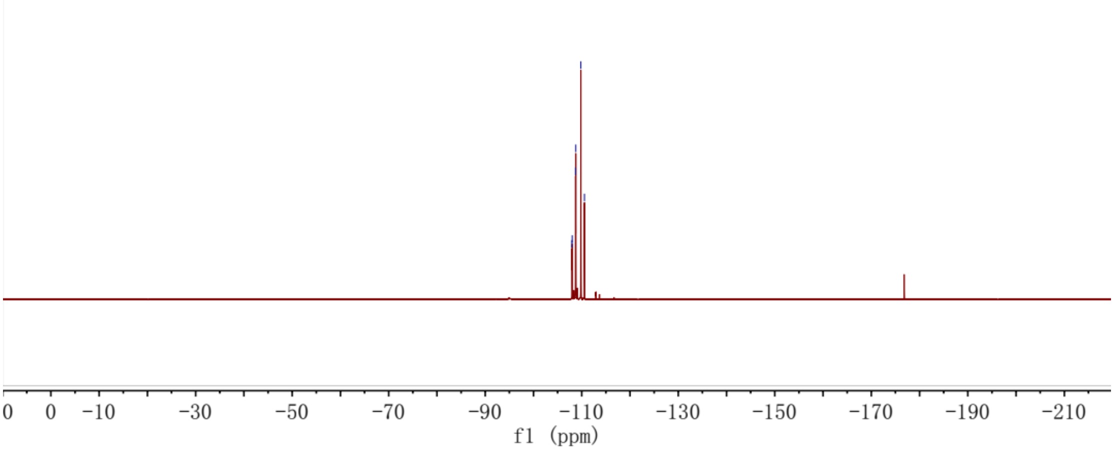

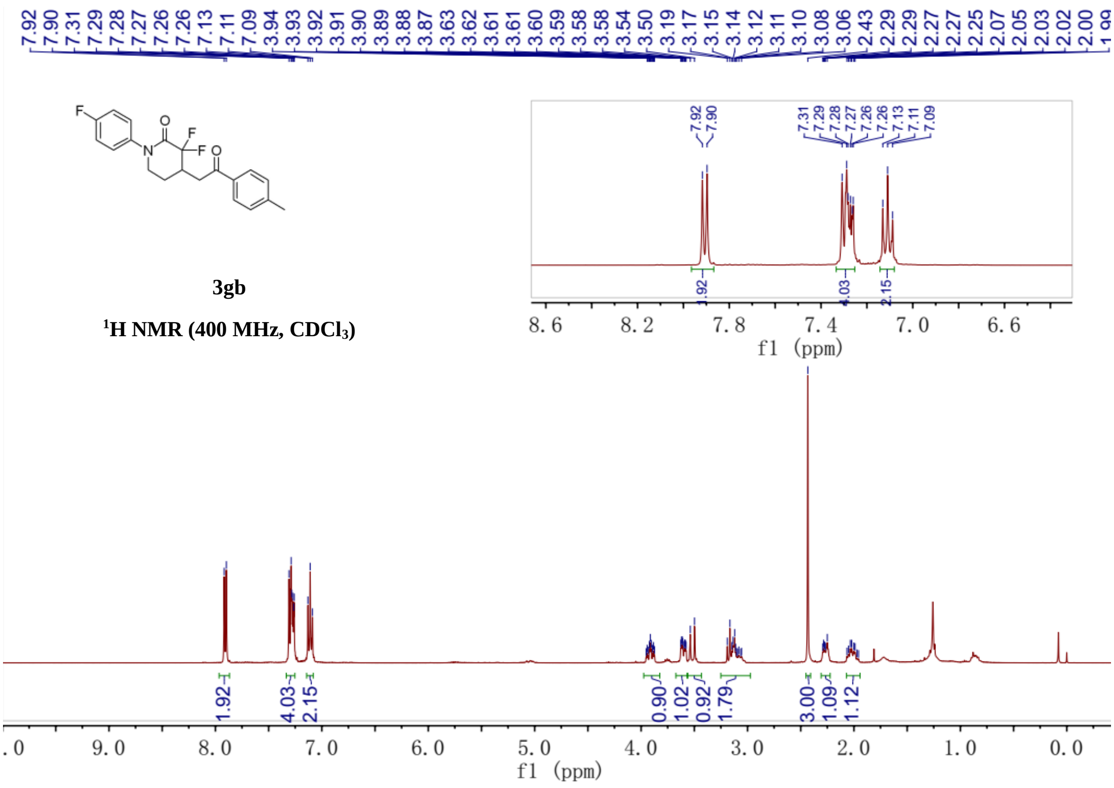

3gb

^{1}H NMR (400 MHz, CDCl$_3$)

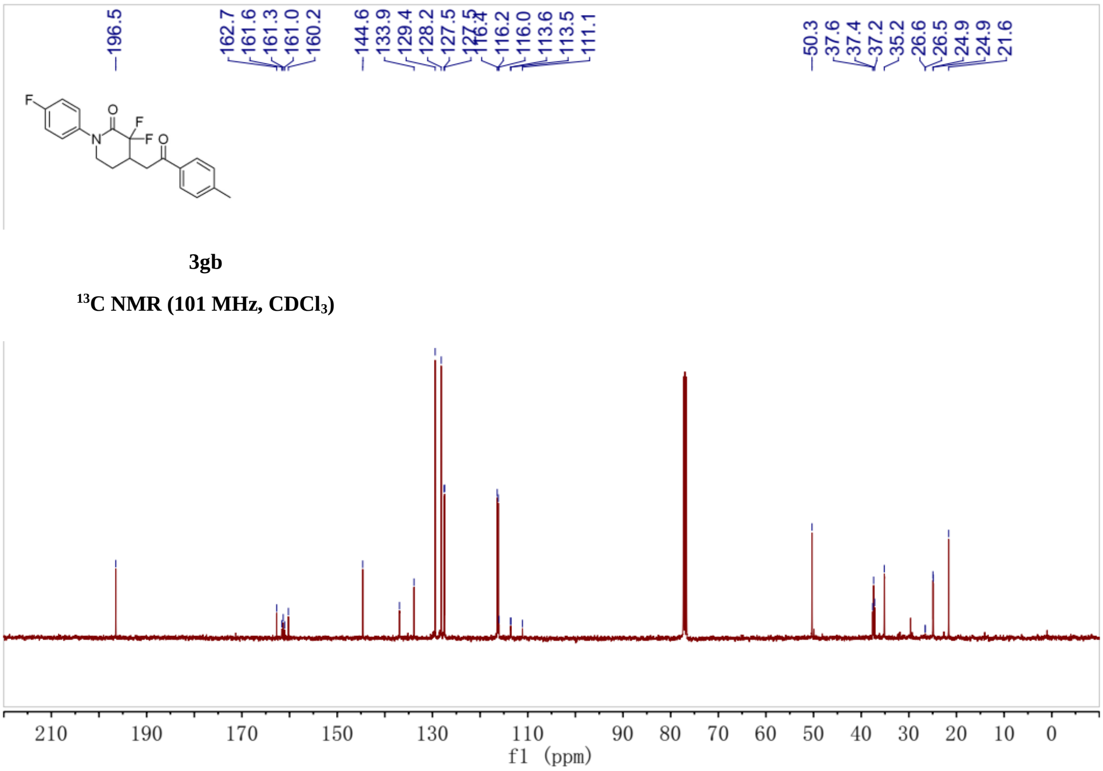

3gb

^{13}C NMR (101 MHz, CDCl$_3$)

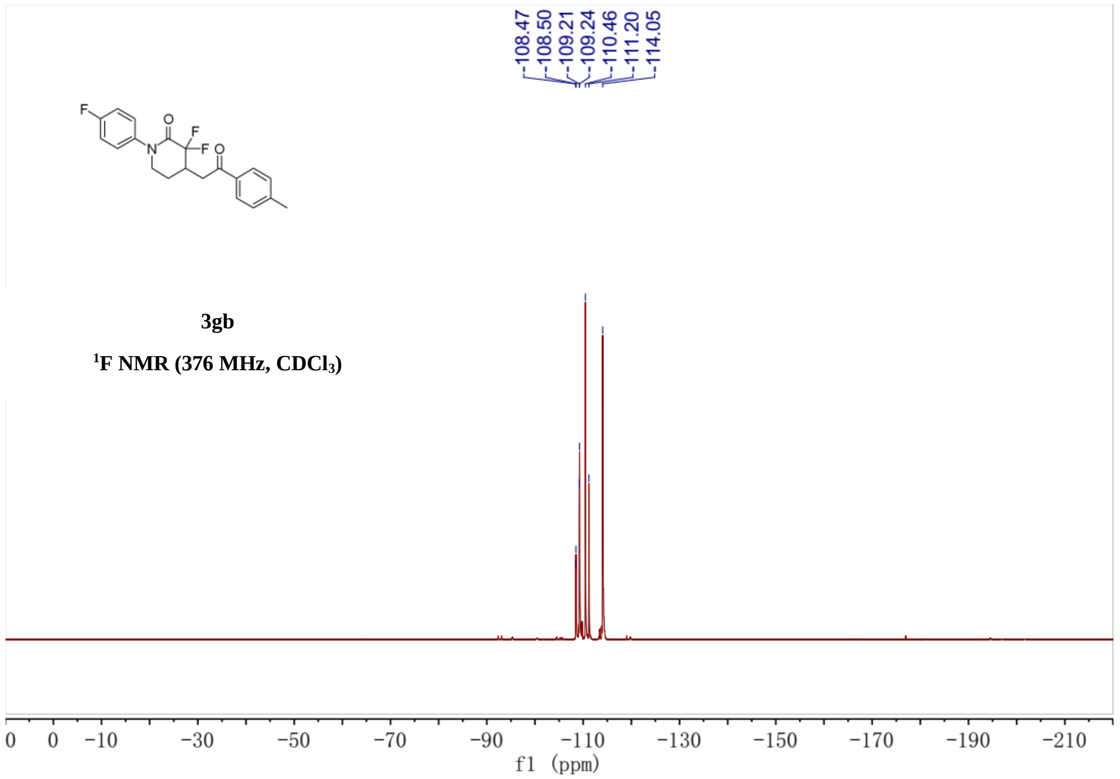
-108.47
-108.50
-109.21
-109.24
-110.46
-111.20
-114.05
3gb
^{1}F NMR (376 MHz, CDCl$_3$)
0 0 −10 −30 −50 −70 −90 −110 −130 −150 −170 −190 −210
f1 (ppm)

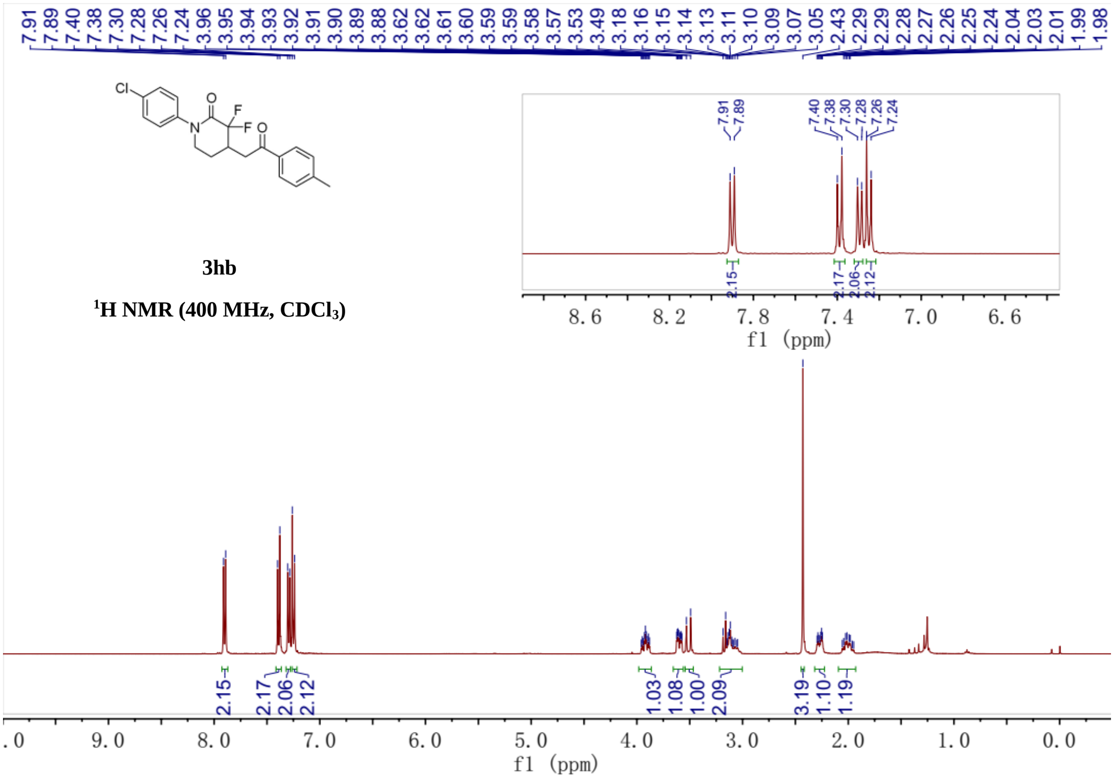
3hb
1H NMR (400 MHz, CDCl3)
f1 (ppm)

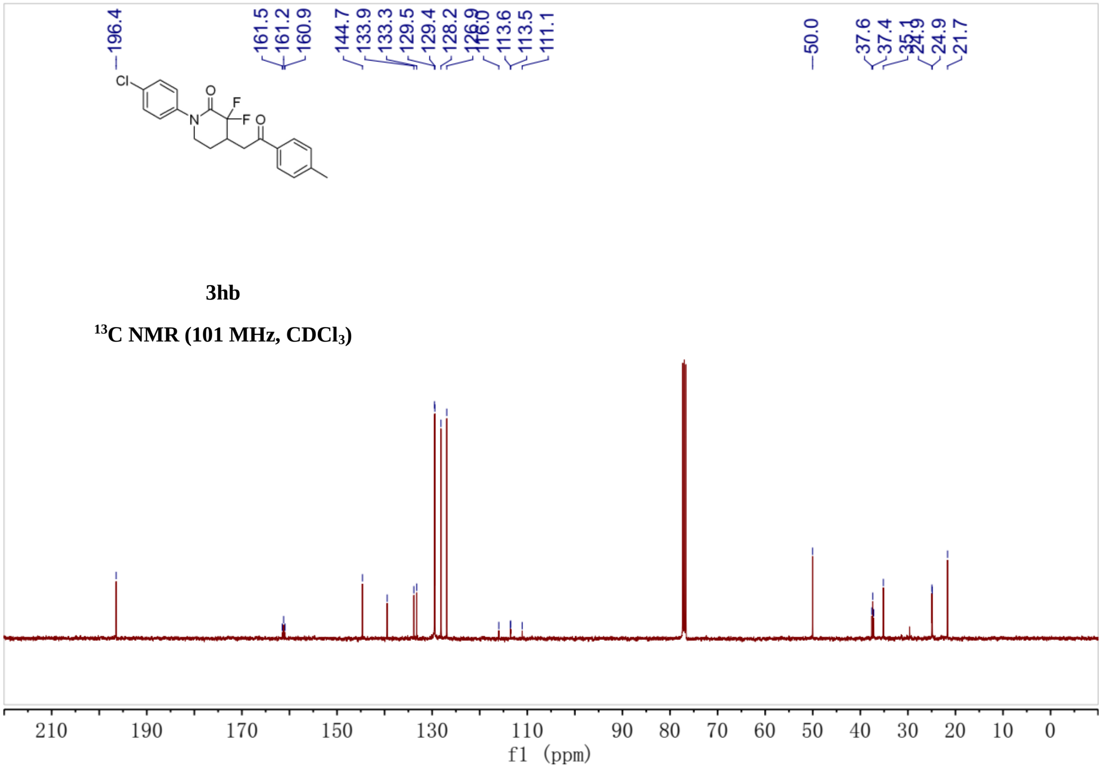
3hb
13C NMR (101 MHz, CDCl3)
f1 (ppm)

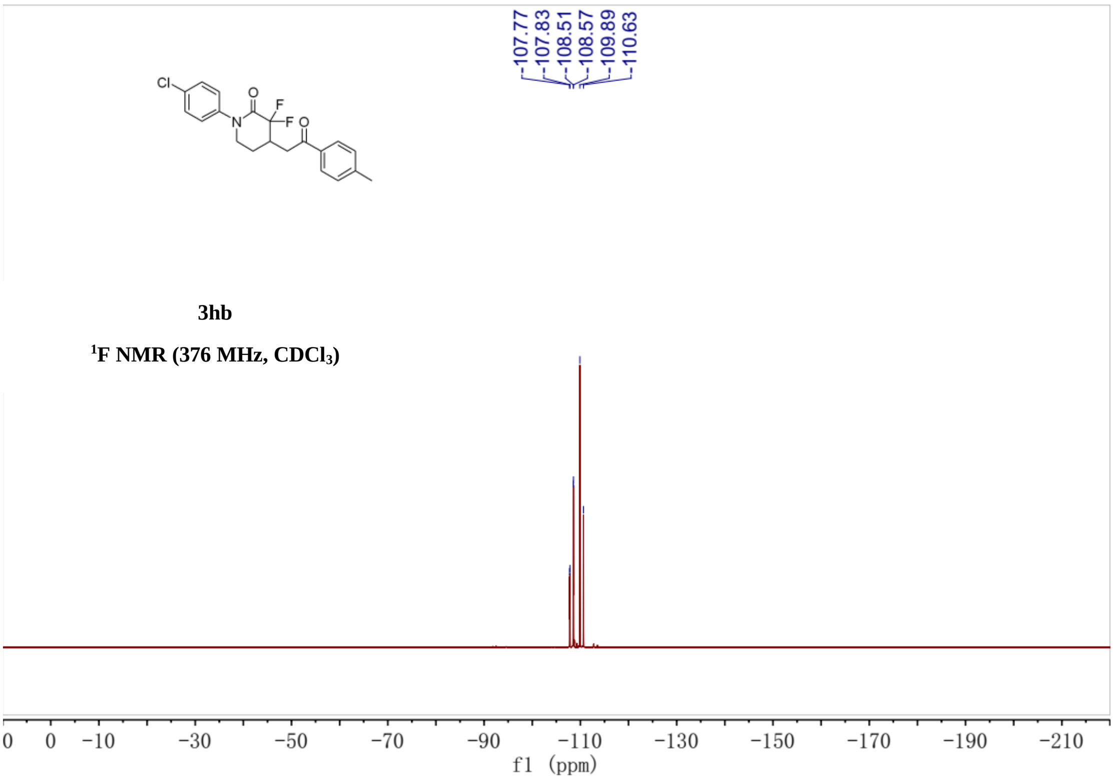
107.77
107.83
108.51
108.57
109.89
110.63
3hb
1F NMR (376 MHz, CDCl3)
0
0
−10
−30
−50
−70
−90
−110
−130
−150
−170
−190
−210
f1 (ppm)

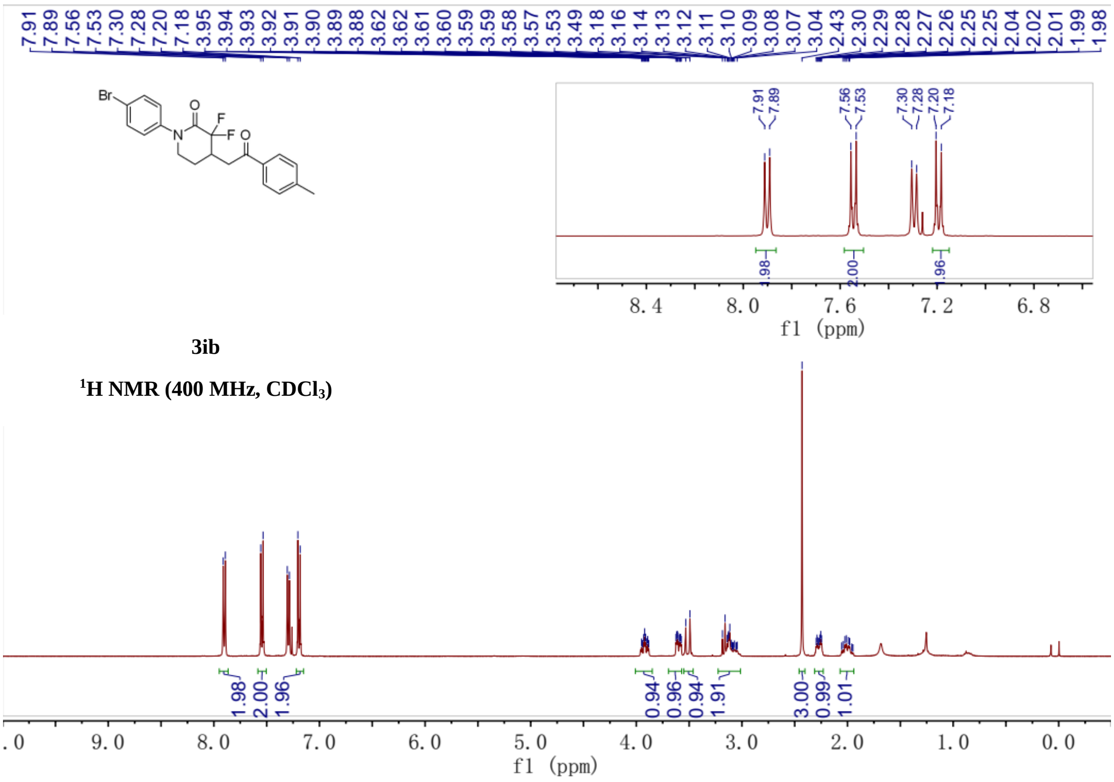

3ib

^{1}H NMR (400 MHz, CDCl$_3$)

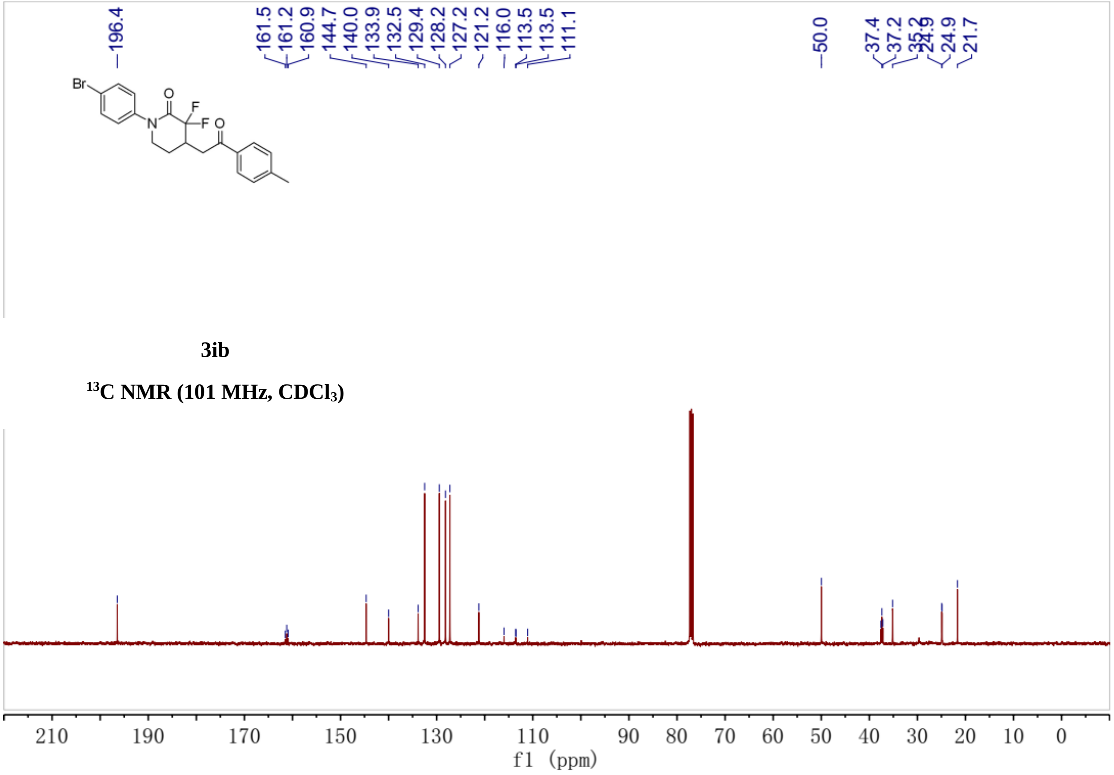

3ib

^{13}C NMR (101 MHz, CDCl$_3$)

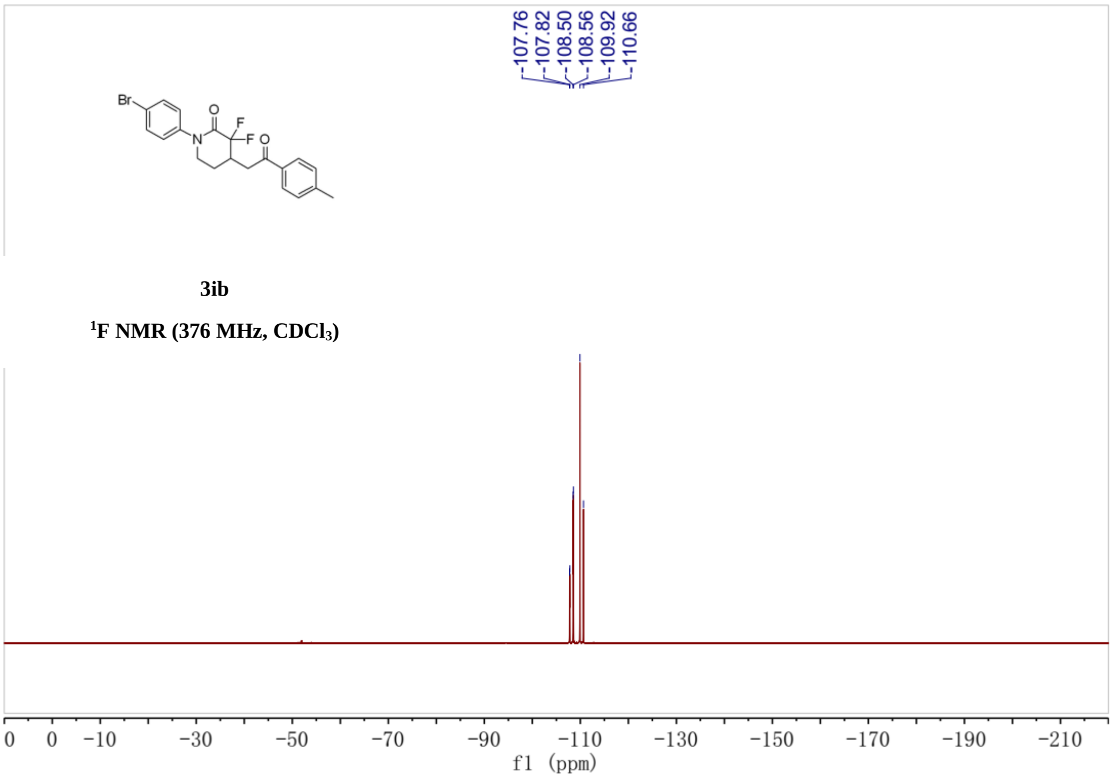
-107.76
-107.82
-108.50
-108.56
-109.92
-110.66
3ib
1F NMR (376 MHz, CDCl3)
0 0 −10 −30 −50 −70 −90 −110 −130 −150 −170 −190 −210
f1 (ppm)

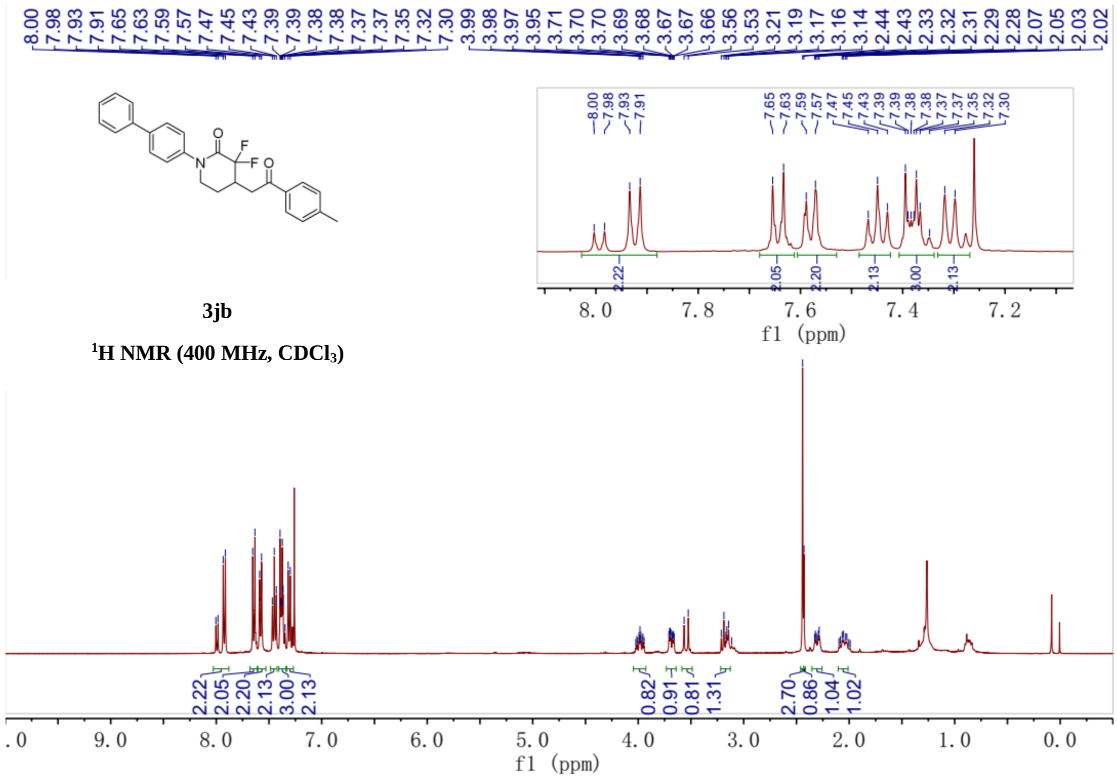

3jb

^{1}H NMR (400 MHz, CDCl$_3$)

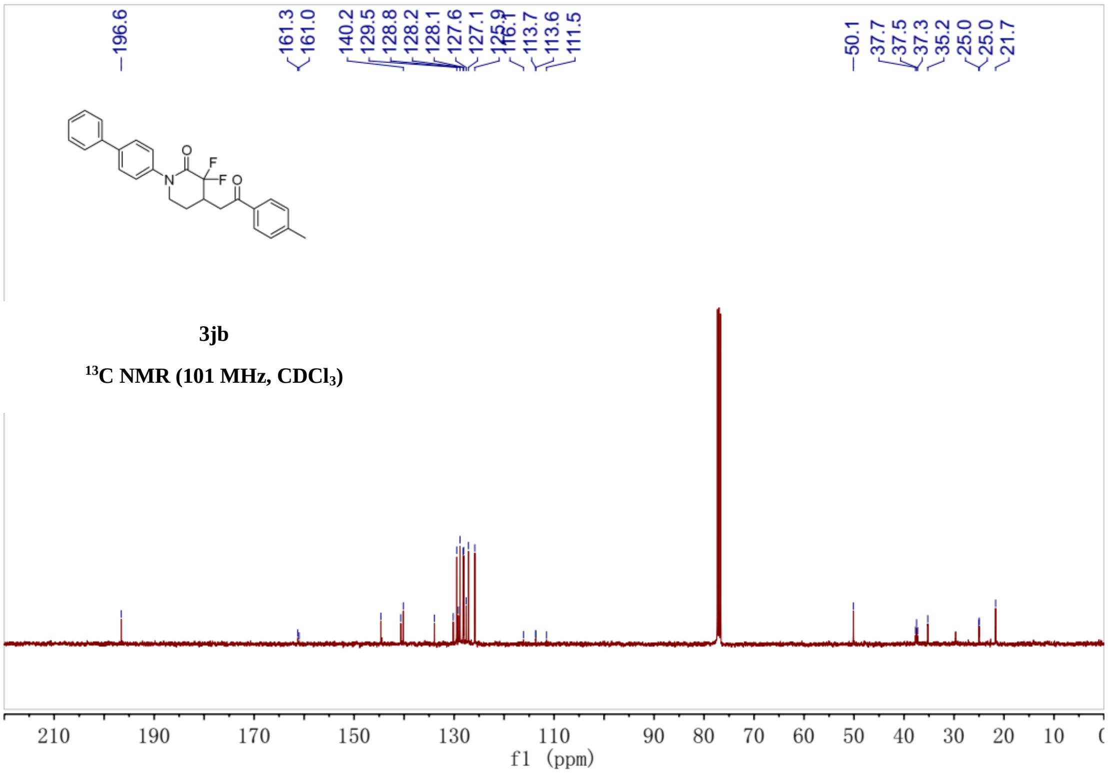

3jb

^{13}C NMR (101 MHz, CDCl$_3$)

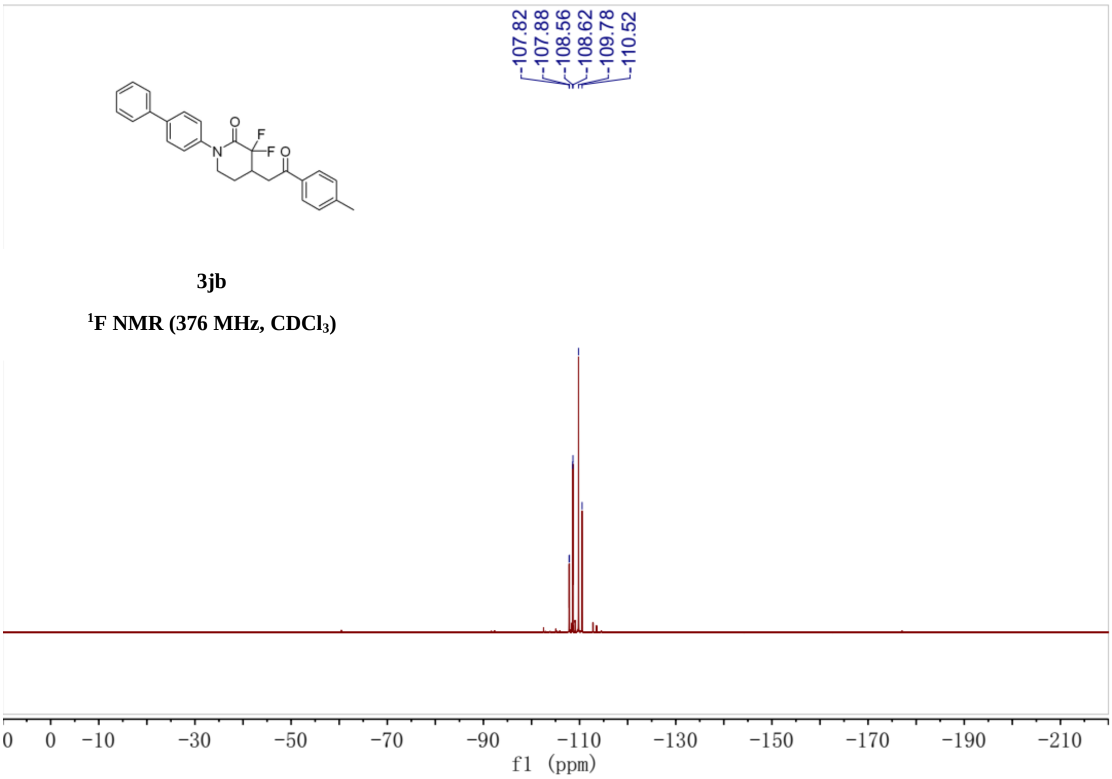

3jb

[1]F NMR (376 MHz, CDCl$_3$)

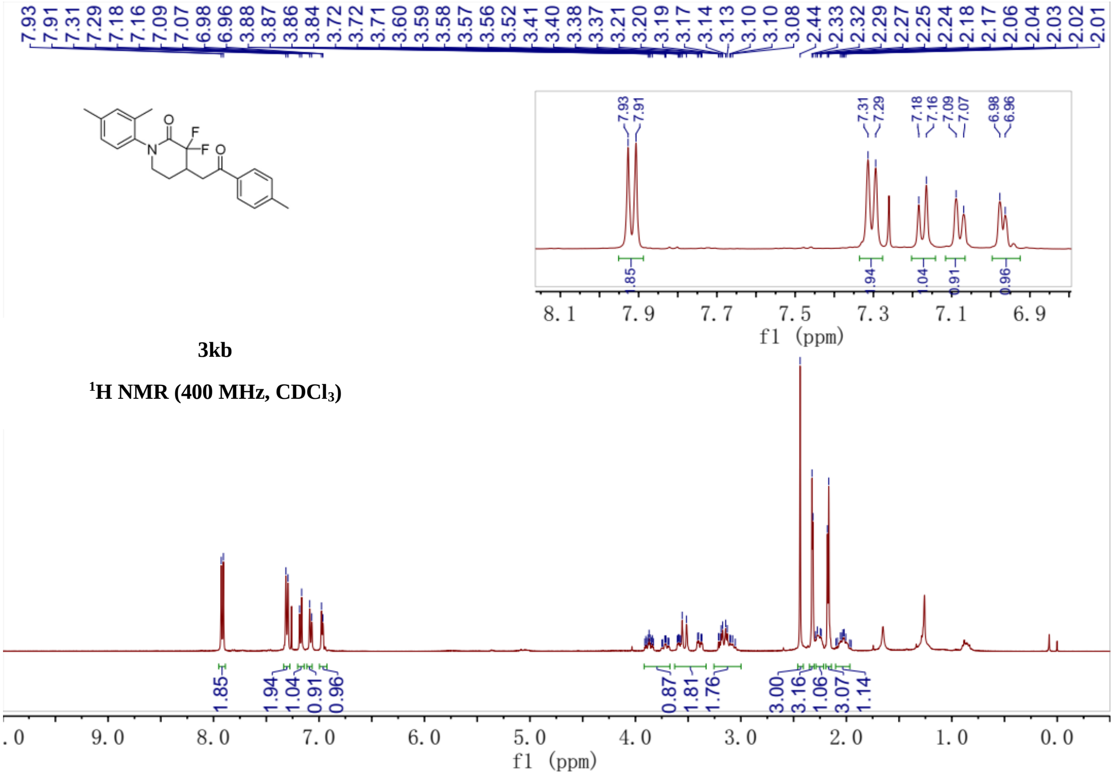

3kb

^{1}H NMR (400 MHz, $CDCl_3$)

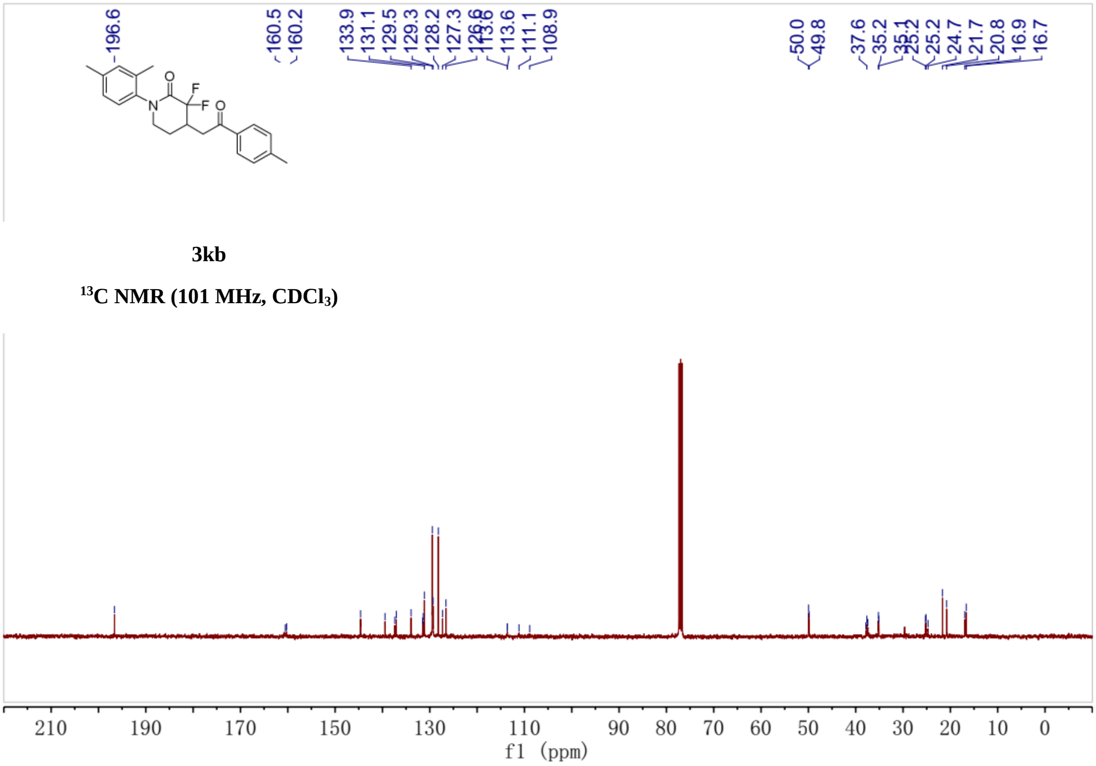

3kb

^{13}C NMR (101 MHz, $CDCl_3$)

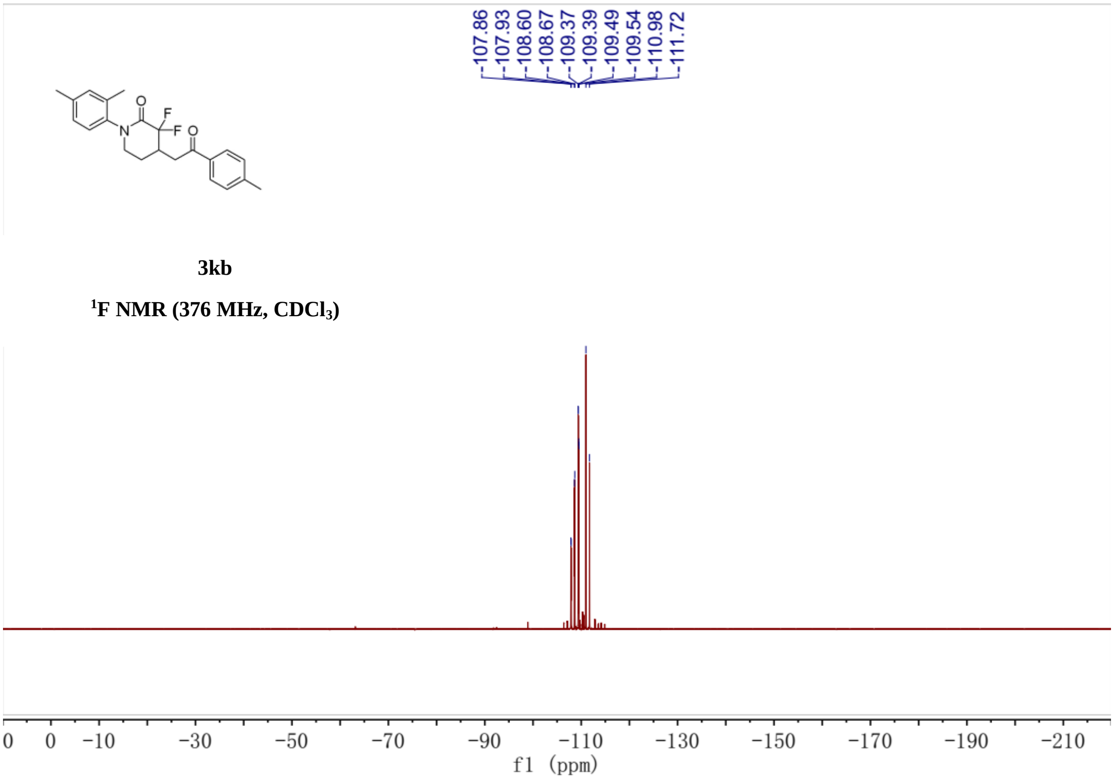

3kb

^{1}F NMR (376 MHz, $CDCl_3$)

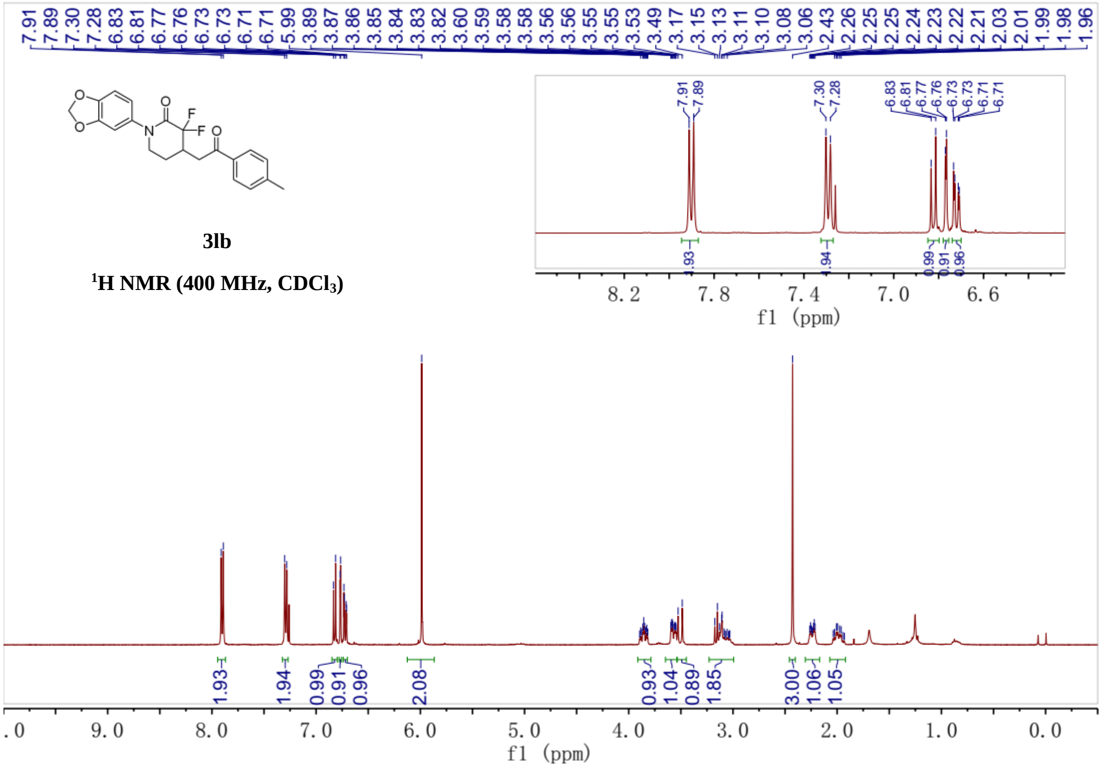

3lb

^{1}H NMR (400 MHz, $CDCl_3$)

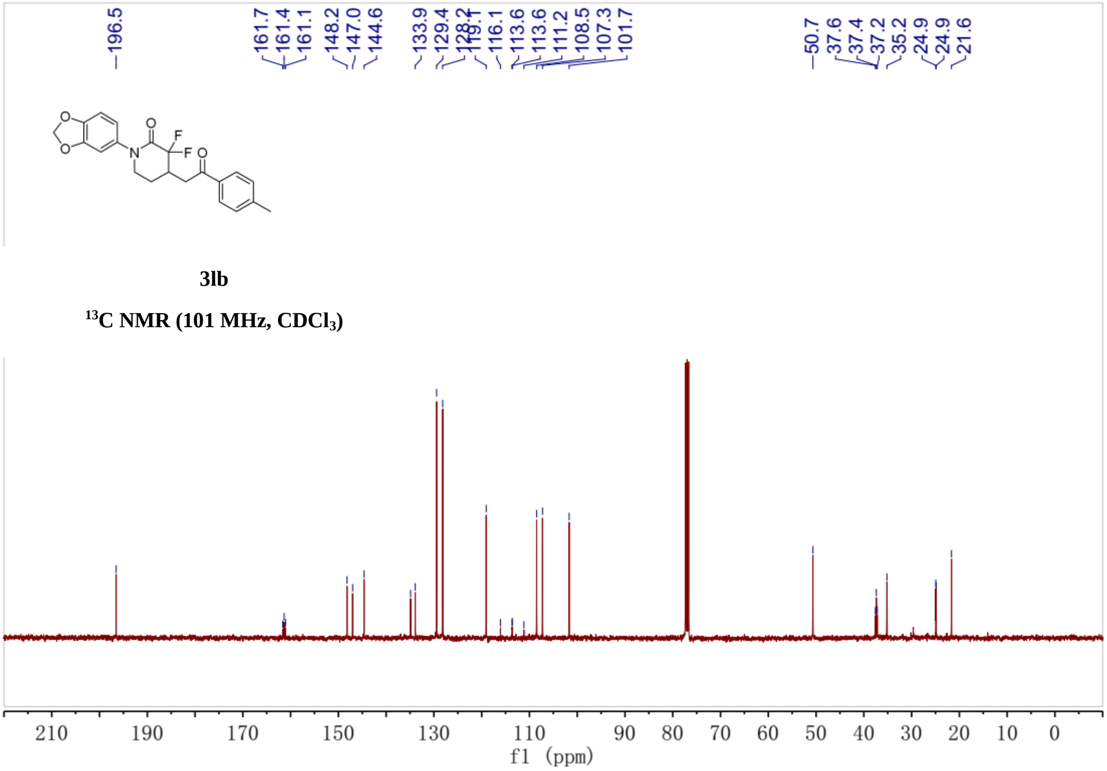

3lb

^{13}C NMR (101 MHz, $CDCl_3$)

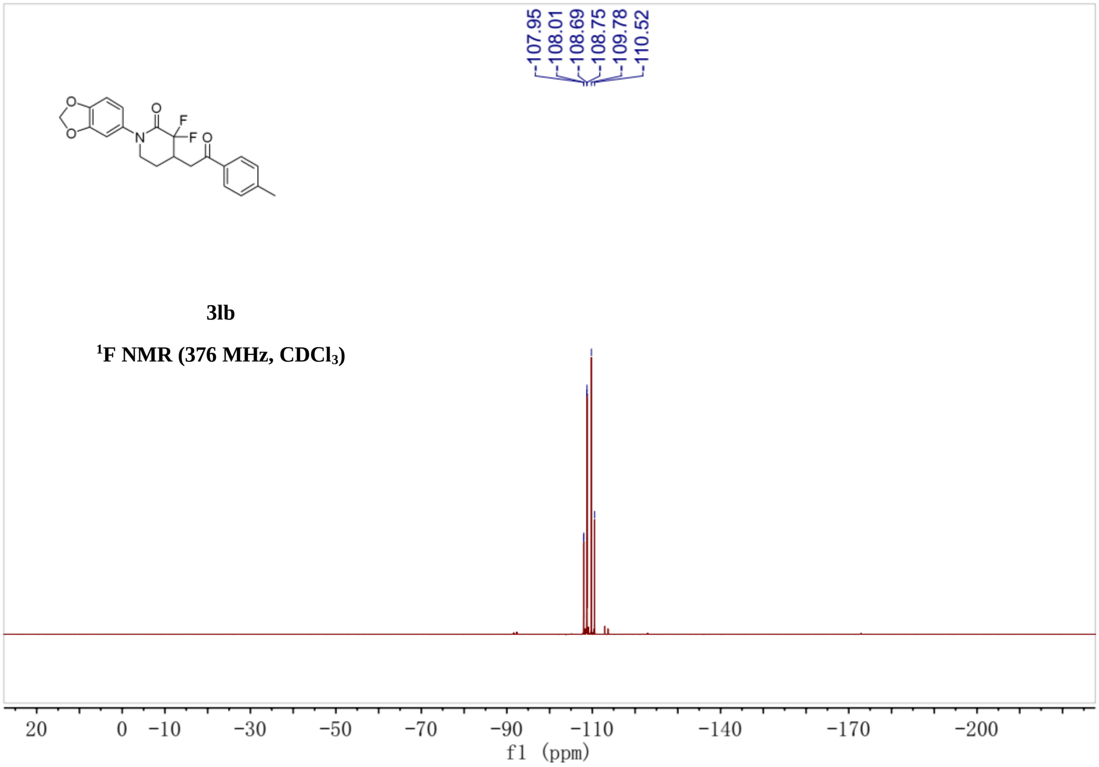

-107.95
-108.01
-108.69
-108.75
-109.78
-110.52
3lb
^{1}F NMR (376 MHz, $CDCl_3$)
20 0 −10 −30 −50 −70 −90 −110 −140 −170 −200
f1 (ppm)

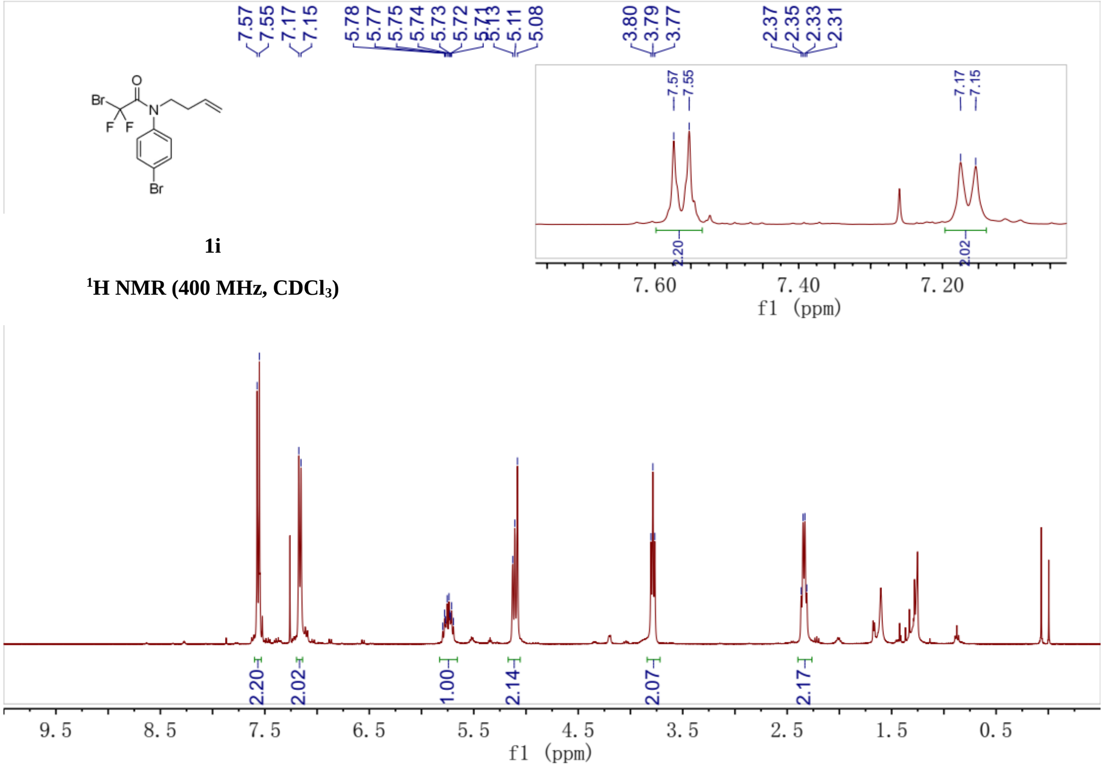

1i

^{1}H NMR (400 MHz, $CDCl_3$)

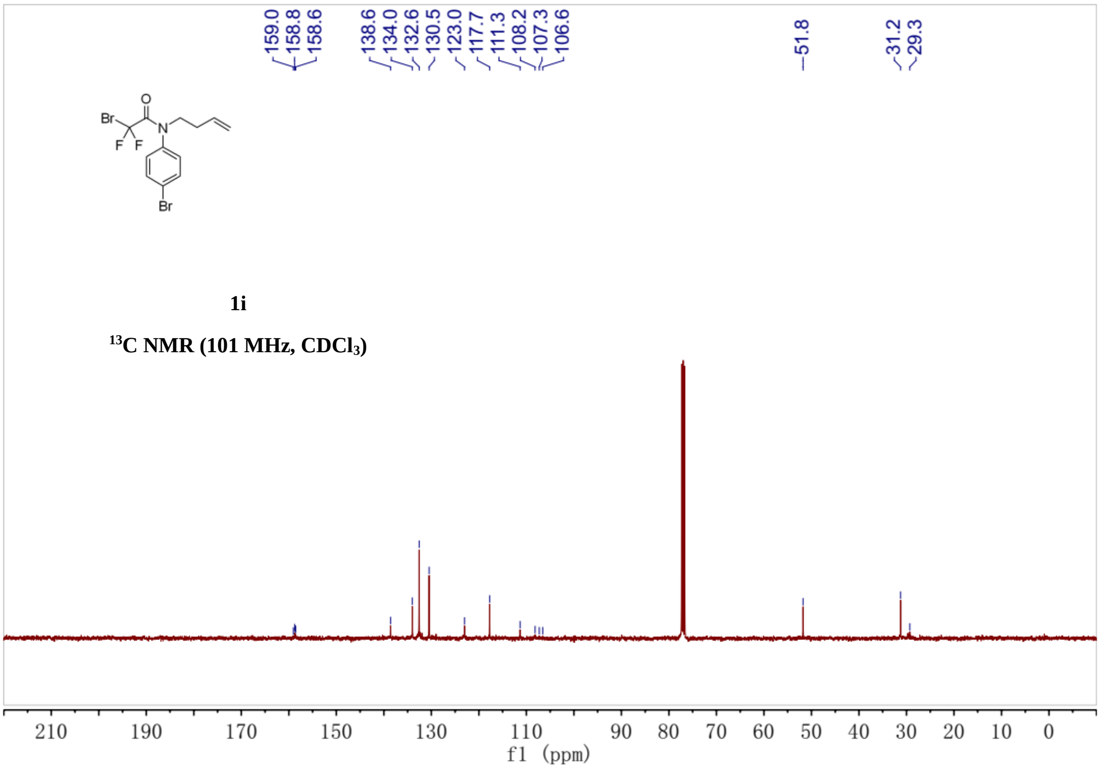

1i

^{13}C NMR (101 MHz, $CDCl_3$)

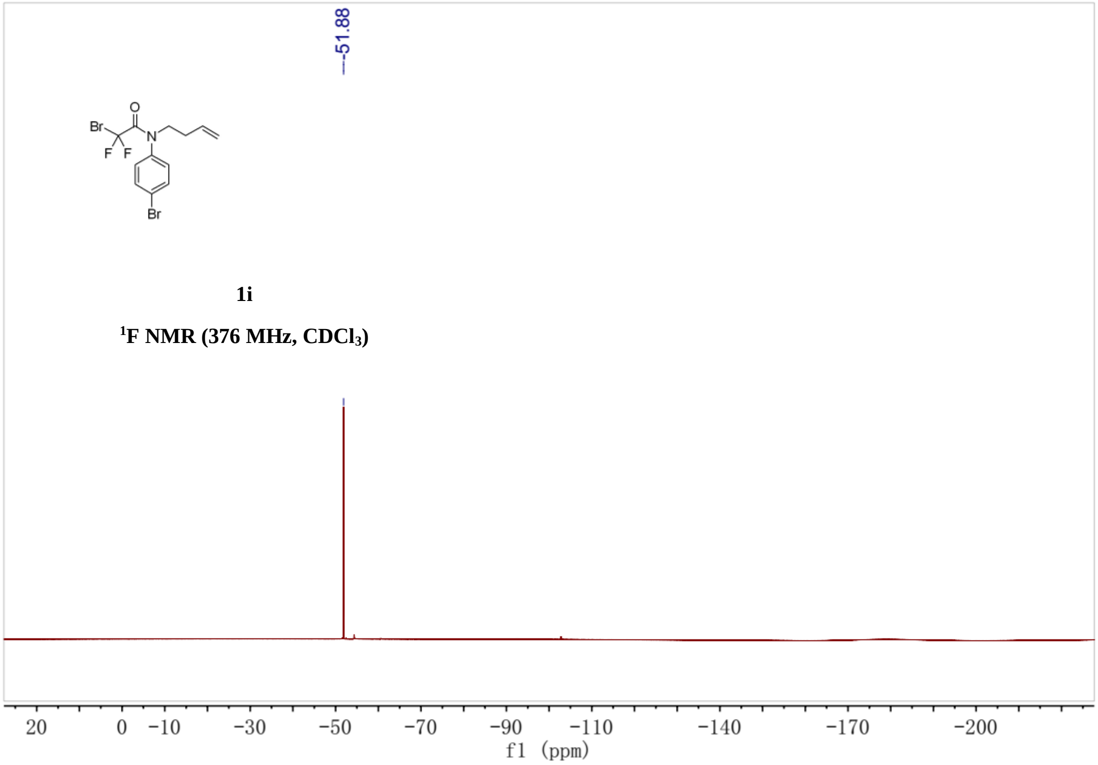

-51.88
1i
1F NMR (376 MHz, CDCl3)
20
0
−10
−30
−50
−70
−90
−110
−140
−170
−200
f1 (ppm)
